# Highly efficient H_2_S scavengers *via* thiolysis of positively-charged NBD amines†

**DOI:** 10.1039/d0sc01518k

**Published:** 2020-07-09

**Authors:** Ismail Ismail, Zhuoyue Chen, Lu Sun, Xiuru Ji, Haishun Ye, Xueying Kang, Haojie Huang, Haibin Song, Sarah G. Bolton, Zhen Xi, Michael D. Pluth, Long Yi

**Affiliations:** State Key Laboratory of Elemento-Organic Chemistry and Department of Chemical Biology, College of Chemistry, National Pesticide Engineering Research Center, Collaborative Innovation Center of Chemical Science and Engineering, Nankai University Tianjin 300071 China; State Key Laboratory of Organic–Inorganic Composites, Beijing Key Lab of Bioprocess, Beijing University of Chemical Technology (BUCT) Beijing 100029 China yilong@mail.buct.edu.cn; Tianjin Key Laboratory on Technologies Enabling Development of Clinical Therapeutics and Diagnostics (Theranostics), School of Pharmacy, Tianjin Medical University Tianjin 300070 China; Department of Chemistry and Biochemistry, Materials Science Institute, Knight Campus for Accelerating Scientific Impact, Institute of Molecular Biology, University of Oregon Eugene OR 97403 USA

## Abstract

H_2_S is a well-known toxic gas and also a gaseous signaling molecule involved in many biological processes. Advanced chemical tools that can regulate H_2_S levels *in vivo* are useful for understanding H_2_S biology as well as its potential therapeutic effects. To this end, we have developed a series of 7-nitro-1,2,3-benzoxadiazole (NBD) amines as potential H_2_S scavengers. The kinetic studies of thiolysis reactions revealed that incorporation of positively-charged groups onto the NBD amines greatly increased the rate of the H_2_S-specific thiolysis reaction. We demonstrate that these reactions proceed effectively, with second order rate constants (*k*_2_) of >116 M^−1^ s^−1^ at 37 °C for **NBD-S8**. Additionally, we demonstrate that **NBD-S8** can effectively scavenge enzymatically-produced and endogenous H_2_S in live cells. Furthering the biological significance, we demonstrate **NBD-S8** mediates scavenging of H_2_S in mice.

## Abstract 2

We demonstrate that positively-charged NBD amines can effectively scavenge biological H_2_S in live cells and in mice.

## Graphical abstract

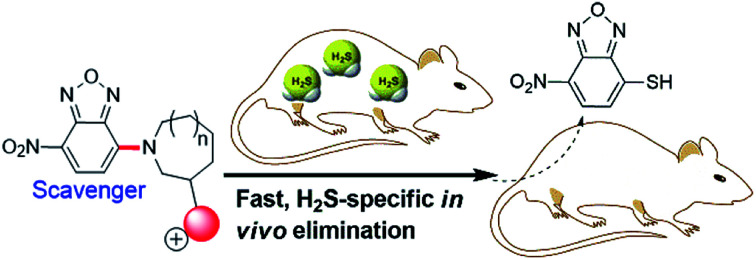

## Introduction

Hydrogen sulfide (H_2_S) has long been known as a toxic gas, but recent studies indicate that H_2_S has important physiological functions, leading to its inclusion as the third gasotransmitter along with nitric oxide (NO) and carbon monoxide (CO).^1–3^ Endogenous H_2_S is enzymatically generated by cystathionine γ-lyase (CSE), cystathionine-β-synthase (CBS) and 3-mercaptopyruvate sulfurtransferase (3-MST)/cysteine aminotransferase (CAT).^4^ H_2_S influences a wide range of physiological processes in mammals, ranging from vasorelaxation,^5^ cardioprotection,^6^ and neurotransmission^7^ to anti-inflammatory action^8^ and angiogenesis.^9^ Misregulation of H_2_S is associated with numerous diseases.^10,11^ Specially, low levels of endogenous H_2_S appears to exhibit pro-cancer effects, whereas higher concentrations of H_2_S can lead to cell apoptosis and have anti-cancer characteristics.^11^ Complementing the importance of H_2_S in mammals, H_2_S in bacteria and plants also plays many important functions.^12,13^ Due to its wide biodistribution and complex behaviors in many diseases, the physiological characters of H_2_S and the molecular mechanisms in which H_2_S is involved need further investigation. In addition, these factors support the development and refinement of advanced chemical tools that can visualize,^14^ scavenge^15^ or release^16^ H_2_S *in vivo* and in related complex environments.

Significant attention has focused on developing chemical tools for H_2_S detection and delivery,^14,16^ but much less effort has focused on reducing H_2_S levels in complex environments.^15,17^ Such H_2_S elimination could be achieved by either inhibition of H_2_S biosynthesis or by the selective scavenging of H_2_S. Though CBS inhibitors have been reported to show anti-tumor activity, many of CBS inhibitors target the pyridoxal-5′-phosphate (PLP) cofactor and therefore have low specificity or unwanted side effects.^11*b*^ Complementing these challenges, both CSE and 3-MST inhibitors are relatively underdeveloped.^11*a*^ Moreover, H_2_S can be produced from non-enzymatic processes,^14^ which suggests that enzymatic inhibitors can only perturb certain pools of biological H_2_S genesis. Recently, sulfonyl azides were reported as highly efficient H_2_S scavengers in buffers, enzymatic systems, and living biological environments.^15^ It should be noted that the H_2_S-mediated reduction of aryl azides to amines results in generation of sulfane sulfur and polysulfides,^15,18^ both of which are important reactive sulfur species that might complicate such scavenging.^19*a*^ Considering the *in vivo* biological complex,^19^ our goal was to develop efficient H_2_S scavengers utilizing different reaction mechanisms that not only scavenge H_2_S efficiently, but also do not generate other reactive sulfur species in the scavenging process with the long-term goal of applying these toward *in vivo* systems.

**Fig. 1:**
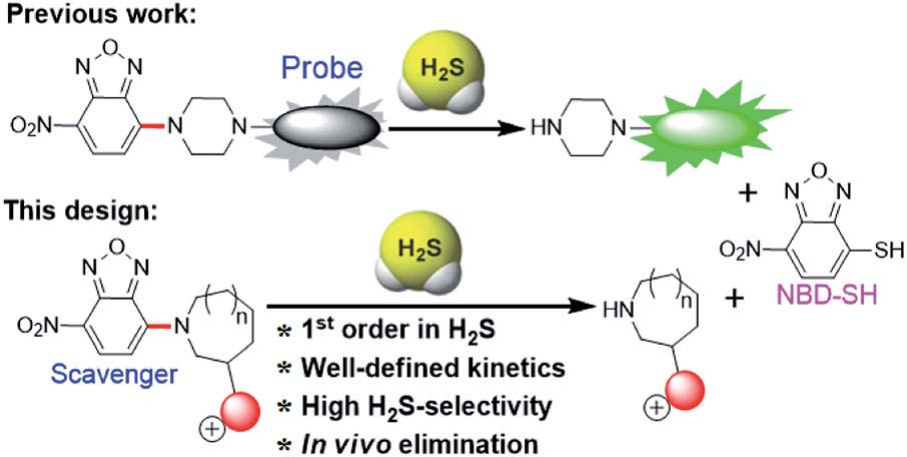
(top) The thiolysis of NBD amines used previously for H_2_S probe development. (bottom) Inclusion of cationic charge in the present system enables access to efficient H_2_S scavengers by thiolysis of NBD cyclic amines.

**Fig. 2:**
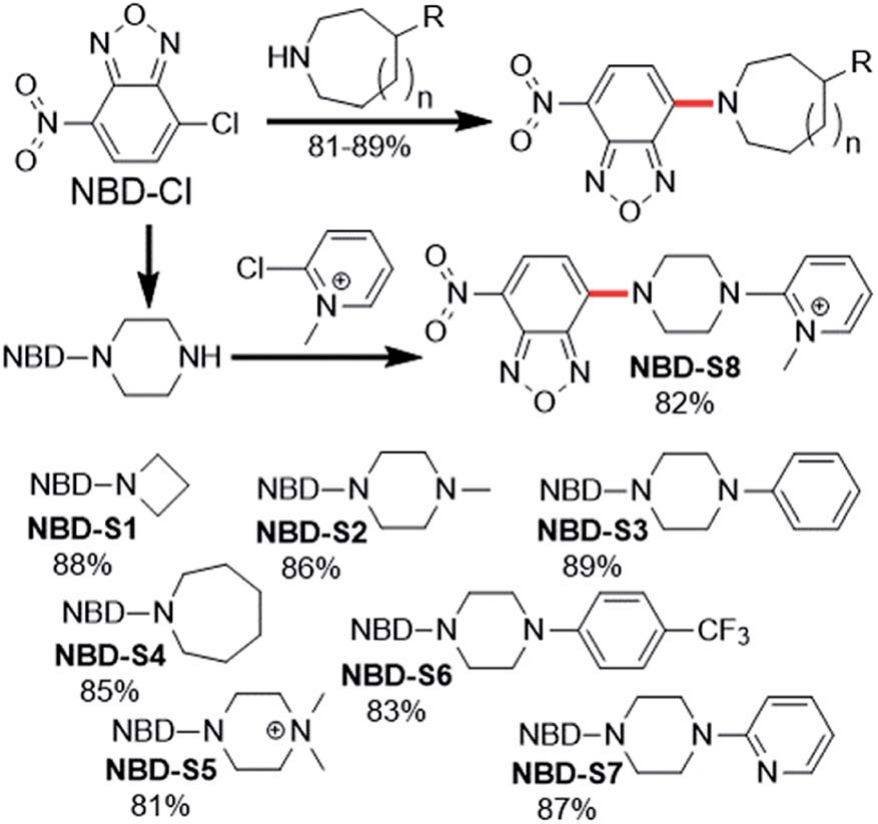
Synthesis of NBD amines **NBD-S1** to **NBD-S8**. The C–N bond cleaved by H_2_S-mediated thiolysis is highlighted in red in each structure.

A major challenge in the development of H_2_S scavengers is developing fast chemical reactions that effectively differentiate the reactivity of biological nucleophiles (*e.g.* biothiols) from H_2_S. To this end, we as well as others have previously reported the H_2_S-specific thiolysis of 7-nitro-1,2,3-benzoxadiazole (NBD) amine in 2013 (Fig. 1),^20^ which has enabled, amongst other applications, the development of near-infrared H_2_S probes for imaging of H_2_S *in vivo*.^20*c*^ To further advance the development of H_2_S scavengers, we report here that simple modifications of the NBD electrophiles can increase the reactivity of this platform to enable efficient H_2_S scavenging (Fig. 2) and apply these chemical tools to scavenge H_2_S in buffer, serum, cells, and mice. We believe that the present H_2_S scavengers could be useful tools to study H_2_S biology as well as potential therapeutic agents in the future.

## Results and discussion

Compounds **NBD-S1** to **NBD-S8** were synthesized from commercially available reagents by simply coupling **NBD-Cl** to the desired amine or by reacting NBD-piperizine with 2-chloro-1-methylpyridinium salt (Fig. 2). Compound identity was confirmed by ^1^H and ^13^C NMR spectroscopy and HRMS. All scavengers were prepared in high yield in either one- or two-step syntheses, which readily enables access to large quantities of each scavenger.

**Fig. 3:**
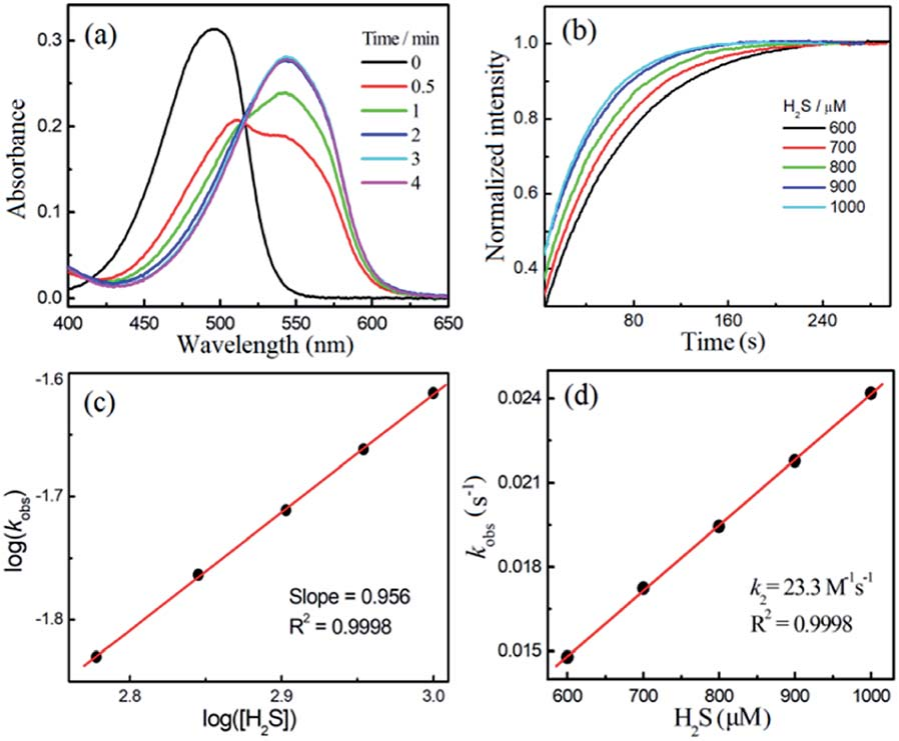
(a) Time-dependent absorbance spectra of 10 μM **NBD-S2** towards 1 mM H_2_S in PBS buffer (50 mM, pH = 7.4, containing 2% DMSO) at 25 °C. (b) Time-dependent normalized signals at 540 nm of 10 μM **NBD-S2** towards different concentrations of H_2_S. (c) The reaction order of H_2_S was determined as the slope of plots of log(*k*_obs_) *versus* log([H_2_S]). (d) The *k*_2_ was determined as the slope of plots of *k*_obs_*versus* [H_2_S].

To measure the rate of the prepared compounds with H_2_S, we monitored the optical response of **NBD-S1–NBD-S8** (5–10 μM) after addition of H_2_S (6–600 equiv., using Na_2_S) in PBS buffer (pH 7.4) at 25 °C. An example of the observed reactivity is shown for **NBD-S2** (Fig. 3a), in which the starting absorbance peak at 490 nm from the NBD moiety shifts to 540 nm after reaction with H_2_S due to formation of **NBD-SH**.^20*b*^ The reaction kinetics were monitored by measuring absorbance data at 540 nm or 490 nm (Fig. S1†) and showed a clear isosbestic point at 510 nm, which is consistent with a clean overall transformation. The pseudo-first-order rate constant, *k*_obs_ was determined by fitting the intensity data to a single exponential function (Fig. 3b). Plotting log(*k*_obs_) *versus* log([H_2_S]) confirmed a first-order dependence in H_2_S (Fig. 3c). The linear fitting between *k*_obs_ and H_2_S concentrations gives the second-order rate constant (*k*_2_) of 23.3 M^−1^ s^−1^ (Fig. 3d).

Using the above method, the H_2_S-mediated thiolysis rates for the prepared NBD amines were measured, and these data are summarized in Table 1. The thiolysis rates for the four, six, and seven-membered ring amines (**NBD-S1** to **NBD-S4**) are significantly different, with the presence of the second amine group in the ring appearing to be particular important. We hypothesized that this increased rate could be due to protonation of the piperizine nitrogen at physiological pH (*e. g.*: the p*K*_a_ of 1-methyl piperizine is ∼9).^21^ To verify this hypothesis, compounds **NBD-S5** to **NBD-S8** with the functionlized second amine were further tested. As expected, alkylation of the piperizine nitrogen (**NBD-S5**) or inclusion of a positively charged pyridinium (**NBD-S8**) significantly increased the thiolysis reaction rates. For example, *k*_2_ values for cationic **NBD-S5** and **NBD-S8** derivatives were 70.2 M^−1^ s^−1^ and 76.2 M^−1^ s^−1^ at 25 °C, respectively, which are significantly faster than the *k*_2_ values of the neutral **NBD-S2** and **NBD-S7** analogues. To further support these measurements, we also performed kinetic analysis using fluorescence methods (Fig. S9 and S10†) for **NBD-S5** and **NBD-S8**, which afforded *k*_2_ values of 71.6 M^−1^ s^−1^ and 78.3 M^−1^ s^−1^, respectively, which are consistent with those from absorbance measurements. We also measured *k*_2_ for **NBD-S8** at 37 °C, which yielded a significantly higher value of 116.1 M^−1^ s^−1^ (Fig. S11†), and suggests that this compound can scavenge H_2_S efficiently at physiological temperatures. The formation of the amine product during the thiolysis reaction was confirmed by HPLC and HRMS analysis (Fig. S12 and S13†). Taken together, these data suggest that **NBD-S5** and **NBD-S8** are highly reactive reagents toward H_2_S clearance.

**Table 1:** Kinetic parameters of the thiolysis of NBD amines at 25 °C

	**NBD-S1**	**NBD-S2**	**NBD-S3**	**NBD-S4**	**NBD-S5**	**NBD-S6**	**NBD-S7**	**NBD-S8**
k_obs_^a^/s^−1^	4.2 × 10^−3^	2.4 × 10^−2^	2.7 × 10^−2^	N.D.^e^	9.1 × 10^−3^	2.2 × 10^−2^	3.6 × 10^−2^	9.3 × 10^−3^
([H_2_S]^b^)	6.0 mM	1.0 mM	1.5 mM		120 μM	1.2 mM	2.0 mM	120 μM
Slope^c^	0.937	0.956	1.039	N.D.	0.898	1.066	1.048	0.979
*k*_2_ d/M^−1^ s^−1^	0.67	23.3	18.0	< 0.01	70.2	19.7	18.8	76.2

a *k*_obs_: the value was obtained by fitting the time-dependent spectra data with single exponential function.

b [H_2_S]: the H_2_S concentration used at the determination of the *k*_obs_ value.

c Slope: the slope value of the linear fitting of log(*k*_obs_) *versus* log([H_2_S]).

d *k*_2_: the slope value of the linear fitting of *k*_obs_*versus* [H_2_S].

e N.D.: no detection due to the very slow reaction.

**Fig. 4:**
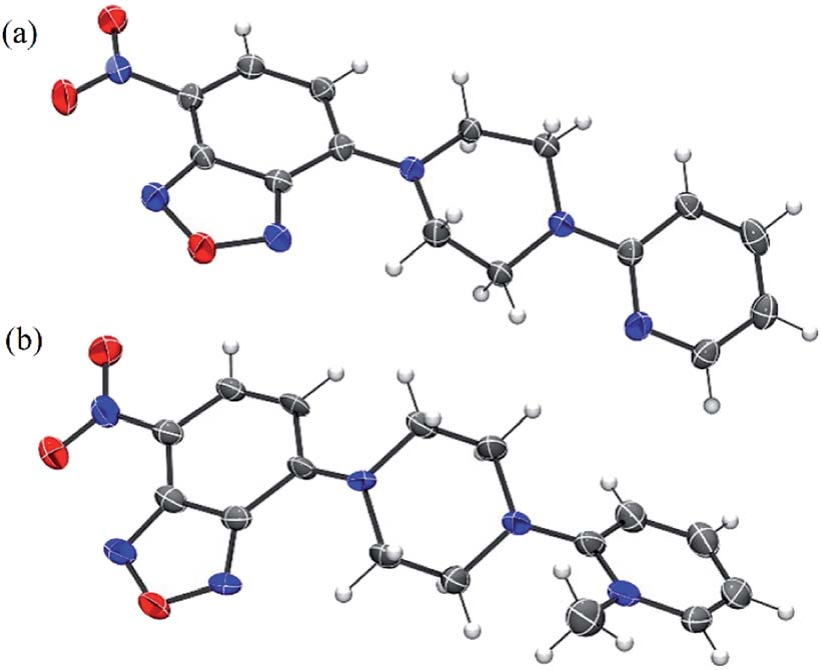
ORTEP diagram (50% ellipsoid) for the molecular structure of **NBD-S7** (a) and **NBD-S8** (b). The I^−^ counterion in (b) is omitted for clarity.

We also obtained crystals suitable for single-crystal X-ray diffraction studies of **NBD-S7** and **NBD-S8** (Fig. 4). In the solid state, the piperazinyl heterocycles in **NBD-S7** and **NBD-S8** are in twist-boat and chair conformations, respectively. Prior work with electrophilic piperazine-containing cyanine GSH probes has shown that the chair conformation is favorable for the thiolysis reaction,^22*a*^ which may help explain the increased reaction rate of **NBD-S8** when compared to **NBD-S7**. In addition, the positive charge of **NBD-S8** may favor reaction with anionic HS^−^, and prior work with probes for F^−^ has shown increased rates for cationic versions of probes when compared to neutral counterparts.^22*b*^

We also evaluated the stability, water-solubility, and selectivity of **NBD-S5** and **NBD-S8**. The plots of absorbance intensity against the scavenger concentration (up to 100 μM) were linear (Fig. S14 and S15†), suggesting the good solubility of **NBD-S5** and **NBD-S8** in PBS buffer (50 mM, pH = 7.4, containing 2% DMSO). Both **NBD-S5** and **NBD-S8** were shown to be stable in PBS buffer, with no decomposition over 2 days (Fig. S16†). The high selectivity of the NBD amines toward H_2_S over biothiols were validated by HPLC analysis (Fig. S17†), suggesting the thiolysis of the NBD amines is highly H_2_S-specific.^20*c*^ We propose that this high selectivity may originate from the intrinsic low thiolysis reactivity for C–N bond cleavage (*e.g.***NBD-S1**), and that certain structural factors, such as the piperazine chair conformation and positively-charged group, may contribute to the faster reactivity with HS^−^ (*e.g.***NBD-S5** and **NBD-S8**). Therefore, **NBD-S5** and **NBD-S8** were further employed as H_2_S scavengers in the following studies.

**Fig. 5:**
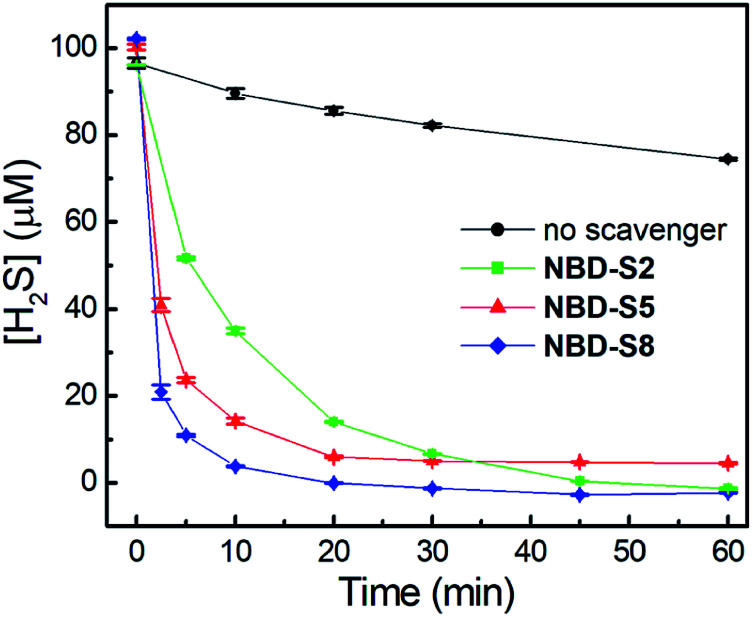
H_2_S-scavenging curves of NBD amines or no scavenger in PBS buffer (pH 7.4) at 25 °C. Three scavengers (**NBD-S2**, **NBD-S5**, and **NBD-S8**, 110 μM) were added to H_2_S solutions (100 μM). The H_2_S concentrations were measured using MBA. The results are expressed as mean ± S.D. (*n* = 3).

We first tested the efficiency of H_2_S clearance by NBD amines in PBS buffer (50 mM, pH 7.4), using the methylene blue assay (MBA)^23^ to quantify H_2_S concentrations (Fig. S18–S21†). The time-dependent H_2_S concentrations in the absence or presence of **NBD-S2**, **NBD-S5**, or **NBD-S8** are shown in Fig. 5. The concentration of H_2_S slowly decreasd over time, which matches previous observations.^24^ Under these conditions, both **NBD-S5** and **NBD-S8** can scavenge more than 50% H_2_S within the first 2 min. Additionally, **NBD-S8** can scavenge more than 90% H_2_S within 5 min, suggesting it is a highly efficient H_2_S scavenger in aqueous buffer (pH 7.4). Based on these parameters, we used **NBD-S8** for subsequent applications. Considering H_2_S is a toxic gas,^2*b*^ we tested the ability of the **NBD-S8** solution to remove H_2_S in gaseous sample (Fig. S22†).^25^ The color change of **NBD-S8** solution and MBA tests of the solution clearly suggested **NBD-S8** could efficiently scavenge H_2_S when the H_2_S-containing gas was passed through the **NBD-S8** solution.

**Fig. 6:**
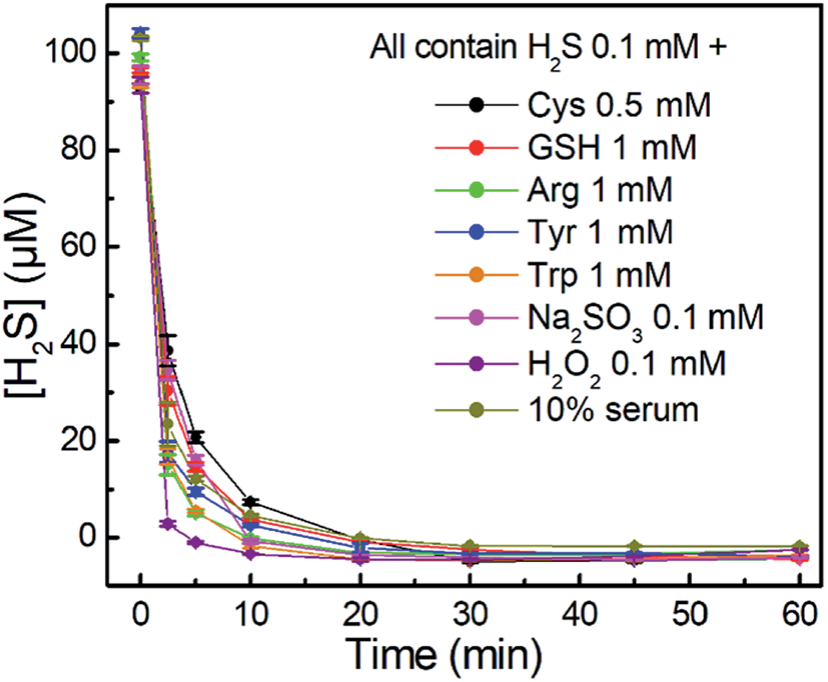
Time-dependent H_2_S-scavenging curves of **NBD-S8** (110 μM) in the presence of other species (inset) and H_2_S (100 μM). The H_2_S concentrations were measured using MBA. The results are expressed as mean ± S.D. (*n* = 3).

We further investigated the time-dependent H_2_S-scavenging ability of **NBD-S8** in the presence of biologically relevant species. As shown in Fig. 6, the results suggest that the scavenging of H_2_S *via***NBD-S8** is not significantly influenced by the presence of millimolar biothiols or other species. The scavenging of H_2_S in 10% fetal bovine serum (FBS) was also efficient. For all tested samples, **NBD-S8** could scavenge more than 90% H_2_S within first 10 min. These data suggest that **NBD-S8** should be an efficient H_2_S scavenger in complex biological-related systems.

Then we tested whether **NBD-S8** could scavenge H_2_S in live cells. We first tested the cytotoxicity of **NBD-S8** by the MTT assay using HeLa cells which contain nearly no endogenous H_2_S. The scavenger did not show cytotoxicity up to 50 μM concentration (Fig. S23a†). Furthermore, the cell viability in the presence of 100 μM and 200 μM **NBD-S8** is still greater than 90% and 80%, respectively. These results suggest that **NBD-S8** is biocompatible for scavenging H_2_S in living cells. To confirm that the thiolysis products of **NBD-S8** (**NBD-SH** and **S8-II**) are not cytotoxic, we evaluated the cytotoxicity in HeLa cells for 24 hours. **NBD-SH** did not show any significant cytotoxicity below 25 μM (Fig. S23b†). The cell viability in the presence of 25 μM and 100 μM **S8-II** is still greater than 90% and 80%, respectively (Fig. S23c†). We note that under physiological conditions, the concentration of free H_2_S is significantly lower than 1 μM,^19*a*^ meaning that we do not expect to generate high micromolar levels of **NBD-SH** under normal scavenging conditions.

**Fig. 7:**
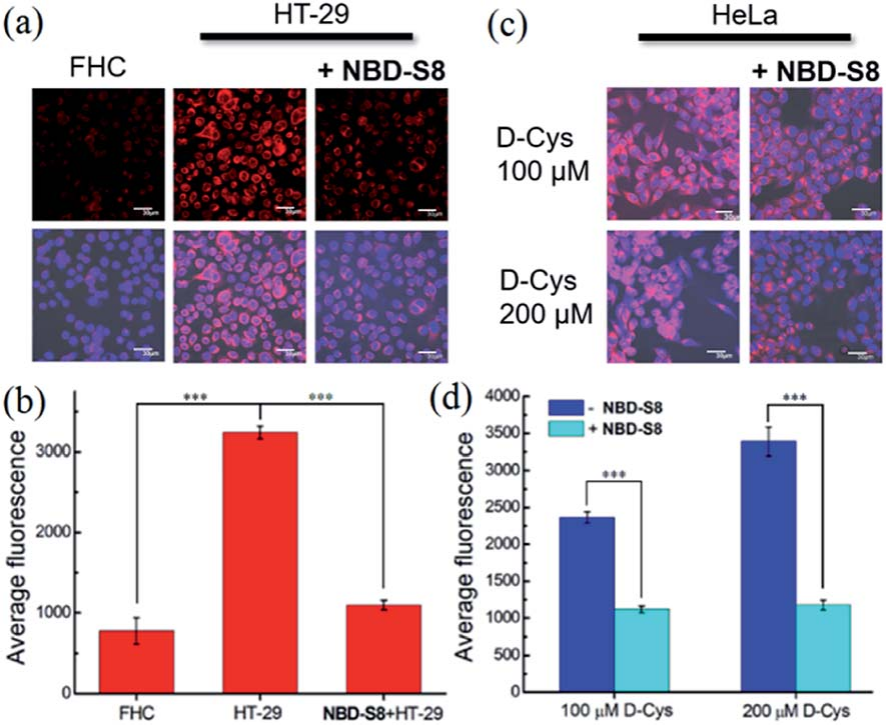
H_2_S scavenged by **NBD-S8** in live cells. (a) H_2_S levels in FHC and HT-29 cells were visualized by the fluorescence probe **Cy7-NBD** (10 μM), and the HT-29 cells were pre-treated with or without 100 μM **NBD-S8**. The nuclei were stained with DAPI. Red fluorescence images (top) and overlap of blue and red fluorescence images (bottom) are shown. (b) Average fluorescence of *N* = 3 fields of cell images in (a). (c) d-Cys-induced H_2_S levels in HeLa cells were visualized by **Cy7-NBD** (10 μM), and the HeLa cells were treated with or without 100 μM **NBD-S8**. Overlap of blue and red fluorescence images are shown. (d) Average fluorescence of *N* = 3 fields of cell images in (c). All emissions were collected at blue (450–550 nm, excitation at 405 nm) and red (650–750 nm, excitation at 647 nm) channels for DAPI and **Cy7-NBD**, respectively. Error bars are means ± S.D. ***p < 0.001. Scale bar, 30 μm.

To measure H_2_S levels, we use the H_2_S-responsive fluorescence probe **Cy7-NBD** (Fig. S24†).^20*c*^ We used HT-29 (human colonic carcinoma cells) cells for these experiments due to recent reports that colorectal cancer cell lines exhibit increased H_2_S production,^11*b*^ which is likely due to the increased angiogenesis needed for cancer cell growth and proliferation.^9^ We used non-cancerous FHC (human fetal normal colonic mucosa) cells as a negative control, which we expected to lower basal H_2_S levels. To test whether **NBD-S8** could function as an efficient H_2_S scavenger, we compared the fluorescence response from **Cy7-NBD** in FHC cells, HT-29 cells, and HT-29 cells treated with **NBD-S8** (Fig. 7a and S25†). We observed a significantly higher response from **Cy7-NBD** in HT-29 cells when compared to FHC cells, as expected. Upon pre-treatment of HT-29 cells with 100 μM **NBD-S8** for 10 min, the fluorescence signals in HT-29 cells significantly decreased (Fig. 7b), which supports that **NBD-S8** can efficiently scavenge endogenous H_2_S in HT-29 cells.

To further support the ability of **NBD-S8** to scavenge H_2_S in live cells, we also measured H_2_S levels in HeLa cells treated with d-Cys (100 μM or 200 μM), which can increase H_2_S biosynthesis from the 3-MST pathway.^4*b*,26^ As expected, we observed a strong red fluorescence signal from the d-Cys-treated cells in the absence of **NBD-S8** (Fig. 7c and S26†).^26^ In contrast, co-incubation of d-Cys treated HeLa cells with 100 μM **NBD-S8**, the cells displayed significantly lower fluorescence than that of the control cells (Fig. 7d). Taken together with the data in HT-29 cells, these data suggest that **NBD-S8** can be applied to efficiently scavenge enzymatically-produced and endogenous H_2_S in live cells.

**Fig. 8:**
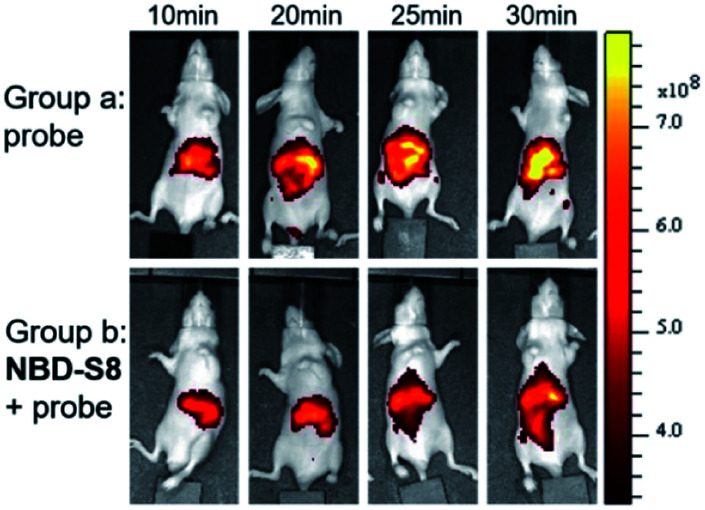
Representative fluorescence images of mice which were treated with **NBD-S8** and **Cy7-NBD***via* tail vein injection. In (group a) mice were injected with **Cy7-NBD** (150 μM, 200 μL) only; whilst in (group b) mice were pretreated with **NBD-S8** (100 μM, 200 μL) for 10 min, and then **Cy7-NBD**.

Encouraged by these results, we further examined the feasibility of **NBD-S8** for H_2_S clearance in normal nude mice again using **Cy7-NBD** as an H_2_S reporter. Briefly, mice were intraperitoneally injected with probe only (negative control), Na_2_S followed by probe (positive control), or Na_2_S followed by **NBD-S8** for 10 min and then probe. The mice were imaged using an IVIS spectrum imaging system (PerkinElmer, USA) with a 740 nm excitation filter. As expected, the mice injected with Na_2_S and **NBD-S8** displayed weaker fluorescence signal than that of the mice injected with Na_2_S (Fig. S27†). These results implied that **NBD-S8** still functions H_2_S clearance *in vivo*, and its feasibility to scavenge endogenous H_2_S in live mice was further examined. To investigate the ability to scavenge endogenous H_2_S, **NBD-S8** was administered to 3 mice *via* tail vein injection. After 10 min, probe **Cy7-NBD** was injected to visualize the level of endogenous H_2_S. As a control group, another 3 mice were injected with **Cy7-NBD** only. As shown in Fig. 8, time-dependent fluorescence increase was detected primarily in the liver of mice (group a), which is consistent with previous results from tissue imaging.^20*c*^ However, mice in (group b) exhibited comparatively lower fluorescence at each corresponding time point when compared to group a (Fig. S28†). These results preliminarily demonstrate that **NBD-S8** can scavenge endogenous H_2_S at the organism level.

## Conclusions

In summary, we have developed H_2_S scavengers based on the thiolysis of NBD amines. **NBD-S8** shows clear reaction kinetics toward H_2_S with 2^nd^ order rate constants of up to 116 M^−1^ s^−1^ at 37 °C and high selectivity toward H_2_S over other biologically relevant species. **NBD-S8** can efficiently scavenge H_2_S in aqueous buffer, serum, gaseous sample, and live cells. Additionally, the high selectivity and reactivity of **NBD-S8** toward H_2_S enables efficient H_2_S scavenging in live animals. Moreover, **NBD-S8** showed better stability in 5 mM GSH containing PBS buffer (pH 7.4) than that of *p*-toluenesulfonyl azide (Fig. S29†), a recently reported H_2_S scavenger. Considering *in vivo* biological complexity,^19^ we propose that H_2_S scavengers *via* the H_2_S-specific thiolysis of NBD amines may represent an emerging class of efficient scavengers for investigating H_2_S in complex biological systems.

## Author contributions

L. Y., M. D. P., Z. X. supervised the work. I. I. synthesized the compounds; Z. C., H. Y., X. K. tested the kinetics and *in vitro* H_2_S scavenging; L. S., X. J., H. H. performed the bioimaging tests; I. I., H. S. solved crystal structures; S. G. B., X. J. tested the cytotoxicity; L. Y., M. D. P. wrote the manuscript; all authors involved in checking the manuscript and data.

## Ethical statement

All animal procedures were performed in accordance with the Guidelines for Care and Use of Laboratory Animals of Tianjin Medical University and approved by the Animal Ethics Committee of Tianjin Medical University.

## Conflicts of interest

The authors declare no competing financial interests.

## Supplementary Material

SC-011-D0SC01518K-s001

SC-011-D0SC01518K-s002
