# Supplementary material for: Highly efficient H2S scavengers via thiolysis of positively-charged NBD amines
Source: Chem Sci. 2020 Jul 9;11(30):7823–8. doi: 10.1039/d0sc01518k (PMC8163142; doi:10.1039/d0sc01518k)
Supplement: SC-011-D0SC01518K-s001 [file SC-011-D0SC01518K-s001.pdf]

Electronic Supplementary Material (ESI) for Chemical Science.
This journal is © The Royal Society of Chemistry 2020

Supporting Information

Highly efficient H_2S scavengers via thiolysis of positively-charged NBD amines

Ismail Ismail,[‡a] Zhuoyue Chen,[‡b] Lu Sun,[‡c] Xiuru Ji,[c] Haishun Ye,[b] Xueying Kang,[b] Haojie Huang,[b] Haibin Song,[a] Sarah G. Bolton,[d] Zhen Xi,[a] Michael D. Pluth[d] and Long Yi*[b]

[a]State Key Laboratory of Elemento-Organic Chemistry and Department of Chemical Biology, College of Chemistry, National Pesticide Engineering Research Center, Collaborative Innovation Center of Chemical Science and Engineering, Nankai University, Tianjin, China.

[b]State Key Laboratory of Organic-Inorganic Composites and Beijing Key Lab of Bioprocess, Beijing University of Chemical Technology (BUCT), Beijing 100029, China.

[c]Tianjin Key Laboratory on Technologies Enabling Development of Clinical Therapeutics and Diagnostics (Theranostics), School of Pharmacy, Tianjin Medical University, Tianjin 300070, China.

[d]Department of Chemistry and Biochemistry, Materials Science Institute, Institute of Molecular Biology, University of Oregon, Eugene, OR 97403, USA.

[‡]These authors contributed equally to this work.

Table of contents

1. Synthesis	2
2. Spectra tests and HPLC analysis	7
3. X-ray crystallography studies	8
4. H_2S scavenging tests	14
5. Cell culture, MTT assay and bioimaging	15
6. Optical *in vivo* imaging	17
7. Supporting figures	18
8. Supporting NMR and MS spectra	38
9. Supporting reference	55

1. Synthesis

All chemicals and solvents used for organic synthesis were purchased from commercial suppliers and applied directly in the experiments without further purification. The progress of the reaction was monitored by TLC on pre-coated silica plates (Merck 60F-254, 250 μm in thickness), and spots were visualized by basic $KMnO_4$, UV light or iodine. Merck silica gel 60 (100-200 mesh) was used for general column chromatography purification. ^{1}H NMR and ^{13}C NMR spectra were recorded on a Bruker 400 spectrometer. High-resolution mass spectra (HRMS) were obtained on an Agilent 6540 UHD Accurate-Mass Q-TOFLC/MS or Varian 7.0 T FTICR-MS. The single-crystal X-ray diffraction data set was collected and tested on a Rigaku 007 Saturn 70 (Mo target) diffractometer.

General procedure:

The NBD-amines (**NBD-S1** to **NBD-S7**) were synthesized by a general literature procedure.[1] To a solution of 4-chloro-7-nitrobenzofurazan (NBD-Cl, 1.1 equiv) in dichloromethane (10 mL) was added corresponding amines (1.0 equiv) followed by *N*,*N*-diisopropylethylamine (DIPEA, 1.5 equiv) or triethylamine (TEA, 2.0 equiv). The reaction mixture was stirred for 3-12 h at room temperature under an inert atmosphere. After completion of reaction, the solvent was removed in vacuo, and the resulted residue was purified by column chromatography by eluting with methanol/dichloromethane to give the NBD-amines.

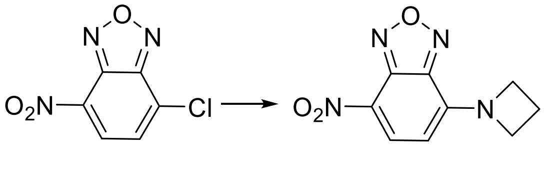

NBD-S1

Synthesis of NBD-S1:

Yielded an orange-red solid product (88%). ^{1}H NMR (400 MHz, DMSO-d_6) δ 8.45 (d, J = 9.2 Hz, 1H), 6.03 (d, J = 9.2 Hz, 1H), 4.77 (bs, 2H), 4.42 (bs, 2H), 2.52–2.61 (m, 2H). ^{13}C NMR (101 MHz, DMSO-d_6) δ 145.2, 144.6, 143.6, 136.4, 119.1, 99.4, 56.4, 53.0, 16.5. HRMS: $[M+H]^+$ calcd. for $C_9H_9N_4O_3^+$: 221.0669; found: 221.0667.

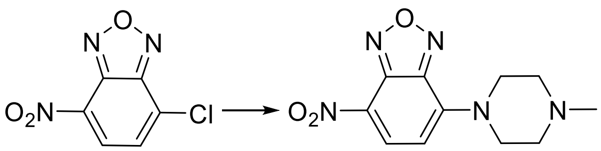

NBD-S2

Synthesis of NBD-S2:

Yielded a dark red solid product (86%). ^{1}H NMR (400 MHz, DMSO-d_6) δ 8.47 (d, J = 9.2 Hz, 1H), 6.68 (d, J = 9.2 Hz, 1H), 4.13 (bs, 4H), 2.61-2.53 (m, 4H), 2.25 (s, 3H). ^{13}C NMR (101 MHz, DMSO-d_6) δ 145.2, 144.7, 136.2, 121.0, 103.5, 54.1, 49.2, 45.2. HRMS: $[M+Na]^+$ calcd. for $C_{11}H_{13}N_5O_3Na^+$: 286.0911; found: 286.0915.

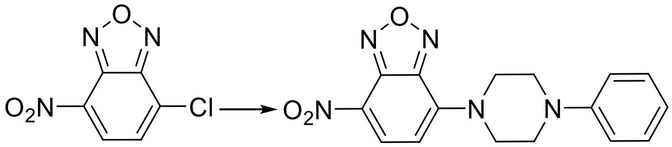

NBD-S3

Synthesis of NBD-S3:

Yielded a red solid product (89%). ^{1}H NMR (400 MHz, DMSO-d_6) δ 8.49 (d, J = 9.2 Hz, 1H), 7.25 (t, J = 8.0 Hz, 2H), 6.98 (d, J = 8.0 Hz, 2H), 6.81 (t, J = 7.2 Hz, 1H), 6.68 (d, J = 9.2 Hz, 1H), 4.29 (bs, 4H), 3.48 (bs, 4H). ^{13}C NMR (101 MHz, DMSO-d_6) δ 150.0, 145.3, 144.8, 136.3, 129.1, 121.1, 119.0, 115.1, 103.4, 49.0, 47.3. HRMS: $[M-H]^-$ calcd. for $C_{16}H_{14}N_5O_3^-$: 324.1103; found: 324.1100.

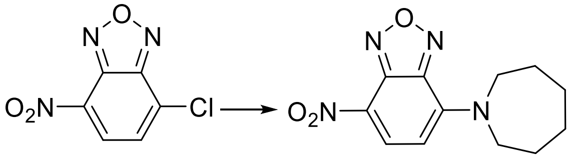

NBD-S4

Synthesis of NBD-S4:

Yielded a red solid product (85%). ^{1}H NMR (400 MHz, DMSO-d_6) δ 8.45 (d, J = 9.2 Hz, 1H), 6.49 (d, J = 9.2 Hz, 1H), 4.35 (bs, 2H), 3.91 (bs, 2H), 1.87 (bs, 4H), 1.56 (bs, 4H). ^{13}C NMR (101 MHz, DMSO-d_6) δ 145.6, 144.8, 144.2, 136.1, 119.7, 101.9, 53.8, 52.5, 27.7, 25.7, 24.9. HRMS: $[M+H]^+$ calcd. for $C_{12}H_{15}N_4O_3^+$: 263.1139; found: 263.1136.

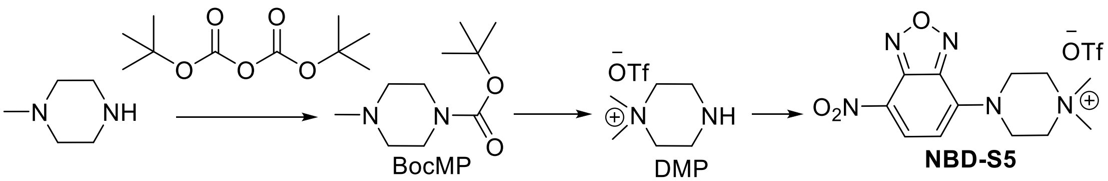

Synthesis of NBD-S5:

NBD-S5 was synthesized by the following three steps, according to a literature procedure.[2]

Tert-butyl 4-methylpiperazine-1-carboxylate (BocMP):

To the solution containing 1-methylpiperazine (0.5 g, 4.99 mmol) in tetrahydrofuran (6 mL) was added di-tert-butyl dicarbonate (1.72 mL, 7.49 mmol) under 0 °C, and the resulted mixture was stirred at room temperature for 2 h. The organic phase was removed under reduced pressure, and the resulted residue was purified by silica gel column chromatography to yield product BocMP (0.949 g, 95%). ^{1}H NMR (400 MHz, $CDCl_3$) δ 3.47 (t, J = 4.8 Hz, 4H), 2.39 (t, J = 4.8 Hz, 4H), 2.32 (s, 3H), 1.46 (s, 9H). ^{13}C NMR (101 MHz, $CDCl_3$) δ 154.8, 79.9, 77.4, 54.8, 46.1, 28.5.

1,1-Dimethylpiperazinium trifluoromethanesulfonate (DMP):

Methyl triflate (0.409 g, 2.49 mmol) was dropwise added to the solution of 4-methyl-piperazine-1-carboxylic acid tertbutyl ester (0.5 g, 2.49 mmol) in dry dichloromethane (10 mL) and stirred at room temperature over 1 h. Then the mixture was treated with triflic acid (0.599 g, 3.99 mmol) and allowed to stir for one hour. The organic phase was removed under reduced pressure, and the resulted residue was washed by 10 mL methanol to produce the white solid precipitate DMP (0.540 g, 82%). ^{1}H NMR (400 MHz, DMSO-d_6) δ 9.05 (br, 1H), 3.57 (bs, 4H), 3.54 (s, 6H), 3.24 (bs, 4H). ^{13}C NMR (101 MHz, DMSO-d_6) δ 120.9 (q, CF_3, $J_{C\text{-}F}$ = 322 Hz), 58.1, 51.8, 37.8.

DMP (0.2 g, 0.76 mmol) and TEA (210.2 μL, 1.15 mmol) were mixed in dichloromethane (10 mL) followed by dropwise addition of NBD-Cl (0.181 g, 0.91 mmol) in dichloromethane (1 mL). The mixture was stirred at room temperature for 14 h. After completion of reaction, the solvent was removed in vacuo, and the resulted residue was purified by column chromatography to give a brown-black solid product **NBD-S5** (0.262 g, 81%). ^{1}H NMR (400 MHz, DMSO-d_6) δ 8.62 (d, J = 8.8 Hz, 1H), 6.81 (d, J = 9.2 Hz, 1H), 4.44 (bs, 4H), 3.69 (bs, 4H), 3.26 (s, 6H). ^{13}C NMR (101 MHz, DMSO-d_6) δ 144.8,

144.6, 144.5, 136.1, 123.3, 105.0, 59.7, 50.9, 43.1. HRMS: $[M]^+$ calcd. for $C_{12}H_{16}N_5O_3^+$: 278.1248; found: 278.1250.

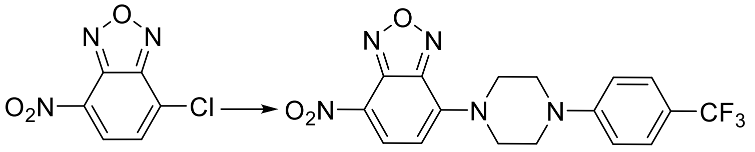

NBD-S6

Synthesis of NBD-S6:

Yielded a red solid product (83%). ^{1}H NMR (400 MHz, DMSO-d_6) δ 8.46 (d, J = 8.8 Hz, 1H), 7.54 (d, J = 8.0 Hz, 2H), 7.06 (d, J = 8.4 Hz, 2H), 6.61 (d, J = 8.8 Hz, 1H), 4.32 (bs, 4H), 3.69 (bs, 4H). ^{13}C NMR (101 MHz, DMSO-d_6) δ 152.0, 145.2, 144.6, 136.2, 129.1, 126.4, 126.3, 126.2, 123.7, 121.0, 117.6 (q, CF_3, $J_{C\text{-}F}$ = 32 Hz), 113.2, 103.0, 48.5, 45.4. HRMS: $[M+Na]^+$ calcd. for $C_{17}H_{14}F_3N_5O_3Na^+$: 416.0941; found: 416.0948.

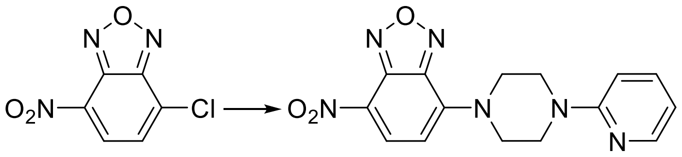

NBD-S7

Synthesis of NBD-S7:

Yielded a red solid product (87%). ^{1}H NMR (400 MHz, DMSO-d_6) δ 8.51 (d, J = 8.8 Hz, 1H), 8.11–8.22 (m, 1H), 7.58 (t, J = 7.6 Hz, 1H), 6.84 (d, J = 8.4 Hz, 1H), 6.76–6.60 (m, 2H), 4.29 (bs, 4H), 3.84 (bs, 4H). ^{13}C NMR (101 MHz, DMSO-d_6) δ 158.1, 147.6, 145.5, 144.8, 137.7, 136.3, 120.9, 113.2, 106.8, 103.1, 48.8, 43.4. HRMS: $[M+H]^+$ calcd. for $C_{15}H_{15}N_6O_3^+$: 327.1200; found: 327.1199.

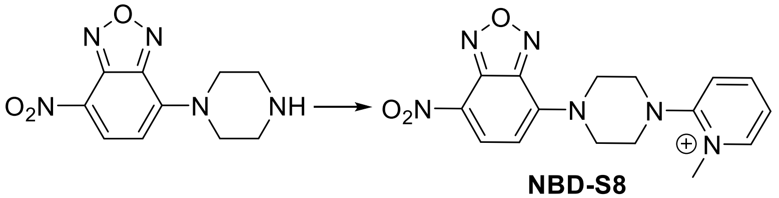

NBD-S8

Synthesis of NBD-S8:

2-Chloro-1-methylpyridin-1-ium iodide (0.1 g, 0.39 mmol) and TEA (108.8 µL, 0.78 mmol) were mixed in dichloromethane (10 mL), followed by dropwise addition of NBD-piprazine[1] (0.098 g, 0.39 mmol) in dichloromethane (1 mL).[3] The mixture was stirred at room temperature for 12 h. After completion of the reaction, the solvent was removed in

vacuo, and the resulted residue was purified by column chromatography to give a dark red solid product **NBD-S8** (0.15 g, 82%). ^{1}H NMR (400 MHz, DMSO-d_6) δ 8.67 (d, J = 5.6 Hz, 1H), 8.54 (d, J = 8.8 Hz, 1H), 8.37 (t, J = 7.6 Hz, 1H), 7.73 (d, J = 8.4 Hz, 1H), 7.57 (t, J = 6.4 Hz, 1H), 6.75 (d, J = 8.8 Hz, 1H), 4.39 (bs, 4H), 4.19 (s, 3H), 3.74 (bs, 4H). ^{13}C NMR (101 MHz, DMSO-d_6) δ 157.6, 145.3, 145.0, 144.9, 144.8, 144.7, 136.3, 121.7, 119.5, 119.3, 103.8, 48.9, 48.4, 44.3. HRMS: $[M]^+$ calcd. for $C_{16}H_{17}N_6O_3^+$: 341.1357; found: 341.1362.

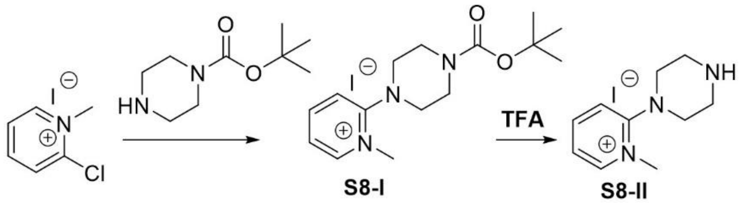

Synthesis of S8-II:

2-Chloro-1-methylpyridin-1-ium iodide (0.2 g, 0.78 mmol) and DIPEA (269.5 µL, 1.56 mmol) were mixed in dichloromethane (8 mL), followed by dropwise addition of 1-boc-piprazine (0.16 g, 0.86 mmol) in dry dichloromethane (2 mL). The mixture was stirred at room temperature for 12 h. After completion of the reaction, the solvent was removed in vacuo and the residue was subjected for column chromatography by eluting with methanol/dichloromethane to provide a white solid (0.17 g, 53.6%). ^{1}H NMR (400 MHz, DMSO-d_6) δ 8.70 (d, J = 6.4 Hz, 1H), 8.36 (t, J = 8.4 Hz, 1H), 7.67 (d, J = 8.4 Hz, 1H), 7.57 (t, J = 7.2 Hz, 1H), 4.12 (s, 3H), 3.54 (bs, 4H), 3.29 (bs, 4H), 1.43 (s, 9H). ^{13}C NMR (101 MHz, DMSO-d_6) δ 158.2, 153.8, 145.3, 145.1, 119.9, 119.7, 79.4, 49.8, 43.8, 28.0. HRMS: $[M]^+$ calcd. for $C_{15}H_{24}N_3O_2^+$: 278.1863; found: 278.1866.

Compound **S8-I** (0.1 g, 0.24 mmol) was dissolved in 5 mL dichloromethane and 2 mL trifluoroacetic acid and stirred for 4 h at room temperature. Then, the solvent was removed in vacuo and the resulted residue was purified by column chromatography to get a light yellow solid (0.056 g, 76%). ^{1}H NMR (400 MHz, DMSO-d_6) δ 9.23 (bs, 1H), δ 8.71 (dd, J = 6.4, 1.6 Hz, 1H), 8.44-8.37 (m, 1H), 7.77 (d, J = 8.0 Hz, 1H), 7.67-7.60 (m, 1H), 4.14 (s, 3H), 3.56-3.49 (m, 4H), 3.36-3.35 (m, 4H). ^{13}C NMR (101 MHz, DMSO-d_6) δ 157.4, 145.6, 145.2, 120.4, 120.2, 47.1, 43.9, 42.3. HRMS: $[M]^+$ calcd. for $C_{10}H_{16}N_3^+$: 178.1339; found: 178.1341.

2. Spectra tests and HPLC analysis

General methods. 1-250 mM stock solutions of Na_2S in degassed (or by bubbling N_2 for 30 min) PBS buffer (pH 7.4) were used as H_2S source. The DMSO stock solutions for NBD amines were 1-20 mM, which were diluted in PBS buffer to afford the final concentration of 1-200 µM, respectively. All measurements were performed in a 3 mL sealed cuvette. The UV-visible spectra were recorded on a UV-3600 UV-VIS-NIR spectrophotometer. Fluorescence studies were carried out using a F-280 spectrophotometer or Varian Cary Eclipse spectrophotometer. All reaction mixtures were shaken uniformly before spectra measurements.

10 µM NBD-based reagents in PBS buffer (pH 7.4) were mixed with different concentrations of Na_2S in sealed cuvette, and the time-dependent absorbance spectra or intensity at 540 nm were recorded at 25 ºC. The pseudo-first-order rate, k_{obs} was determined by fitting the time-dependent intensity data with single exponential function. The plots of log(k_{obs}) vs log([H_2S]) were fitted linearly to indicate the reaction order for H_2S. The linear fitting between k_{obs} and [H_2S] gives the reaction rate (k_2).

The water solubility was tested by concentration-dependent absorbance spectra of the scavengers in PBS (50 mM, pH = 7.4).[4] In all cases, the final concentration of DMSO in buffer was maintained to be 2%. The plots of absorbance intensity at 470 nm for **NBD-S5** and at 490 nm for **NBD-S8** against the reagent concentrations were linearly fitted (Fig. S14 and S15). The maximum concentration in the linear region was taken as the water-solubility.

Fluorescence tests. 5 µM **NBD-S5** in PBS buffer was treated with different concentrations of H_2S (50, 60, 70, 80 µM), or 10 µM **NBD-S8** in PBS buffer was treated with different concentrations of H_2S (60, 80, 90, 100, 120 µM). Immediately, time-dependent fluorescence signals at 540 nm for **NBD-S5** or 550 nm for **NBD-S8** (both excitation at 470 nm) were recorded (Fig. S9 and S10). The data can be fitted by single exponential function, which can be used to calculate the reaction kinetics.

HPLC analysis. The reaction of **NBD-S8** (0.2 mM) and H_2S (0.5 mM) in PBS buffer was analyzed by HPLC at different reaction time (Fig. S12). The stability of NBD amines in buffer in the presence of biothiols was also tested by HPLC (Fig. S16 and S17).

Conditions: ANGELA TECHNOLOGIES HPLC LC-10F; C18 column with 4.6 mm x 250 mm; detection wavelength: 400 nm for **NBD-S5** and 254 nm for **NBD-S8**; flow 1.0 mL/min; buffer A: 0.1% (v/v) trifluoroacetic acid in water; buffer B: methanol; elution condition: 0-2 min, buffer B: 5%; 2-15 min, buffer B: 5-90%; 15-20, buffer B: 90-5%.

3. X-ray crystallography studies

Crystals of **NBD-S7** were obtained by recrystallization from dichloromethane and methanol (v:v, 1:1). The solution of **NBD-S7** was heated at 60 °C for 20 minutes. After filtration, the resulted clear solution was isothermally evaporated at room temperature. The red-transparent single crystals were emerged after several days.

Crystals of **NBD-S8** were obtained by recrystallization from dichloromethane-methanol (v:v, 1:2) with traces of triethylamine. The solution of **NBD-S8** was heated at 60 °C for 20 minutes. After filtration, the resulted clear solution was isothermally evaporated at room temperature. The red-transparent flat crystals were emerged within three days.

The single-crystal X-ray diffraction data set was collected on a Rigaku 007 Saturn 70 (Mo target) diffractometer. Used Olex2,[5] the structure was solved with the ShelXT structure solution program using Intrinsic Phasing and refined with the ShelXL refinement package using Least Squares minimization.[6] Details of the data collection and the structure refinements are summarized in Table S1. Crystallographic data (CIF) file for the structure has been deposited with the Cambridge Crystallographic Data Centre as supplementary (CCDC 1968528 for **NBD-S7**; 1973453 for **NBD-S8**). Copies of the data can be obtained, free of charge, on application to CCDC, 12 Union Road, Cambridge CB2 1EZ, United Kingdom, P: +44 (0)1223 336408, F: +44 (0)1223 336033 (e-mail: deposit@ccdc.cam.ac.uk).

Table S1 Crystal data and structure refinement for **NBD-S7**

Empirical formula	C15H16N6O3
Formula weight	326.32

Temperature	133.15 K
Wavelength	0.71073 Å
Crystal system	Monoclinic
Space group	P 1 21/c 1
Unit cell dimensions	a = 6.3096(6) Å α = 90°<br>b = 29.084(2) Å β = 91.450(9)°<br>c = 7.5539(8) Å γ = 90°
Volume	1385.7(2) $Å^3$
Z	4
Density (calculated)	1.564 Mg/m^3
Absorption coefficient	0.114 mm^{-1}
F (000)	680
Crystal size	0.32 x 0.14 x 0.12 mm^3
Crystal color and habit	Red
Diffractometer	Rigaku Oxford Diffraction
Theta range for data collection	5.574 to 52.728°
Index ranges	-7<=h<=7, -36<=k<=36, -9<=l<=9
Reflections collected	12221
Independent reflections	2826(R(sigma)= 0.0475)
Observed reflections (I > 2sigma(I))	2159
Completeness to theta = 32.80	100%
Absorption collection	spherical harmonics
Solution method	SHELXT 2014/5 (Sheldrick, 2014)
Refinement method	ShelXL 2018/3 (Sheldrick, 2015)
Data / restraints / parameters	2826/0/218
Goodness-of-fit on F^2	1.130
Final R indices (I>2sigma(I))	R1 = 0.0615, wR2 = 0.1544
R indices (all data)	R1 = 0.0816, wR2 = 0.1686
Largest diff. peak and hole	0.301 and -0.255 e.$Å^{-3}$

Table S2 Crystal data and structure refinement for **NBD-S8**

Empirical formula	C16H17N6O3I
Formula weight	468.25
Temperature	133.15 K
Wavelength	0.71073 Å
Crystal system	Monoclinic
Space group	P 1 21/c 1
Unit cell dimensions	a = 19.9828(7) Å α = 90°<br>b = 12.5986(5) Å β = 101.853(3)°<br>c = 14.2705(5) Å γ = 90°
Volume	3516.1(2) $Å^3$
Z	8
Density (calculated)	1.769 Mg/m^3
Absorption coefficient	1.852 mm^{-1}

F (000)	1856
Crystal size	0.16 x 0.14 x 0.04 mm^3
Crystal color and habit	Red
Diffractometer	Rigaku Oxford Diffraction
Theta range for data collection	3.846 to 52.742°
Index ranges	-24<=h<=24, -15<=k<=15, -14<=l<=17
Reflections collected	7186
Independent reflections	7186(R(sigma)=0.0336)
Observed reflections (I > 2sigma(I))	6571
Completeness to theta = 25.242	100%
Absorption collection	spherical harmonics
Solution method	SHELXS (Sheldrick, 2008)
Refinement method	SHELXL 2018/3 (Sheldrick, 2015)
Data / restraints / parameters	7186/6/472
Goodness-of-fit on F^2	1.198
Final R indices (I>2sigma(I))	R1 = 0.1022, wR2 = 0.2203
R indices (all data)	R1 = 0.1083, wR2 = 0.2243
Largest diff. peak and hole	1.937 and -2.912 e.Å^{-3}

Table S3 Atomic coordinates (x10^4) and equivalent isotropic displacement parameters (Å^{2}x10^4) for **NBD-S7**. U (eq) is defined as one third of the trace of the orthogonalized U^{ij} tensor.

	x	y	Z	U(eq)
C1	2420(5)	5609.1(9)	1541(4)	309(7)
O1	8287(3)	2962.0(6)	7132(3)	316(5)
N1	2891(4)	5192.0(8)	2252(3)	316(6)
C2	402(5)	5755.8(10)	1160(4)	333(7)
O2	6733(3)	1596.3(7)	6345(3)	373(5)
N2	1745(4)	4484.2(8)	3287(3)	282(5)
C3	1237(5)	5461.5(10)	1538(4)	351(7)
O3	3896(3)	1405.1(6)	4821(3)	354(5)
N3	2865(3)	3564.8(7)	4385(3)	227(5)
C4	845(4)	5040.6(9)	2305(4)	277(6)
N4	6828(4)	3258.8(8)	6398(3)	280(5)
C5	1251(4)	4909.5(8)	2618(4)	245(6)
N5	7771(4)	2502.4(8)	6815(3)	282(5)
C6	121(4)	4155.6(9)	3756(4)	260(6)
N6	5120(4)	1696.4(8)	5462(3)	280(5)
C7	844(4)	3664.0(8)	3449(4)	243(6)
C8	4116(4)	3966.8(9)	4952(4)	295(6)
C9	3935(4)	4352.2(9)	3610(4)	293(6)
C10	3453(4)	3127.9(9)	4653(3)	224(6)
C11	5381(4)	2994.6(9)	5615(3)	228(6)
C12	5969(4)	2520.9(9)	5883(3)	228(6)
C13	4638(4)	2169.2(9)	5193(3)	235(6)
C14	2814(4)	2299.9(9)	4299(3)	243(6)
C15	2223(4)	2755.5(9)	4024(4)	238(6)

Table S4 Atomic coordinates (x10^4) and equivalent isotropic displacement parameters (Å^{2}x10^4) for **NBD-S8**. U (eq) is defined as one third of the trace of the orthogonalized U^{ij} tensor.

	x	y	Z	U(eq)
C1	4329(9)	4579(14)	5471(12)	57(4)
O1	972(4)	6156(8)	8805(6)	35(2)
N1	3959(6)	5384(10)	5757(8)	42(3)
C2	4068(10)	3988(13)	4672(12)	56(4)
O2	-1108(5)	6017(8)	7768(6)	38(2)
N2	2939(6)	6283(11)	5683(7)	43(3)
C3	3428(9)	4243(14)	4137(12)	56(4)
O3	-1534(4)	6107(8)	6254(7)	40(2)
N3	1609(5)	6131(9)	6118(6)	33(2)
C4	3052(8)	5012(15)	4454(11)	56(4)
O4	597(4)	3550(7)	-3404(6)	34(2)
N4	1345(5)	6145(9)	8102(7)	33(2)
C5	3309(7)	5559(13)	5312(9)	43(3)
O5	-1384(5)	3712(8)	-2970(6)	41(2)
N5	279(6)	6146(9)	8444(7)	33(2)
C6	2859(6)	6232(15)	6676(9)	48(4)
O6	-1591(4)	3635(8)	-1540(7)	43(2)
N6	-1045(6)	6087(9)	6919(8)	35(2)
C7	2218(6)	5643(12)	6732(10)	41(3)
N7	4030(5)	4568(8)	908(7)	30(2)
C8	1687(6)	6258(13)	5111(8)	38(3)
N8	2865(5)	4222(9)	595(7)	30(2)
C9	2343(6)	6805(12)	5087(9)	39(3)
N9	1634(5)	3366(9)	-445(7)	31(2)
C10	993(6)	6097(11)	6319(9)	34(3)
N10	1087(5)	3490(9)	-2573(7)	32(2)
C11	884(7)	6105(10)	7294(9)	33(3)
N11	-71(5)	3614(9)	-3244(7)	33(2)
C12	220(6)	6107(9)	7509(8)	28(2)
N12	-1194(6)	3692(9)	-2104(7)	35(2)
C13	-370(6)	6121(9)	6751(8)	26(2)
C14	-263(6)	6109(10)	5825(8)	30(3)
C15	381(6)	6114(10)	5614(9)	31(3)
C16	4324(7)	6091(14)	6532(10)	47(4)
C17	4581(6)	5135(11)	1359(9)	37(3)
C18	4544(7)	5860(12)	2040(10)	40(3)
C19	3914(7)	6040(11)	2292(9)	41(3)
C20	3342(7)	5517(11)	1815(9)	36(3)
C21	3399(6)	4796(11)	1105(8)	31(3)
C22	2275(6)	1063(8)	4030(12)	36(3)
C23	1877(6)	3133(11)	568(8)	32(3)
C24	2221(6)	3541(12)	-911(8)	37(3)
C25	2627(6)	4447(11)	-438(8)	33(3)
C26	980(6)	3535(10)	-841(7)	26(2)
C27	724(6)	3577(10)	-1884(8)	29(3)
C28	17(7)	3628(10)	-2305(8)	32(3)
C29	-484(6)	3685(10)	-1717(8)	30(3)
C30	-224(6)	3710(10)	-736(8)	30(3)
C31	458(6)	3664(10)	-312(8)	29(3)
C32	4123(7)	3723(12)	271(10)	45(3)

Table S5 Bond lengths [Å] and angle [º] for **NBD-S7**.

O1 N4	1.369(3)	O3 N6 C13	118.5(2)
O1 N5	1.395(3)	O2 N6 C13	118.3(2)
O3 N6	1.237(3)	C11 N4 O1	105.3(2)
O2 N6	1.237(3)	C12 N5 O1	104.2(2)
N3 C10	1.338(3)	C5 N2 C6	122.0(2)
N3 C7	1.471(3)	C5 N2 C9	120.6(2)
N3 C8	1.468(3)	C9 N2 C6	117.4(2)
N6 C13	1.422(3)	C5 N1 C1	117.5(2)
N4 C11	1.322(3)	N2 C5 C4	121.1(2)
N5 C12	1.323(3)	N1 C5 N2	117.1(2)
N2 C5	1.369(3)	N1 C5 C4	121.8(2)
N2 C6	1.452(3)	N3 C10 C11	123.7(2)
N2 C9	1.449(3)	N3 C10 C15	122.1(2)
N1 C5	1.355(3)	C15 C10 C11	114.3(2)
N1 C1	1.356(3)	N4 C11 C10	129.0(2)
C5 C4	1.390(4)	N4 C11 C12	108.7(2)
C10 C11	1.454(4)	C12 C11 C10	122.3(2)
C10 C15	1.408(4)	N5 C12 C11	109.2(2)
C11 C12	1.440(4)	N5 C12 C13	131.4(2)
C12 C13	1.415(4)	C13 C12 C11	119.4(2)
C13 C14	1.373(4)	C12 C13 N6	121.6(2)
C14 C15	1.391(4)	C14 C13 N6	120.8(2)
C7 C6	1.520(3)	C14 C13 C12	117.6(2)
C8 C9	1.514(4)	C1 C2 C3	117.6(3)
C4 C3	1.374(4)	N1 C1 C2	123.8(3)
C2 C1	1.367(4)	C13 C14 C15	123.8(2)
C2 C3	1.378(4)	C14 C15 C10	122.6(2)
		N3 C7 C6	111.8(2)
N4 O1 N5	112.54(19)	N2 C6 C7	111.4(2)
C10 N3 C7	119.5(2)	N3 C8 C9	111.3(2)
C10 N3 C8	124.6(2)	C3 C4 C5	118.4(3)
C8 N3 C7	115.9(2)	N2 C9 C8	111.4(2)
O3 N6 O2	123.1(2)	C4 C3 C2	120.8(3)

Table S6 Bond lengths [Å] and angle [º] for **NBD-S8**.

O1 N4	1.368(12)	O2 N6 C13	117.1(10)
O1 N5	1.373(13)	O3 N6 O2	122.3(11)
O2 N6	1.247(13)	O3 N6 C13	120.5(10)
O3 N6	1.215(13)	N1 C1 C2	120.9(16)
N1 C1	1.37(2)	C1 C2 C3	118.9(16)
N1 C5	1.341(17)	C4 C3 C2	119.5(16)
N1 C16	1.489(18)	C3 C4 C5	120.7(16)
N2 C5	1.349(19)	N1 C5 N2	118.7(13)
N2 C6	1.459(15)	N1 C5 C4	118.4(14)
N2 C9	1.469(17)	N2 C5 C4	122.8(14)
N3 C7	1.480(16)	N2 C6 C7	109.8(11)
N3 C8	1.484(13)	N3 C7 C6	111.7(11)
N3 C10	1.321(16)	N3 C8 C9	109.9(10)
N4 C11	1.321(16)	N2 C9 C8	113.1(11)
N5 C12	1.317(15)	N3 C10 C11	122.6(11)
N6 C13	1.418(15)	N3 C10 C15	123.6(11)

C1 C2	1.37(2)	C15 C10 C11	113.7(11)
C2 C3	1.39(2)	N4 C11 C10	128.4(12)
C3 C4	1.36(2)	N4 C11 C12	109.1(10)
C4 C5	1.41(2)	C12 C11 C10	122.4(11)
C6 C7	1.496(18)	N5 C12 C11	108.9(11)
C8 C9	1.488(17)	N5 C12 C13	131.0(11)
C10 C11	1.452(16)	C11 C12 C13	120.0(11)
C10 C15	1.416(17)	C12 C13 N6	122.6(10)
C11 C12	1.422(17)	C14 C13 N6	120.0(11)
C12 C13	1.426(16)	C14 C13 C12	117.3(11)
C13 C14	1.382(16)	C15 C14 C13	122.9(11)
C14 C15	1.379(17)	C14 C15 C10	123.6(11)
O4 N10	1.377(13)	N10 O4 N11	113.3(8)
O4 N11	1.401(13)	C17 N7 C21	118.7(11)
O5 N12	1.217(13)	C17 N7 C32	119.5(11)
O6 N12	1.241(13)	C21 N7 C32	121.8(11)
N7 C17	1.358(16)	C21 N8 C22	116.8(9)
N7 C21	1.378(14)	C21 N8 C25	118.9(10)
N7 C32	1.437(17)	C25 N8 C22	110.8(9)
N8 C21	1.368(15)	C23 N9 C24	110.1(9)
N8 C22	1.490(14)	C26 N9 C23	123.4(9)
N8 C25	1.481(14)	C26 N9 C24	125.9(10)
N9 C23	1.458(14)	C27 N10 O4	103.3(9)
N9 C24	1.479(14)	C28 N11 O4	103.5(10)
N9 C26	1.330(15)	O5 N12 O6	123.6(11)
N10 C27	1.342(15)	O5 N12 C29	118.3(11)
N11 C28	1.316(15)	O6 N12 C29	118.0(10)
N12 C29	1.414(16)	C18 C17 N7	122.6(12)
C17 C18	1.35(2)	C17 C18 C19	118.9(13)
C18 C19	1.40(2)	C20 C19 C18	119.8(13)
C19 C20	1.374(18)	C19 C20 C21	119.5(12)
C20 C21	1.383(17)	N7 C21 C20	120.2(11)
C22 C23	1.475(18)	N8 C21 N7	115.3(11)
C24 C25	1.480(18)	N8 C21 C20	124.4(11)
C26 C27	1.471(15)	C23 C22 N8	108.0(10)
C26 C31	1.417(14)	N9 C23 C22	110.9(10)
C27 C28	1.417(17)	N9 C24 C25	108.7(10)
C28 C29	1.434(16)	C24 C25 N8	109.9(10)
C29 C30	1.390(16)	N9 C26 C27	122.9(10)
C30 C31	1.374(17)	N9 C26 C31	123.9(10)
N4 O1 N5	112.6(8)	C31 C26 C27	113.2(10)
C1 N1 C16	116.9(13)	N10 C27 C26	127.6(11)
C5 N1 C1	120.8(14)	N10 C27 C28	109.6(10)
C5 N1 C16	122.1(13)	C28 C27 C26	122.7(10)
C5 N2 C6	122.0(12)	N11 C28 C27	110.1(11)
C5 N2 C9	121.3(11)	N11 C28 C29	129.3(12)
C6 N2 C9	110.2(11)	C27 C28 C29	120.6(11)
C7 N3 C8	112.4(10)	N12 C29 C28	122.5(11)
C10 N3 C7	123.2(10)	C30 C29 N12	122.1(11)
C10 N3 C8	120.1(10)	C30 C29 C28	115.4(11)
C11 N4 O1	104.6(10)	C31 C30 C29	125.1(11)
C12 N5 O1	104.7(9)	C30 C31 C26	122.8(10)

4. H_2S scavenging tests

Normally, H_2S concentration was determined via the Methylene Blue Assay (MBA).[7] The stock solutions of 1% $Zn(OAc)_2$ in water, 30 mM $FeCl_3$ in 1.2 M HCl, 20 mM *N*,*N*-dimethyl-*p*-phenylene diamine in 7.2 M HCl were prepared. The methylene blue cocktail solution was freshly prepared by mixture of $Zn(OAc)_2$, $FeCl_3$, *N*,*N*-dimethyl-*p*-phenylene diamine solutions in 1:2:2 (v:v:v). A solution (1.8 mL) containing 0.9 mL of the methylene blue cocktail and 0.9 mL degassed PBS in cuvettes was used as MBA background solutions. A 250 mM stock solution of Na_2S in degassed PBS was prepared on ice and diluted to 1-10 mM. Immediately after dilution, Na_2S was added to the 1.8 mL MBA solution for final concentrations of 10, 20, 30, 40, and 50 μM, respectively. Solutions were mixed thoroughly, and incubated at 25 °C for 1 h. Absorbance values at 670 nm were measured for the MBA calibration curve.

The 20 mM stock solutions of scavengers **NBD-S2**, **NBD-S5** and **NBD-S8** were prepared in DMSO. Na_2S (0.1 mM) in 40 mL degassed PBS under argon gas protection was treated with or without the H_2S scavenger (0.11 mM) in a sealed flask. At a certain time-point, 0.9 mL Na_2S solution was drawn off and mixed with 0.9 mL of the methylene blue cocktail. After incubation, the resulted solution was checked by absorbance at 670 nm to determine the H_2S concentration. Different biologically relevant molecules (100 mM) were prepared as stock solutions in degassed PBS buffer or DMSO. H_2S scavenging in the presence of other species was performed as description above. Each species Arg (1 mM), Cys (0.5 mM), GSH (1 mM), Tyr (1 mM), Trp (1 mM), H_2O_2 (0.1 mM), or Na_2SO_3 (0.1 mM), respectively, was mixed with H_2S (0.1 mM) in degassed buffer (pH 7.4), and then **NBD-S8** (0.11 mM) was added to start the scavenging. The H_2S-scavenging in 10% fetal bovine serum (FBS) and 90% PBS buffer (pH = 7.4) was performed using Na_2S (0.1 mM) with **NBD-S8** (0.11 mM) in a sealed flask under argon gas protection. The time-dependent H_2S concentrations were determined as description above. Each test was performed in triplicate unless stated otherwise.

H_2S scavenging in gas:[8] the device was demonstrated in Fig. S22 by centrifuge tube (A) and glass tubes (B and C). Tube A: Na_2S solution (9 mg in 2 mL H_2O); tube B: **NBD-S8** solution (0.2 mM, 20 mL) in PBS buffer (50 mM, pH = 7.4); tube C: $AgNO_3$ solution

(0.1 M, 30 mL). Continuous nitrogen gas was purged into tube A. Then HCl (2 M, 5 mL) in 5 mL syringe was injected into tube A to release H_2S into the N_2 gas flow. After 20 min, 0.9 mL **NBD-S8** solution was mixed with 0.9 mL of the methylene blue cocktail and incubated for 1 h to determine the H_2S concentration as 0 μM.

5. Cell culture, MTT assay and bioimaging

The HeLa cell, HT-29 and FHC cell lines were purchased from the Cell Bank of the Chinese Academy of Sciences (Shanghai, China). HeLa cells were cultured in high glucose DMEM medium, while HT-29 and FHC cells were cultured in RPMI 1640 medium with 10% fetal bovine serum and 1% penicillin/streptomycin under standard cell culture conditions at 37 °C in a humidified incubator with 5% CO_2.

HeLa cells were used for evaluating the cytotoxicity because nearly no endogenous H_2S exists in HeLa cells, which may avoid the potential H_2S-scavenging effects. The cytotoxicity of **NBD-S8** or **S8-II** was determined via a 3-(4,5-dimethylthiazol-2-yl)-2,5-diphenyltetrazolium bromide (MTT) assay by using the HeLa cells. Briefly, cells were transferred to the 96-well plate and cultured for one night before experiments. After that, the culture medium was replaced with a fresh one and the cells were incubated with **NBD-S8** (0-200 μM) or **S8-II** (0-100 μM) for 24 h. Then, 5 mg/mL MTT in sterile water (20 μL) was added to each well and incubated for another 4 h. Finally, the medium was replaced with 150 μL of DMSO to dissolve the purple formazan crystals. The absorbance intensity in each well was detected at 490 nm by a microplate spectrophotometer (SpectraMax M2E (Molecular Device, Inc.)).

NBD-SH was prepared by a previous literature procedure.[9] HeLa cells were plated at a density of 100 k cells/well on a plastic 96 well plate in 10% FBS and 1% penicillin in phenol red-containing DMEM. The next day, the media was aspirated and cells were rinsed with FBS and phenol red-free DMEM. Cells then treated with different concentrations of NBD-SH (0-100 μM) in FBS and phenol red-free DMEM for 24 hours. The media was removed, and the cells were rinsed again. The media was replaced with

FBS and phenol red-free DMEM containing 10% CCK8 reagent (Dojindo) and further incubated until the assay was complete. Measurements were recorded using a BioTek Synergy 2 plate reader at 450 nm.

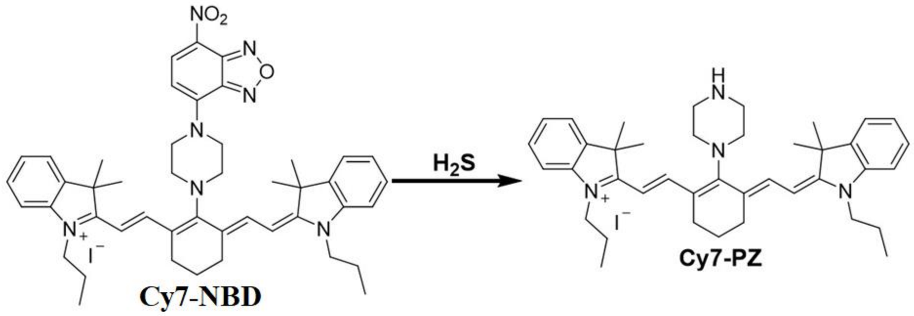

All bioimaging were based on our previous probe **Cy7-NBD**,[10] which could be used to vasualize H_2S in living cells and mice.

Scavenging H_2S in living cells by **NBD-S8** was visualized by cell imaging via the probe **Cy7-NBD**. FHC and HT-29 cells were used for evaluating the feasibility of **NBD-S8** for endogenous H_2S scavenging in cells, since high endogenous H_2S exists in HT-29 cells. While HeLa cells were used for evaluating the feasibility of **NBD-S8** for scavenging the enzymatic-produced H_2S, since nearly no endogenous H_2S exists in HeLa cells.

Briefly, glass bottom dishes were added into a 24-well plate for cell imaging before cells were seeded. Then, the cells were transferred to the 24-well plate and cultured for one night before the experiments. After that, the culture medium was replaced with the fresh one and the cells were treated with the desired reagents. Normally, HeLa cells were preincubated with D-Cys (100 μM or 200 μM) in the presence or absence of **NBD-S8** (100 μM) for 30 min, and then incubated with **Cy7-NBD** (10 μM) for 30 min. While FHC and HT-29 cells were incubated with the probe **Cy7-NBD** (10 μM) for 30 min to visualize the level of endogenous H_2S. And another group of HT-29 cells were pre-incubated with **NBD-S8** (100 μM) for 10 min, and then treated with the probe **Cy7-NBD** (10 μM) for 30 min. After incubation, the cells were quickly washed with PBS three times, and then fixed with 4% paraformaldehyde solution for 10 min. Finally, the cells were washed with PBS and stained with DAPI (5 μg/mL) for 10 min, and then imaged via a confocal microscope (Olympus FV1000) with a 40×objective lens. Emission was collected at the blue channel (450-550 nm, excitation at 405 nm) and the red channel

(650-750 nm, excitation at 647 nm). All images were analyzed with Olympus FV1000-ASW.

6. Optical *in vivo* imaging

BALB/c nude mice (4 weeks old) were purchased from the Chinese Academy of Military Medical Sciences (Beijing, China). All animal procedures were performed in accordance with the Guidelines for Care and Use of Laboratory Animals of Tianjin Medical University and approved by the Animal Ethics Committee of Tianjin Medical University. To evaluate the feasibility of **NBD-S8** for H_2S scavenging *in vivo*, exogenous H_2S was first produced via injecting mice model with Na_2S, and then the H_2S levels in mice body treated by **NBD-S8** or not were visualized by the probe **Cy7-NBD**. Briefly, mice were intraperitoneally (i.p.) injected with the probe **Cy7-NBD** (150 µM, 200 µL) only, with Na_2S (100 µM, 200 µL) and then **Cy7-NBD** (150 µM, 200 µL), with the Na_2S (100 µM, 200 µL) followed by **NBD-S8** (100 µM, 300 µL) for 10 min and then **Cy7-NBD** (150 µM, 200 µL). Fluorescent images were acquired at 10 min post injection.

To verify the feasibility of **NBD-S8** for scavenging endogenous H_2S in mice models, 8 mice were divided into two groups: one group was treated with **Cy7-NBD** only (150 µM, 200 µL) via tail vein injection as control; the second group was pretreated with **NBD-S8** (100 µM, 200 µL) for 10 min, and then with the probe **Cy7-NBD** (150 µM, 200 µL). Fluorescent images were acquired at 10, 20, 25 and 30 min post injection.

An IVIS spectrum imaging system (PerkinElmer, Massachusetts, USA) was used to detect the fluorescence of the probe **Cy7-NBD** (Ex./Em. = 740/800 nm). During the imaging, the mice were anesthetized with 2.5% isoflurane gas in an oxygen flow (1.5 L/min). Images were analyzed using Living Image 4.3.1 software (Xenogen).

7. Supporting figures

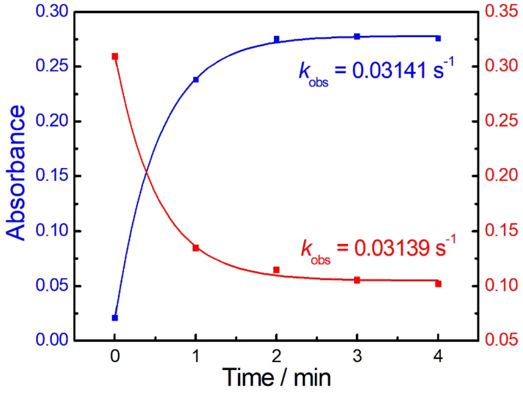

Fig. S1. Time-dependent absorbance signals at 540 nm (increase blue plots) or at 490 nm (decrease red plots) of 10 μM **NBD-S2** towards 1 mM H_2S in PBS buffer (50 mM, pH = 7.4, containing 2% DMSO) at 25 °C. The solid lines represent the best pseudo-first-order fitting with single exponential function.

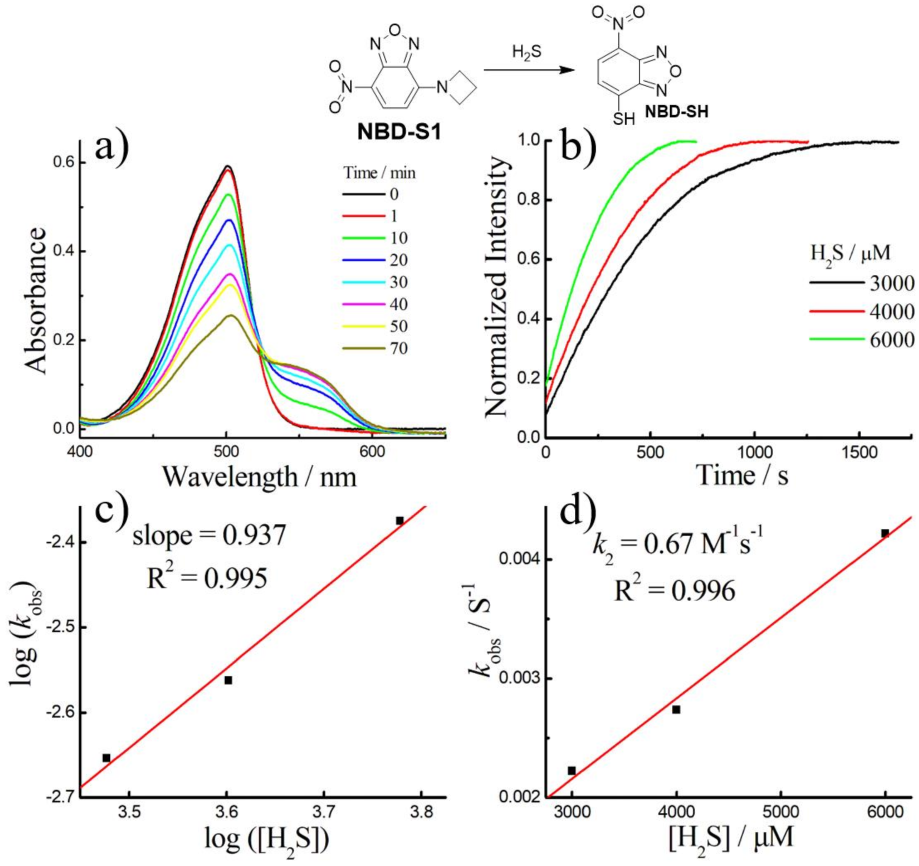

Fig. S2. (a) Time-dependent absorbance spectra of 10 μM **NBD-S1** towards 1 mM H_2S in PBS buffer (50 mM, pH = 7.4, containing 2% DMSO) at 25 °C. (b) Time-dependent normalized absorbance signals at 540 nm of 10 μM **NBD-S1** towards different concentrations of H_2S. The pseudo-first-order rate, k_{obs}, was determined by fitting the intensity data with single exponential function. (c) The reaction order of H_2S was determined as the slope of plots of log(k_{obs}) versus log([H_2S]) for **NBD-S1**. (d) The reaction rate k_2 (0.67 $M^{-1}s^{-1}$) was determined as the slope of plots of k_{obs} versus [H_2S].

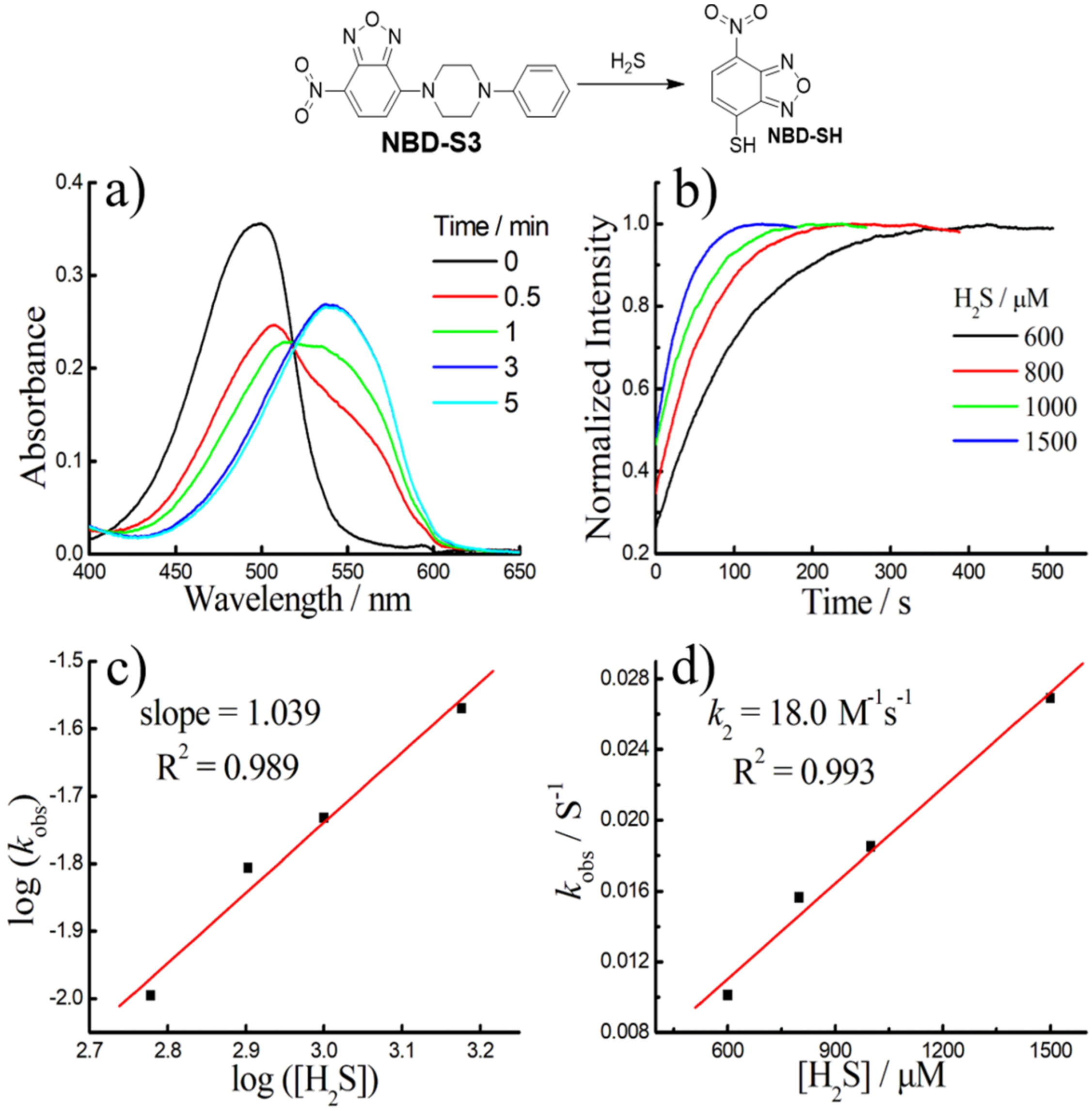

Fig. S3. (a) Time-dependent absorbance spectra of 10 μM **NBD-S3** towards 1 mM H_2S in PBS buffer (50 mM, pH = 7.4, containing 30% DMSO, due to low solubility of the compound) at 25 °C. (b) Time-dependent normalized absorbance signals at 540 nm of 10 μM **NBD-S3** towards different concentrations of H_2S (inset). The k_{obs} was determined by fitting the intensity data with single exponential function. (c) The reaction order of H_2S was determined as the slope of plots of log(k_{obs}) versus log([H_2S]) for **NBD-S3**. (d) The reaction rate k_2 (18.0 $M^{-1}s^{-1}$) was determined as the slope of plots of k_{obs} versus [H_2S].

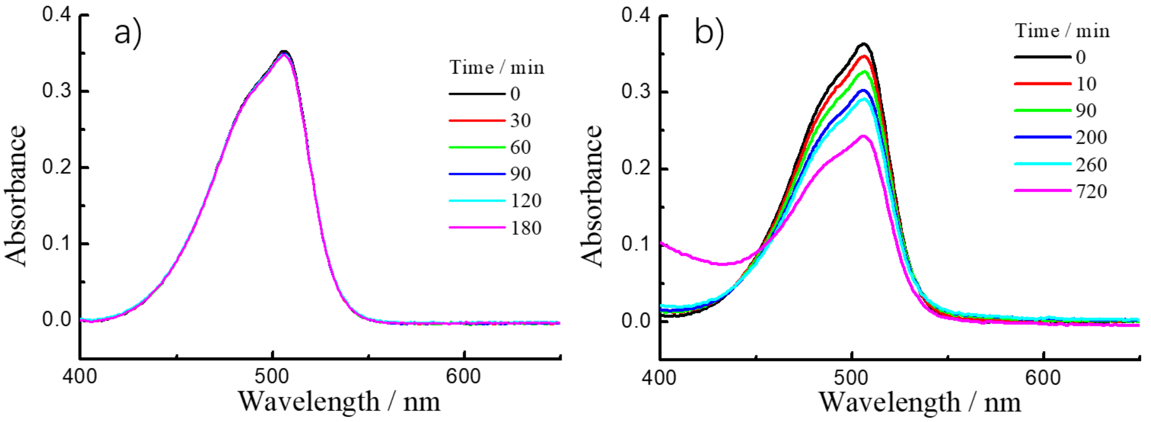

Fig. S4. (a) Time-dependent absorbance spectra of 10 μM **NBD-S4** in PBS buffer (50 mM, pH = 7.4, containing 2% DMSO) at 25 °C. (b) Time-dependent absorbance spectra of 10 μM **NBD-S4** towards 4 mM H_2S in PBS buffer at 25 °C.

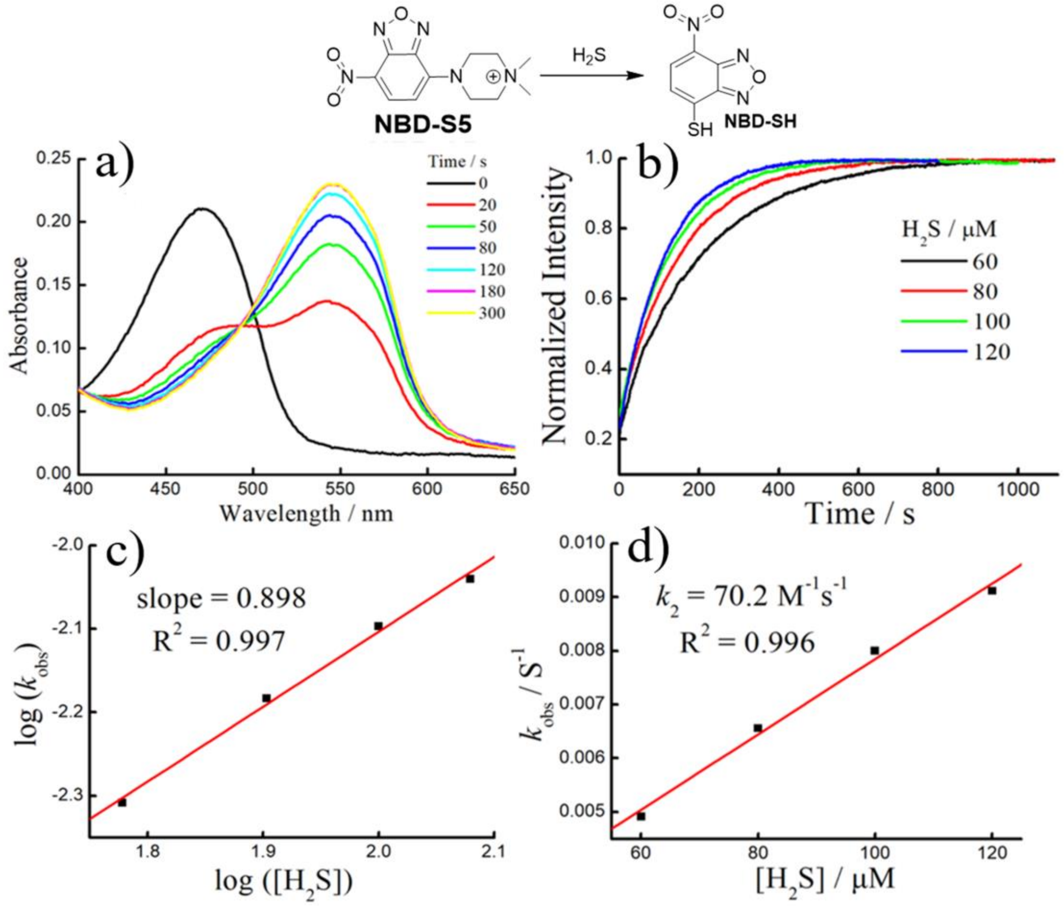

Fig. S5. (a) Time-dependent absorbance spectra of 10 μM **NBD-S5** towards 500 μM H_2S in PBS buffer (50 mM, pH = 7.4, containing 2% DMSO) at 25 °C. (b) Time-dependent normalized absorbance signals at 540 nm of 10 μM **NBD-S5** towards different concentrations of H_2S (inset). The k_{obs} was determined by fitting the intensity data with single exponential function. (c) The reaction order of H_2S was determined as the slope of plots of log(k_{obs}) versus log([H_2S]) for **NBD-S5**. (d) The reaction rate k_2 (70.2 $M^{-1}s^{-1}$) was determined as the slope of plots of k_{obs} versus [H_2S].

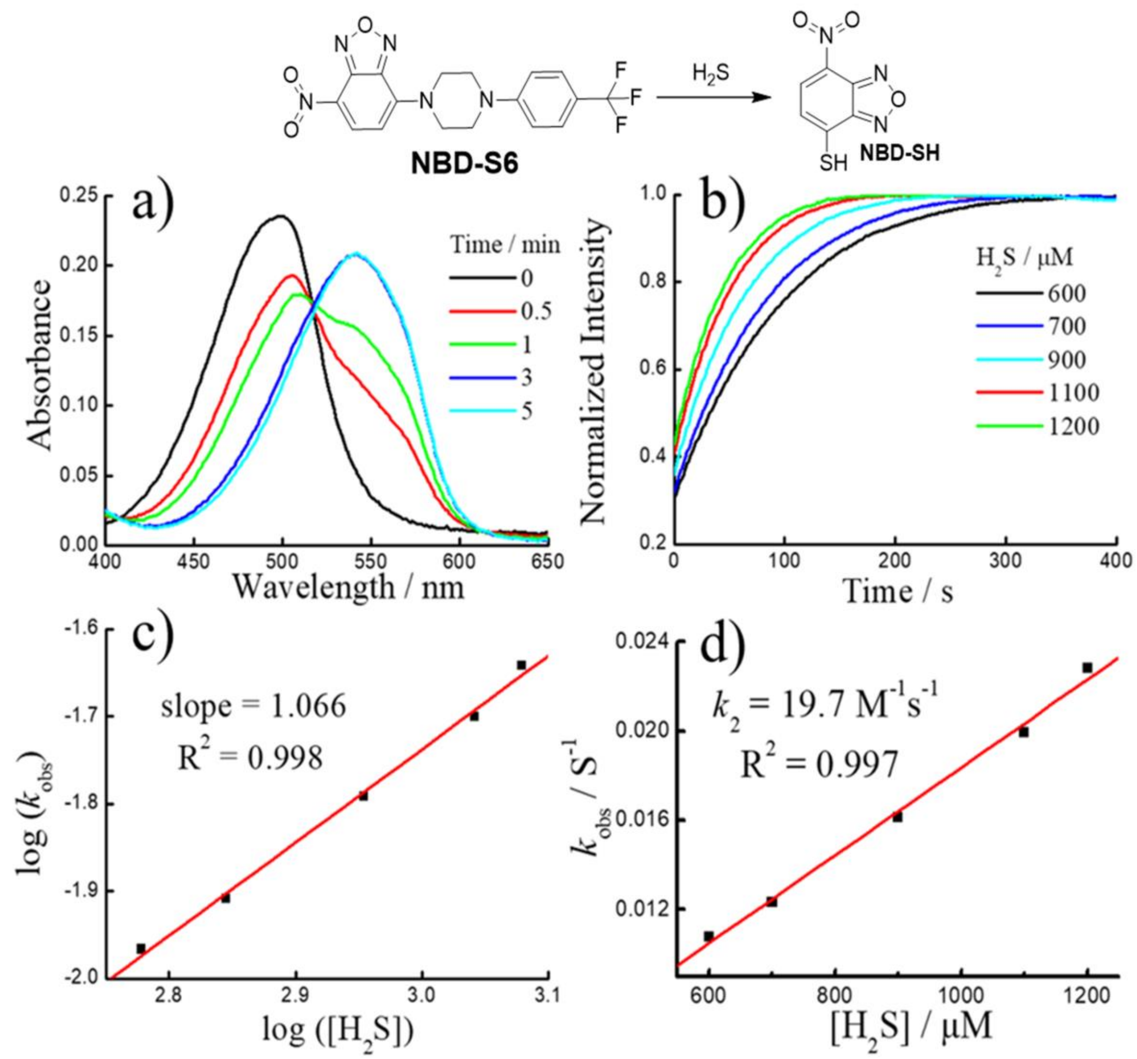

Fig. S6. (a) Time-dependent absorbance spectra of 10 μM **NBD-S6** towards 1 mM H_2S in PBS buffer (50 mM, pH = 7.4, containing 30% DMSO) at 25 °C. (b) Time-dependent normalized absorbance signals at 540 nm of 10 μM **NBD-S6** towards different concentrations of H_2S (inset). The k_{obs} was determined by fitting the intensity data with single exponential function. (c) The reaction order of H_2S was determined as the slope of plots of log(k_{obs}) versus log([H_2S]) for **NBD-S6**. (d) The reaction rate k_2 (19.7 $M^{-1}s^{-1}$) was determined as the slope of plots of k_{obs} versus [H_2S].

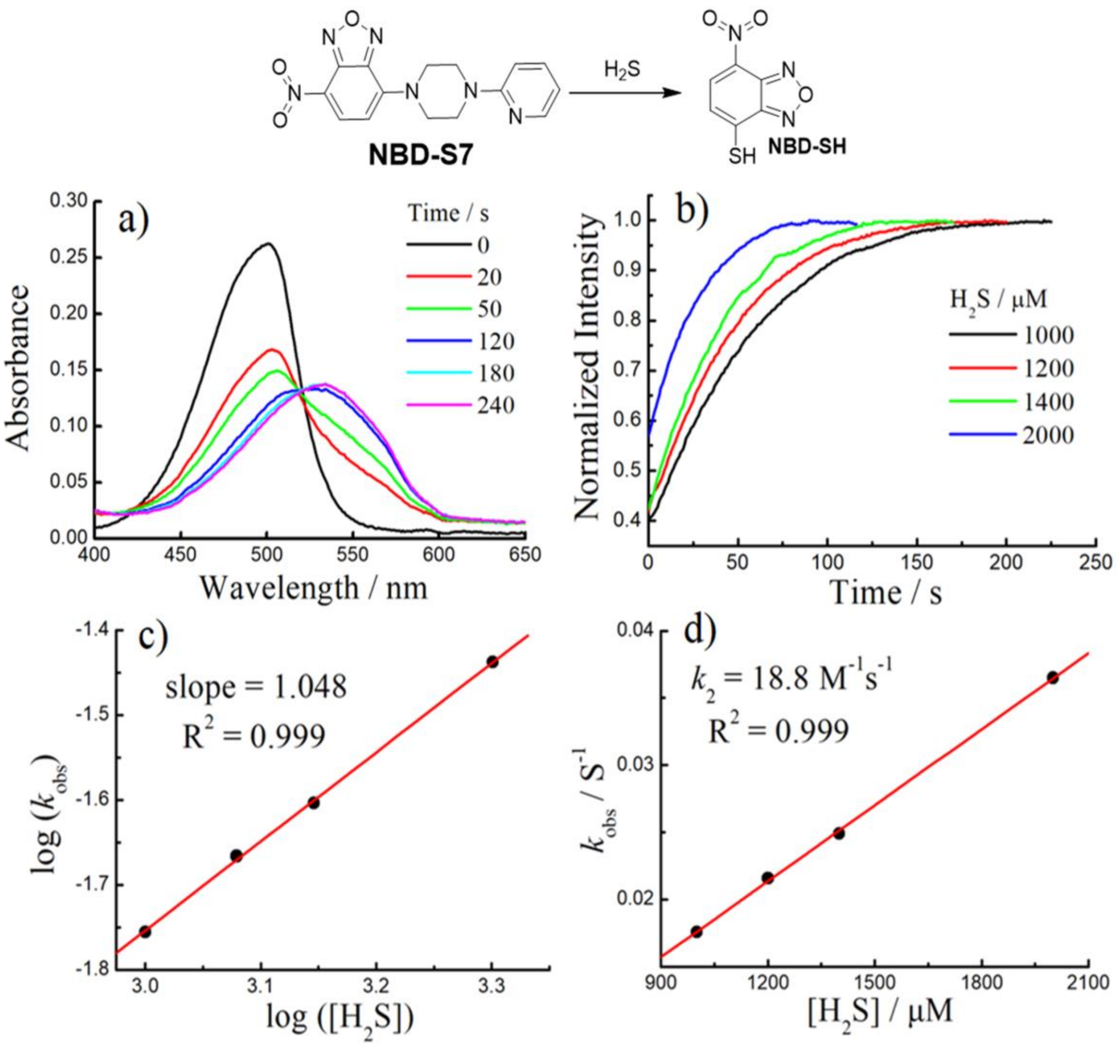

Fig. S7. (a) Time-dependent absorbance spectra of 10 μM **NBD-S7** towards 1 mM H_2S in PBS buffer (50 mM, pH = 7.4, containing 2% DMSO) at 25 ℃. (b) Time-dependent normalized absorbance signals at 540 nm of 10 μM **NBD-S7** towards different concentrations of H_2S (inset). The k_{obs} was determined by fitting the intensity data with single exponential function. (c) The reaction order of H_2S was determined as the slope of plots of log(k_{obs}) versus log([H_2S]) for **NBD-S7**. (d) The reaction rate k_2 (18.8 $M^{-1}s^{-1}$) was determined as the slope of plots of k_{obs} versus [H_2S].

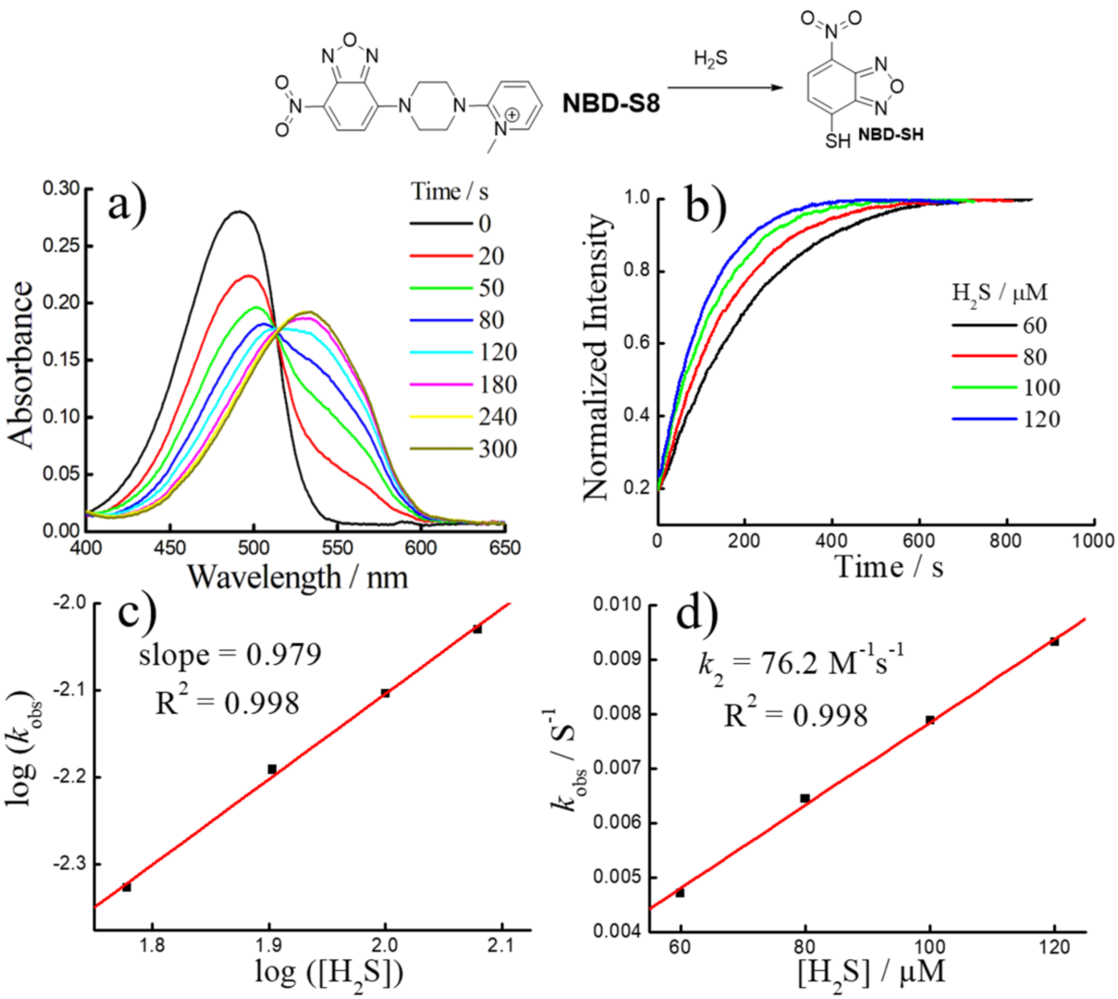

Fig. S8. (a) Time-dependent absorbance spectra of 10 μM **NBD-S8** towards 250 μM H_2S in PBS buffer (50 mM, pH = 7.4, containing 2% DMSO) at 25 ℃. (b) Time-dependent normalized absorbance signals at 530 nm of 10 μM **NBD-S8** towards different concentrations of H_2S (inset). The k_{obs} was determined by fitting the intensity data with single exponential function. (c) The reaction order of H_2S was determined as the slope of plots of log(k_{obs}) versus log([H_2S]) for **NBD-S8**. (d) The reaction rate k_2 (76.2 $M^{-1}s^{-1}$) was determined as the slope of plots of k_{obs} versus [H_2S].

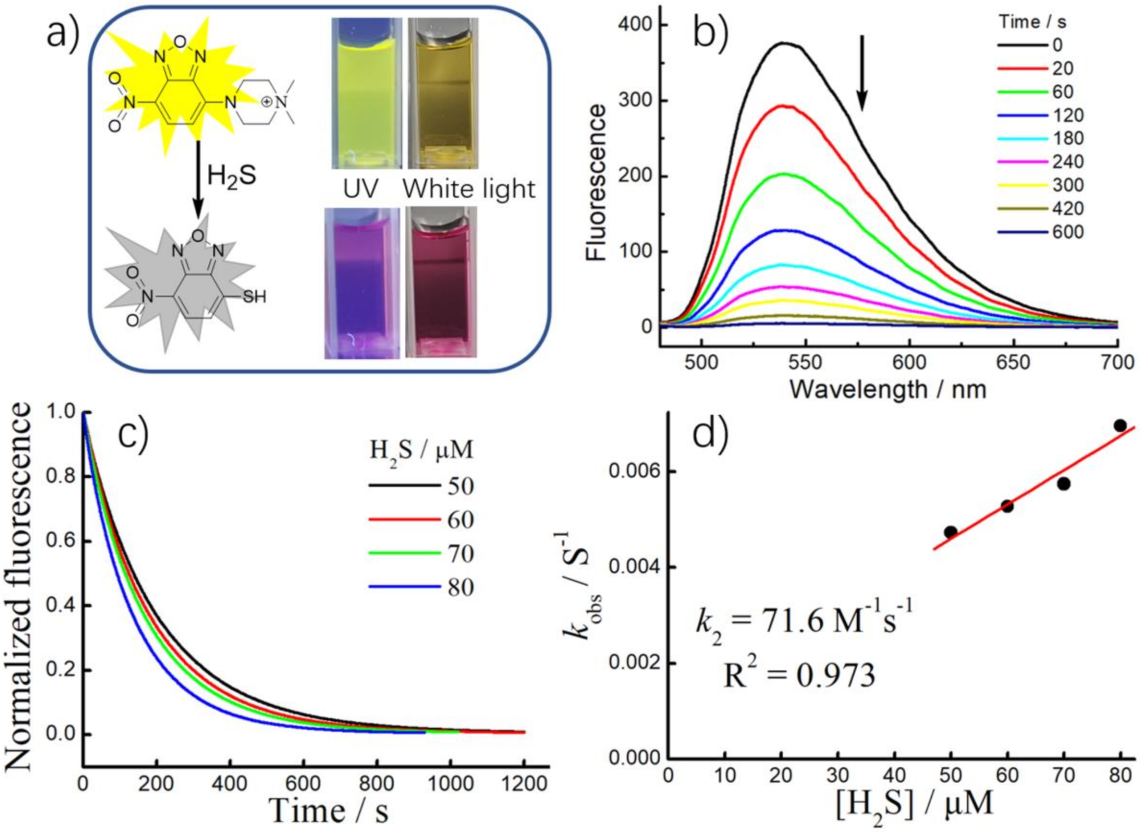

Fig. S9. (a) Fluorogenic quenching of **NBD-S5** by H_2S can be visualized under 365 nm UV lamp. (b) Time-dependent fluorescence spectra of 5 µM **NBD-S5** in the presence of 100 µM H_2S at 25 °C. (c) Time-dependent normalized fluorescence intensities at 540 nm of 5 µM **NBD-S5** in the presence of different concentrations of H_2S. (d) Plots of k_{obs} vs. H_2S concentrations give the reaction rate k_2.

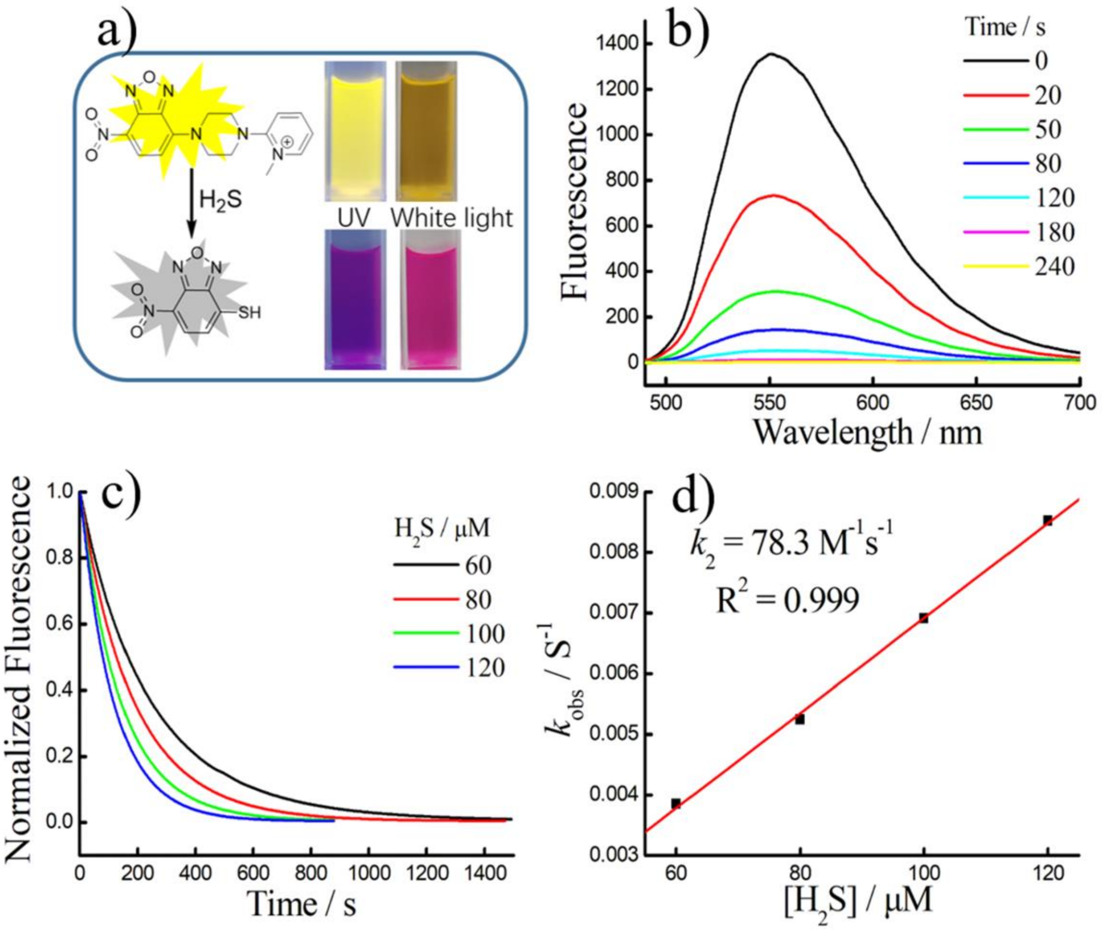

Fig. S10. (a) Fluorogenic quenching of **NBD-S8** by H_2S can be visualized under 365 nm UV lamp. (b) Time-dependent fluorescence spectra of 10 μM **NBD-S8** in the presence of 250 μM H_2S at 25 °C. (c) Time-dependent normalized fluorescence intensities at 550 nm of 10 μM **NBD-S8** in the presence of different concentrations of H_2S. (d) Plots of k_{obs} vs. H_2S concentrations give the reaction rate k_2.

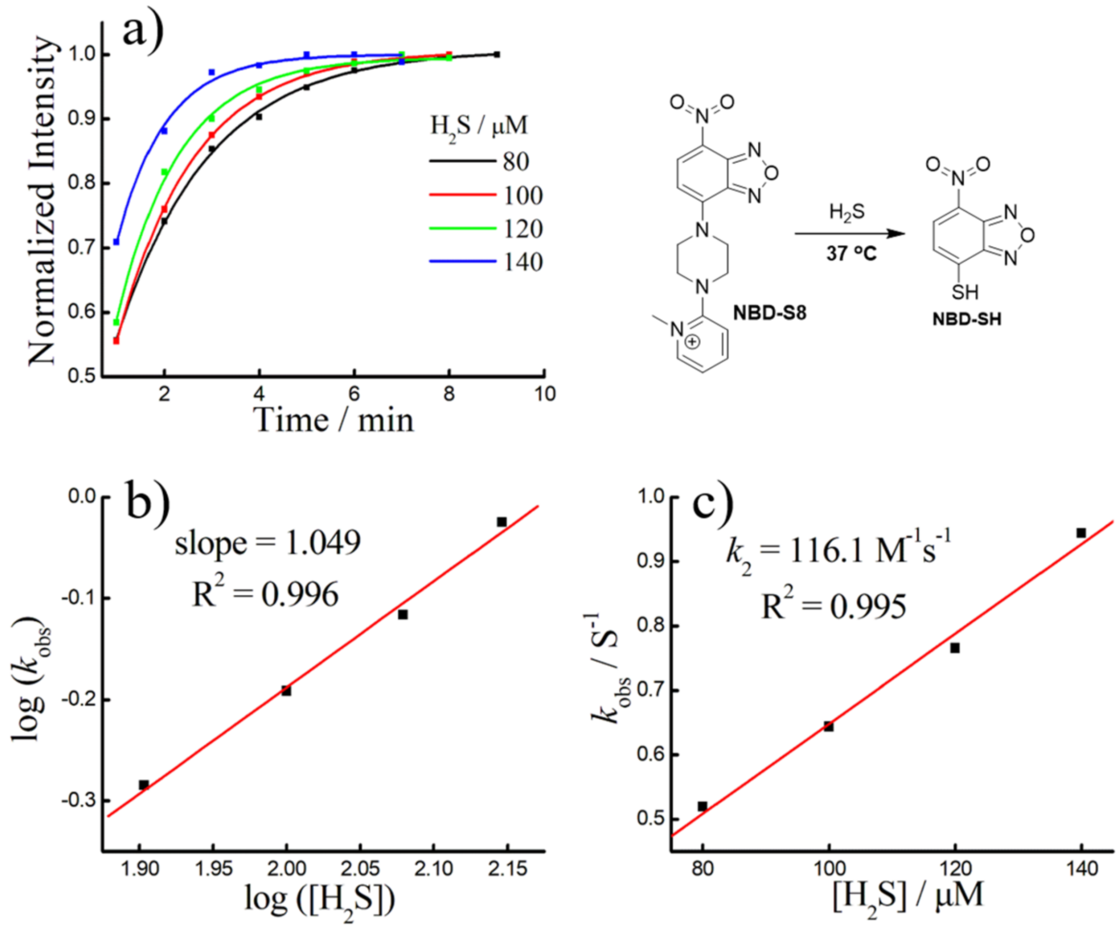

Fig. S11. (a) Time-dependent absorbance spectra at 530 nm of 10 µM **NBD-S8** towards different concentrations of H_2S (inset) in PBS buffer (50 mM, pH = 7.4, containing 2% DMSO) at 37 °C. The k_{obs} was determined by fitting the intensity data with single exponential function. (c) The reaction order of H_2S was determined as the slope of plots of log(k_{obs}) versus log([H_2S]) for **NBD-S8**. (d) The reaction rate k_2 (116.1 $M^{-1}s^{-1}$) was determined as the slope of plots of k_{obs} versus [H_2S].

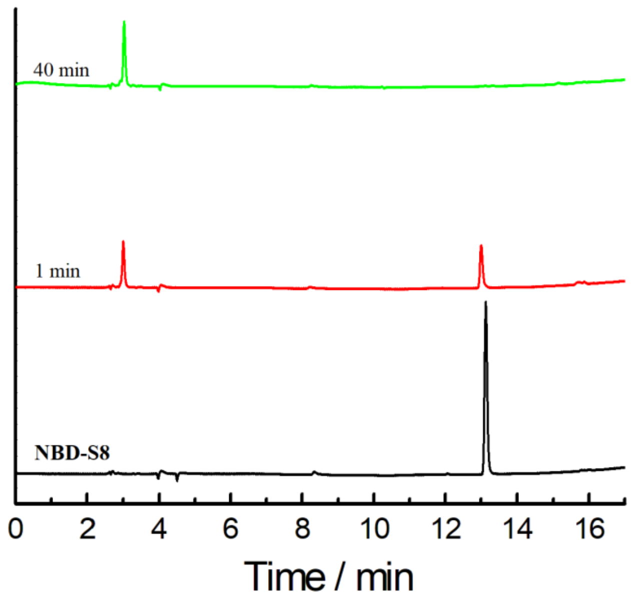

Fig. S12. Time-dependent HPLC traces of the reaction of **NBD-S8** (0.2 mM) with H_2S (0.5 mM) in PBS buffer (50 mM, pH 7.4).

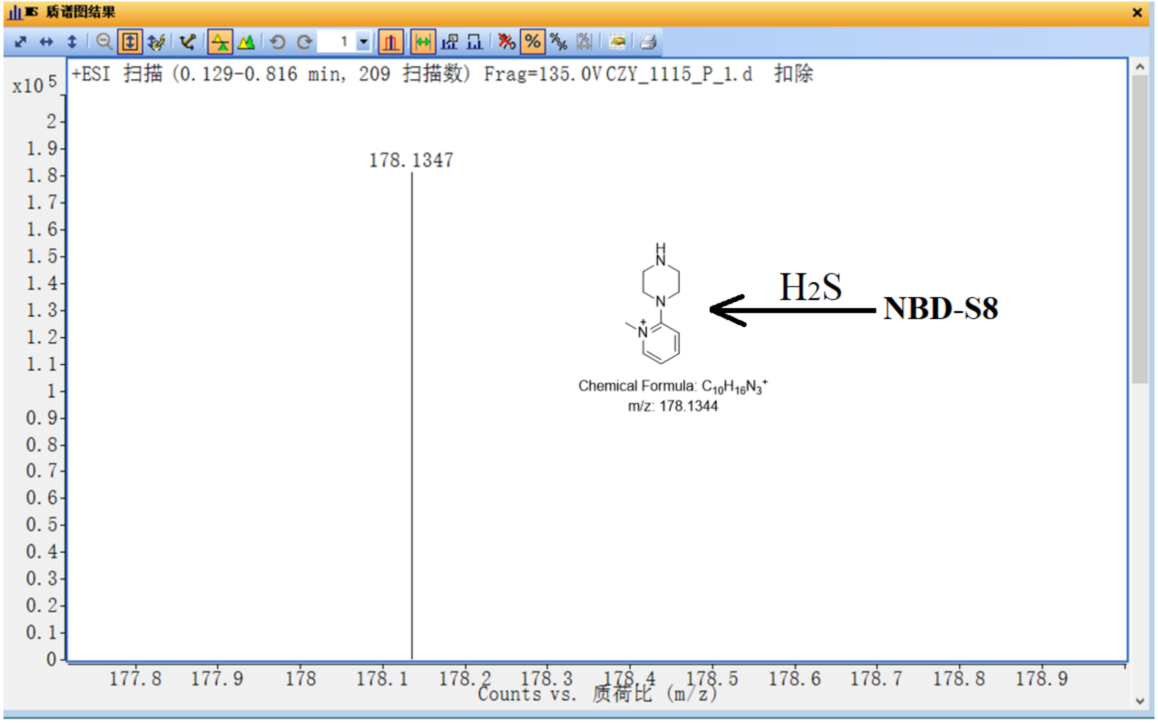

Fig. S13. HRMS of the reaction solution of **NBD-S8** with H_2S to give the expected amine.

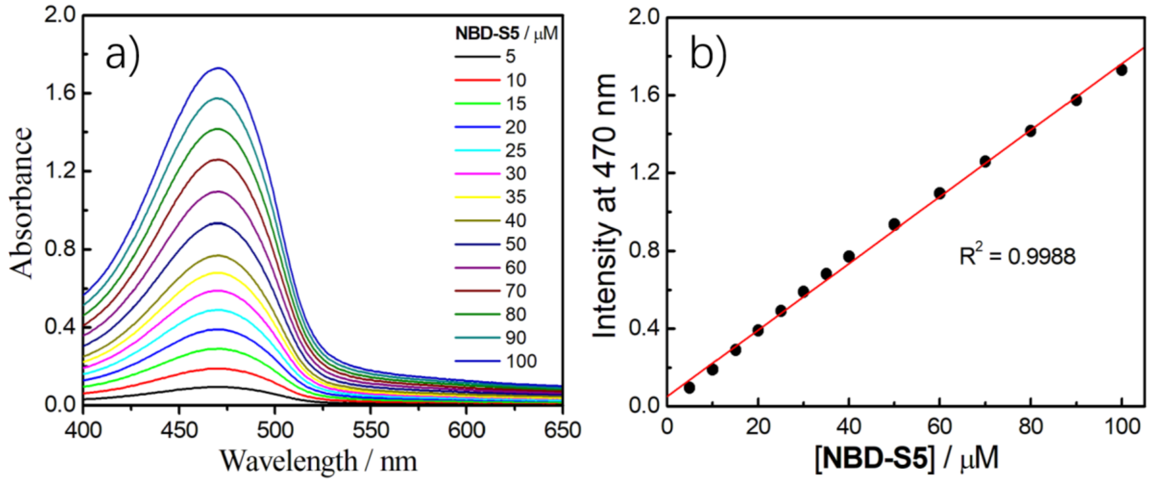

Fig. S14. (a) The absorption spectra of **NBD-S5** at different concentrations in PBS buffer (50 mM, pH = 7.4, containing 2% DMSO). (b) Solubility analysis of **NBD-S5**. Linear relationship of absorbance intensity at 470 nm and the concentration of **NBD-S5**.[11]

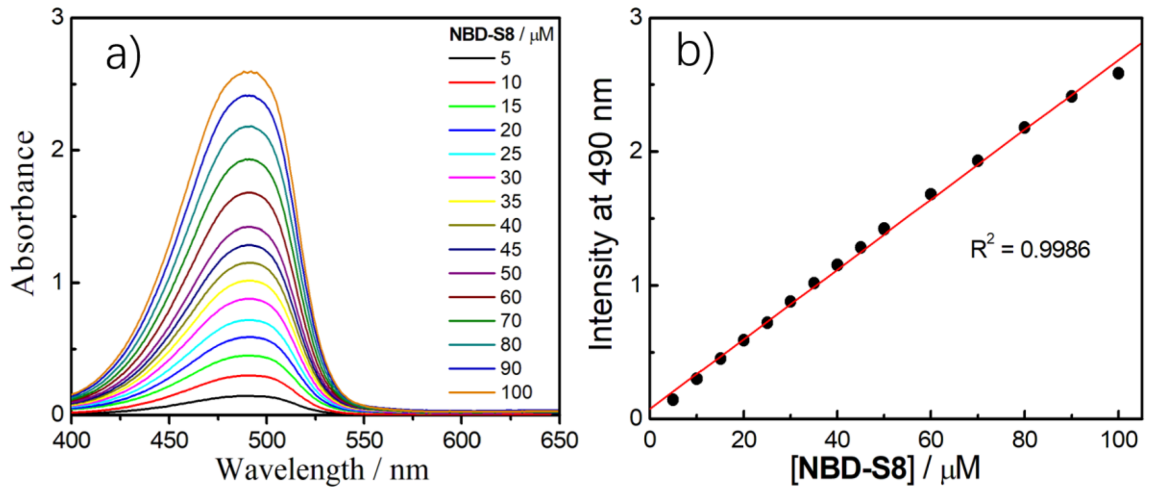

Fig. S15. (a) The absorption spectra of **NBD-S8** at different concentrations in PBS buffer (50 mM, pH = 7.4, containing 2% DMSO). (b) Solubility analysis of **NBD-S8**. Linear relationship of absorbance intensity at 490 nm and the concentration of **NBD-S8**.

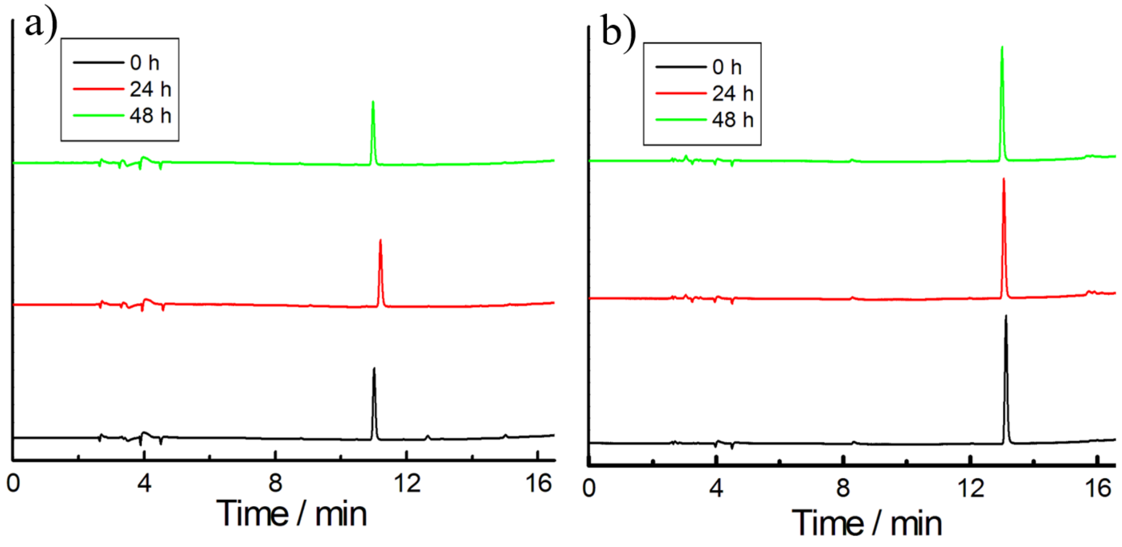

Fig. S16. Time-dependent HPLC traces of **NBD-S5** (a, 0.2 mM) and **NBD-S8** (b, 0.2 mM) in PBS buffer (50 mM, pH = 7.4, containing 10% CH_3CN).

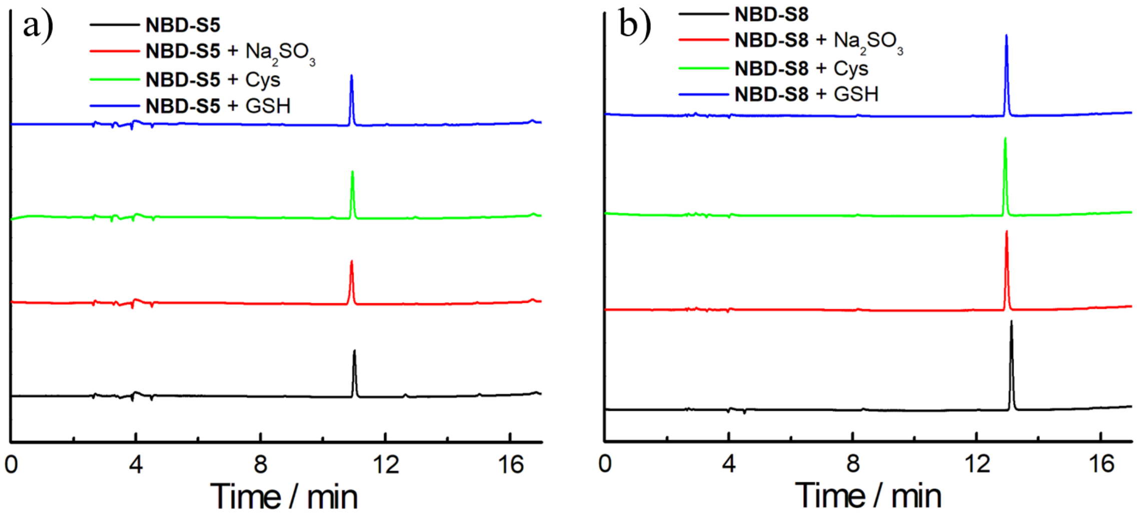

Fig. S17. HPLC traces of **NBD-S5** (a, 0.2 mM) and **NBD-S8** (b, 0.2 mM) in PBS buffer (50 mM, pH = 7.4, containing 10% CH_3CN) containing other species (Na_2SO_3: 0.2 mM; Cys or GSH: 1 mM) after 1 h incubation.

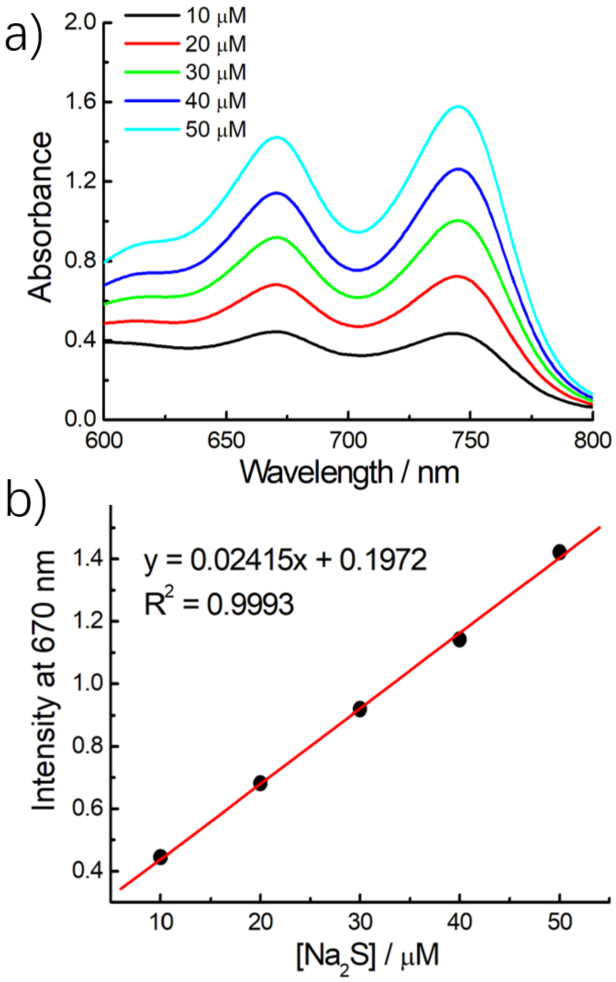

Fig. S18. MBA calibration curve generated using known concentrations of Na_2S in PBS buffer (50 mM, pH = 7.4, containing 2% DMSO), the absorbance spectra (a) and the intensity vs [Na_2S] (b).

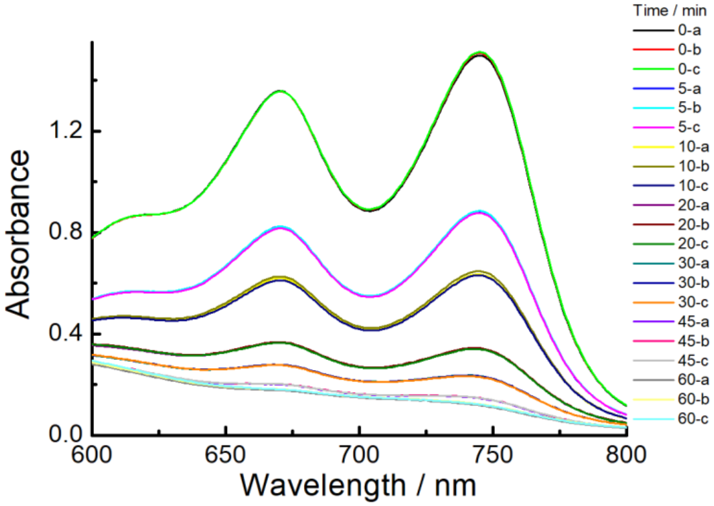

Fig. S19. The time-dependent absorbance spectra of MBA for 100 μM Na_2S in PBS buffer (50 mM, pH = 7.4, containing 2% DMSO) after addition of 110 μM **NBD-S2**; each time point was repeated triple (a-c, inset).

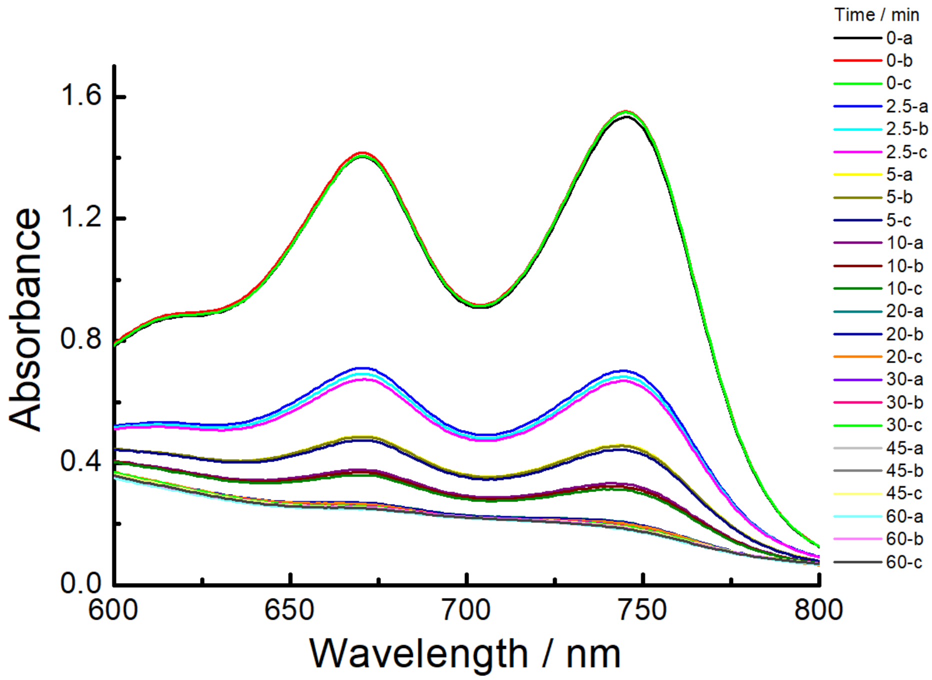

Fig. S20. The time-dependent absorbance spectra of MBA for 100 μM Na_2S in PBS buffer (50 mM, pH = 7.4, containing 2% DMSO) after addition of 110 μM **NBD-S5**; each time point was repeated triple (a-c, inset). Lines 0-c and 30-c are at above and below of the figure, respectively.

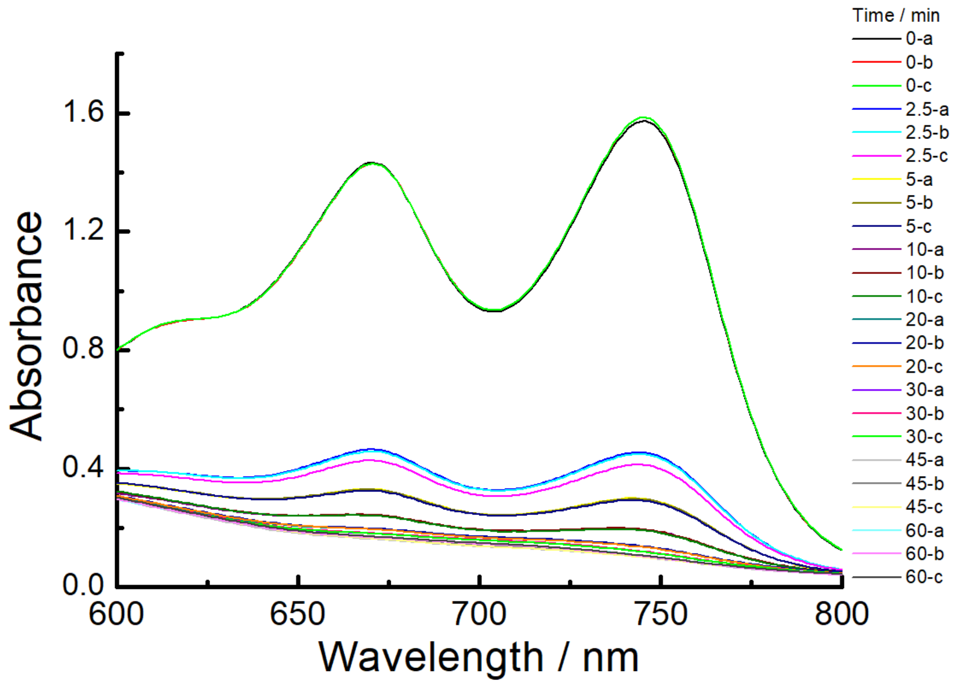

Fig. S21. The time-dependent absorbance spectra of MBA for 100 μM Na_2S in PBS buffer (50 mM, pH = 7.4, containing 2% DMSO) after addition of 110 μM **NBD-S8**; each time point was repeated triple (a-c, inset). Lines 0-c and 30-c are at above and below of the figure, respectively.

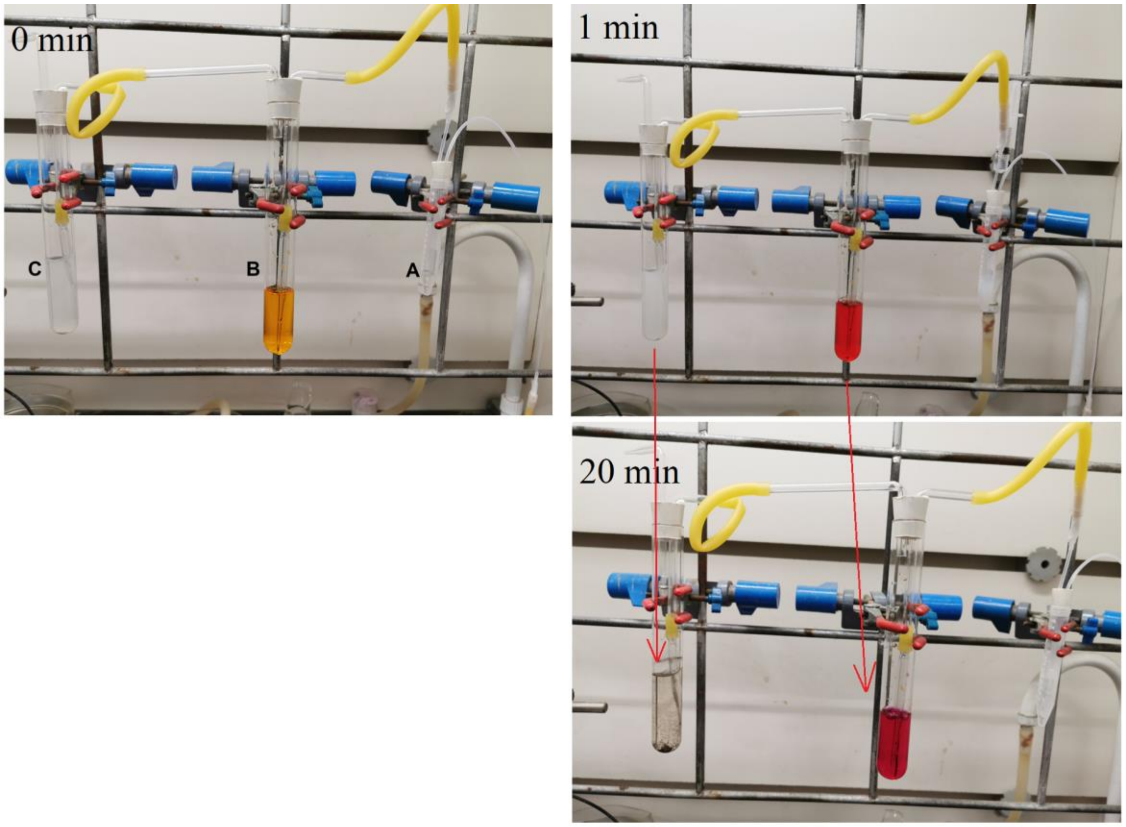

Fig. S22. The device for scavenge of H_2S in gaseous sample. A: Na_2S solution (9 mg in 5 mL H_2O); B: **NBD-S8** solution (0.2 mM, 20 mL) in PBS buffer (50 mM, pH = 7.4); C: $AgNO_3$ solution (0.1 M, 30 mL), which is used to scavenge any excess H_2S. Briefly, HCl (2 M, 5 mL) was injected into the solution A to start the reaction, and then the photos were taken at 1 min and 20 min. The solution B changed from orange to red to dark red, implying the production of NBD-SH. Black solid Ag_2S from the solution C was then produced after the color change of the solution B.

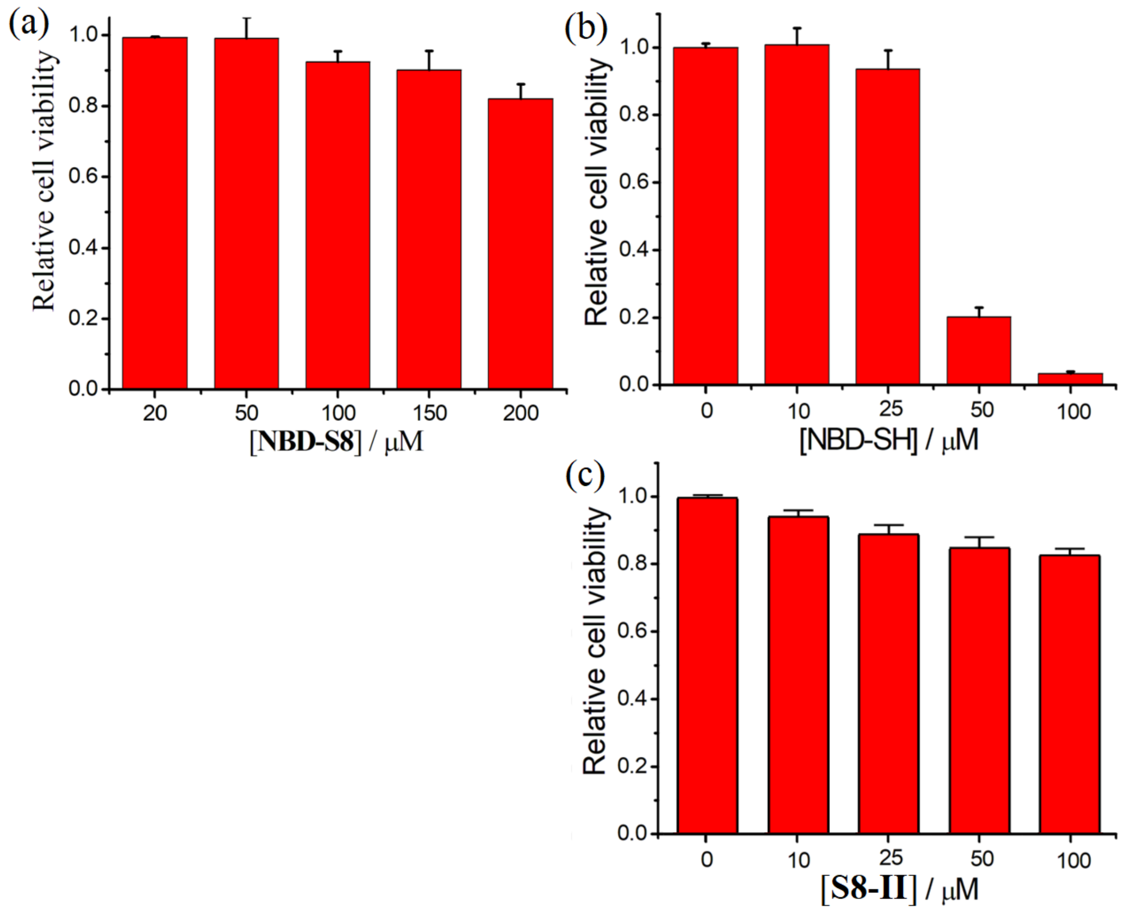

Fig. S23. MTT assay for the relative cell viability of HeLa cells treated with various concentrations of **NBD-S8** (a) or NBD-SH (b) or **S8-II** (c) for 24 h. The results are expressed as mean ± S.D. (n = 4).

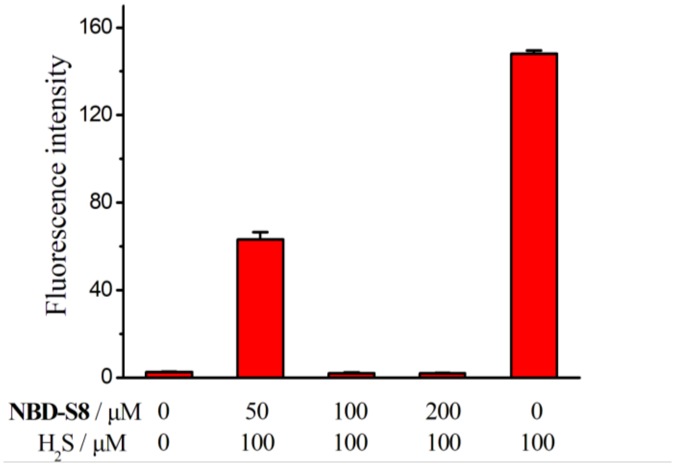

Fig. S24. Fluorescence tests to confirm the efficiency of **NBD-S8** scavenger. Increasing concentrations of scavenger **NBD-S8** (0, 50, 100, 200 µM) were added into H_2S solution (100 µM) in PBS buffer (50 mM, pH = 7.4, containing 2% DMSO) for 10 min incubation at 25 °C, respectively, and the H_2S content was measured by a H_2S probe **Cy7-NBD** (10 µM, Ex./Em. = 730 nm/800 nm) for 30 min incubation at 25 °C. The tests were performed in triplicate, and the fluorescence intensities at 800 nm were expressed as mean ± S.D. (n = 3).

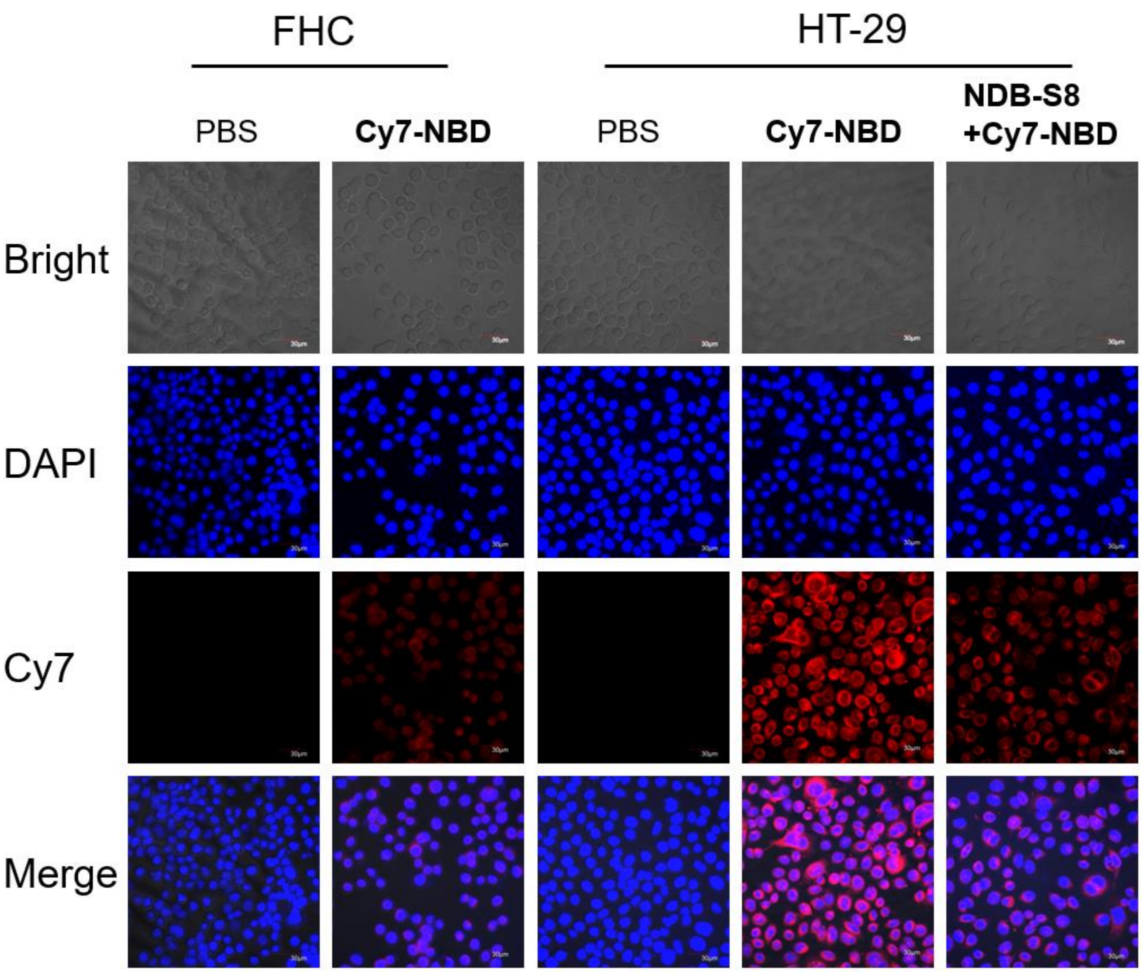

Fig. S25. Imaging of H_2S levels in FHC and HT-29 cells by the fluorescence probe **Cy7-NBD**. The HT-29 cells were pre-treated with or without **NBD-S8** for 10 min before staining with the probe. Scale bar, 30 μm.

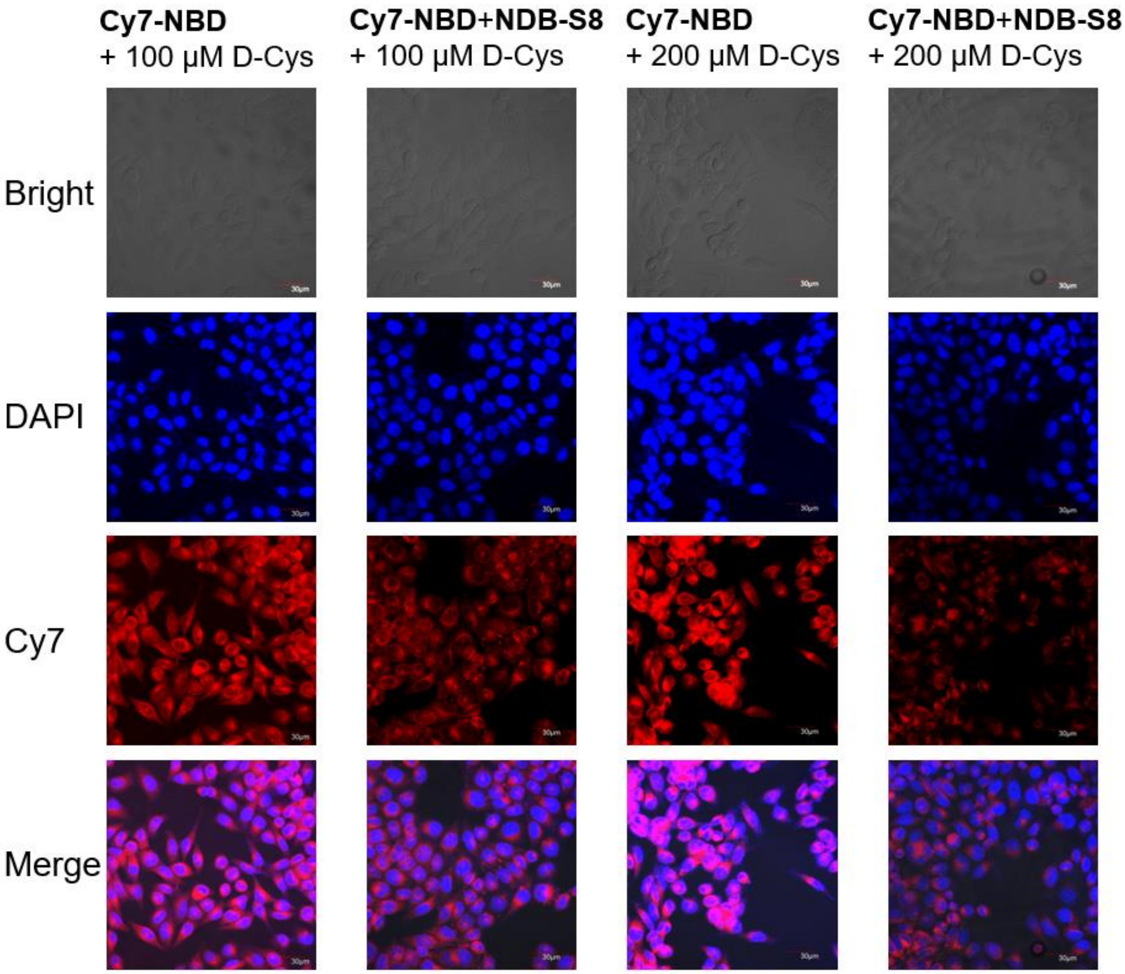

Fig. S26. Imaging of D-Cys induced H_2S in HeLa cells in the absense or presence of **NBD-S8**. H_2S scavenged by **NBD-S8** in cells was visualized via comparison of the fluorescence signals of bioimaging from **Cy7-NBD**. Scale bar, 30 μm.

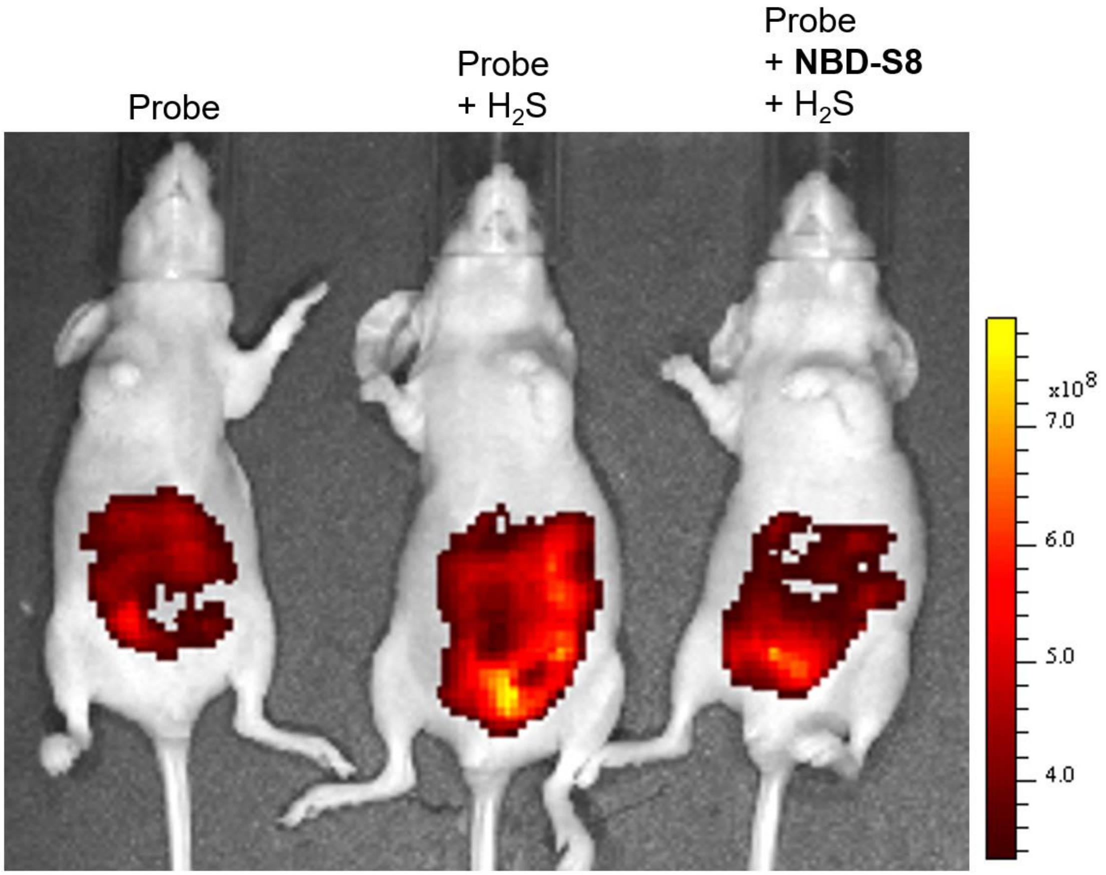

Fig. S27. Fluorescence images of exogenous H_2S by probe **Cy7-NBD** and the H_2S scavenging by **NBD-S8** in mice. Mice were treated with different reagents via intraperitoneal injection: one group was injected with **Cy7-NBD** only; the second group was injected with Na_2S first, then with **Cy7-NBD**; and the third group was injected with Na_2S followed by **NBD-S8**, and then with **Cy7-NBD**.

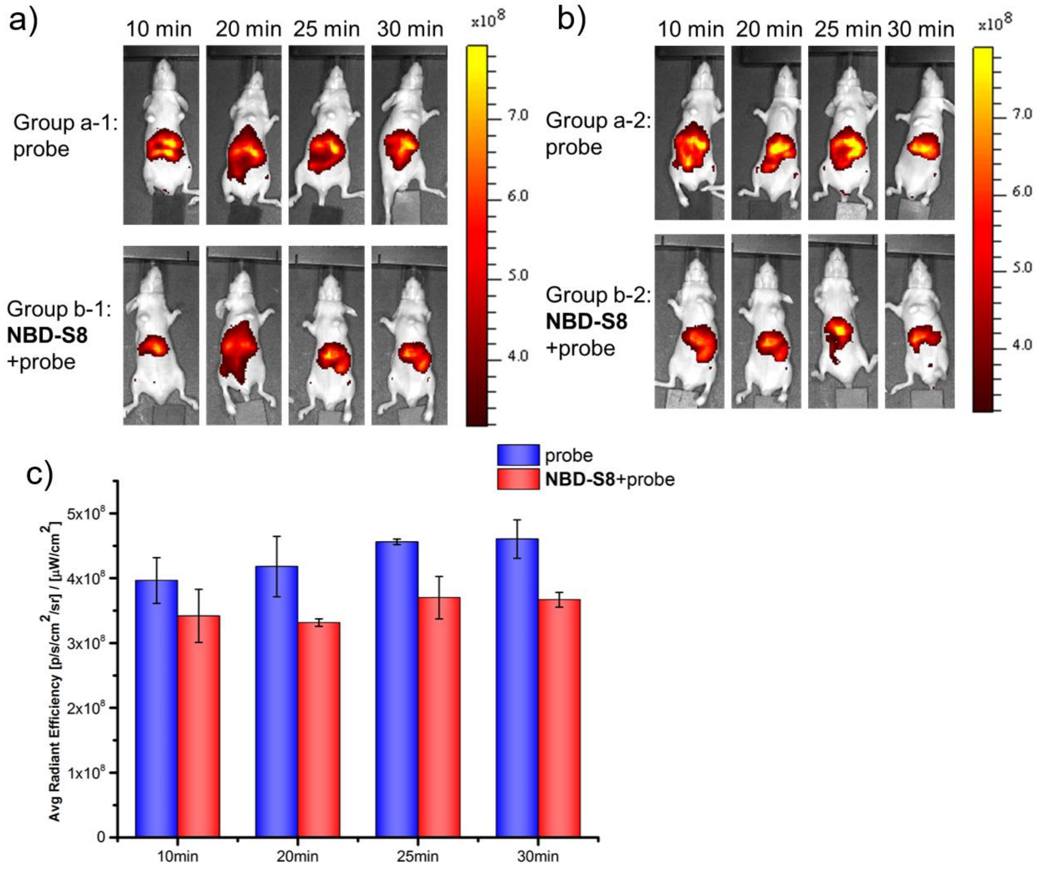

Fig. S28. (a, b) Two repeated experiments of fluorescence images of mice which were treated with **NBD-S8** and **Cy7-NBD** via tail vein injection as that in Fig. 8. (c) Time-dependent average radiant efficiency in the ROI (region of interest) of each image in mice that were treated with or without the H_2S scavenger **NBD-S8**. Data are shown as the mean ± SD.

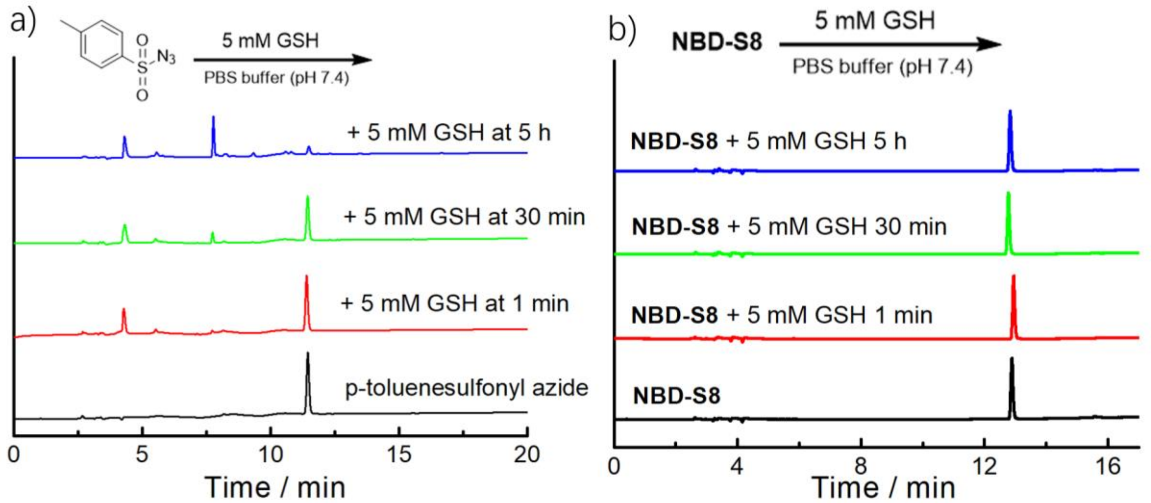

Fig. S29. Time-dependent HPLC traces of *p*-toluenesulfonyl azide (a, 0.3 mM) and **NBD-S8** (b, 0.3 mM) in PBS buffer (50 mM, pH = 7.4, containing 50% CH_3CN) containing 5 mM GSH at 25 ºC. Upon 5 h incubation, the results indicated that more than 90% *p*-toluenesulfonyl azide was decomposed under such conditions, but **NBD-S8** was stable.

8. Supporting NMR and MS spectra

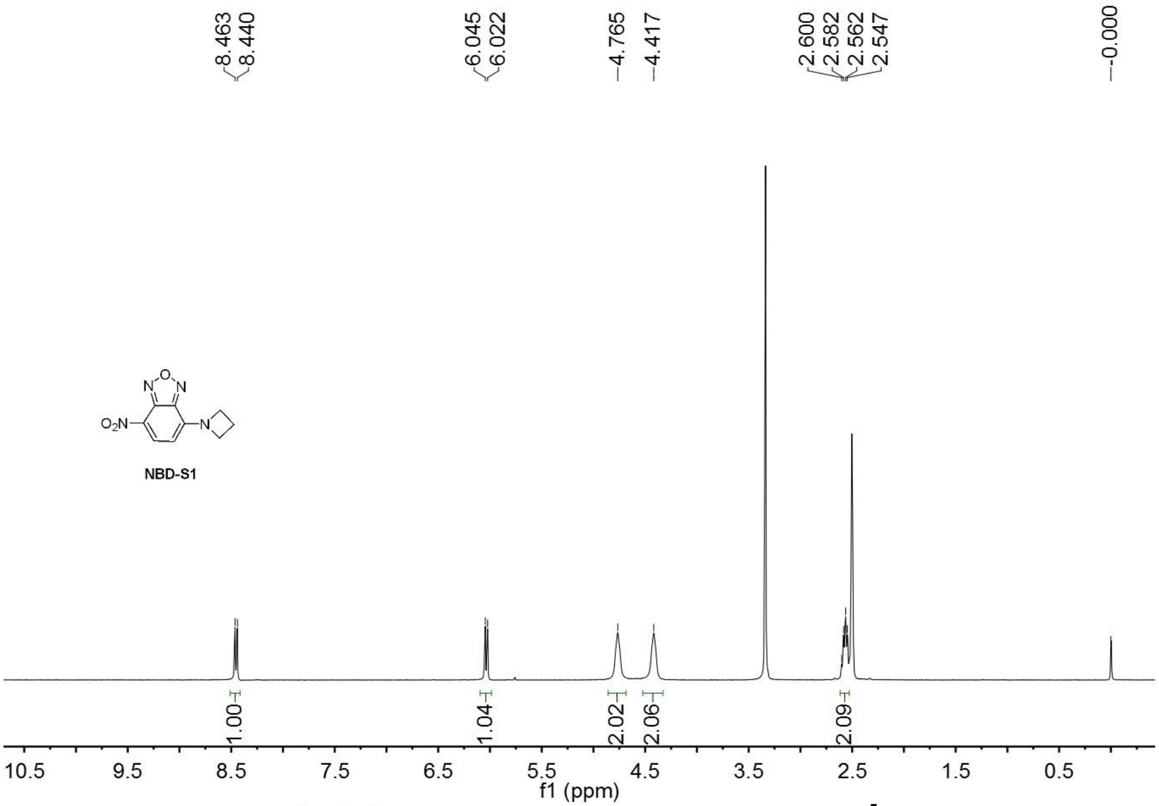

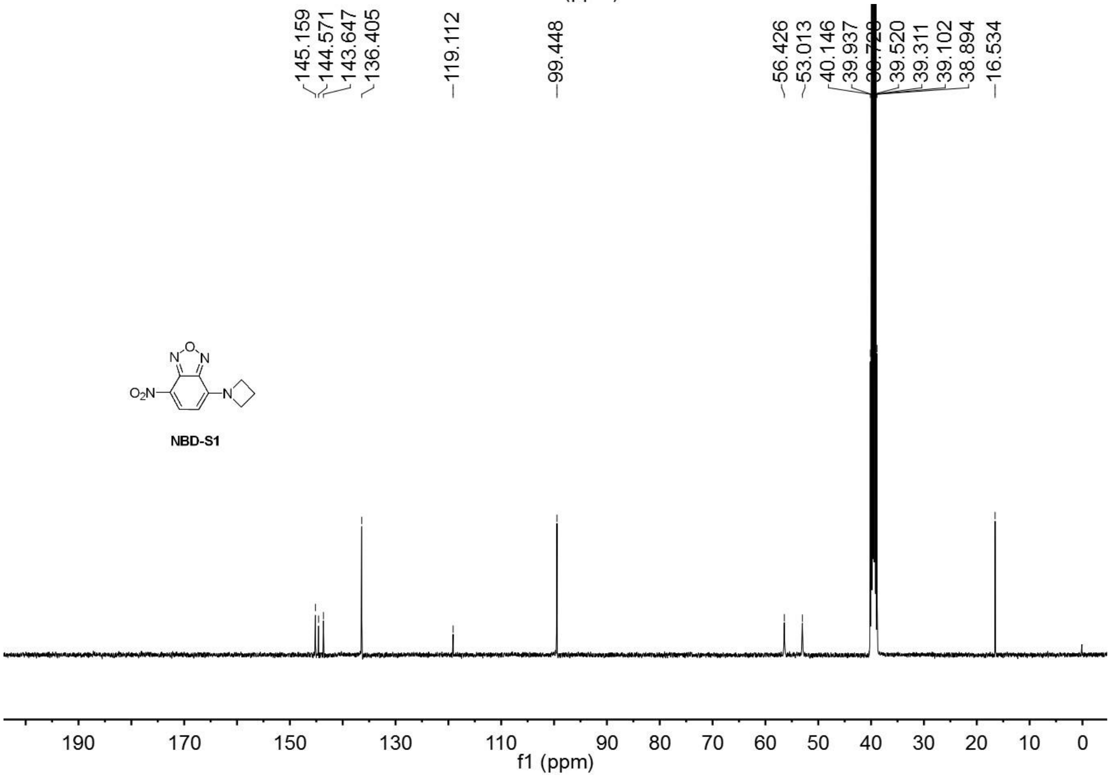

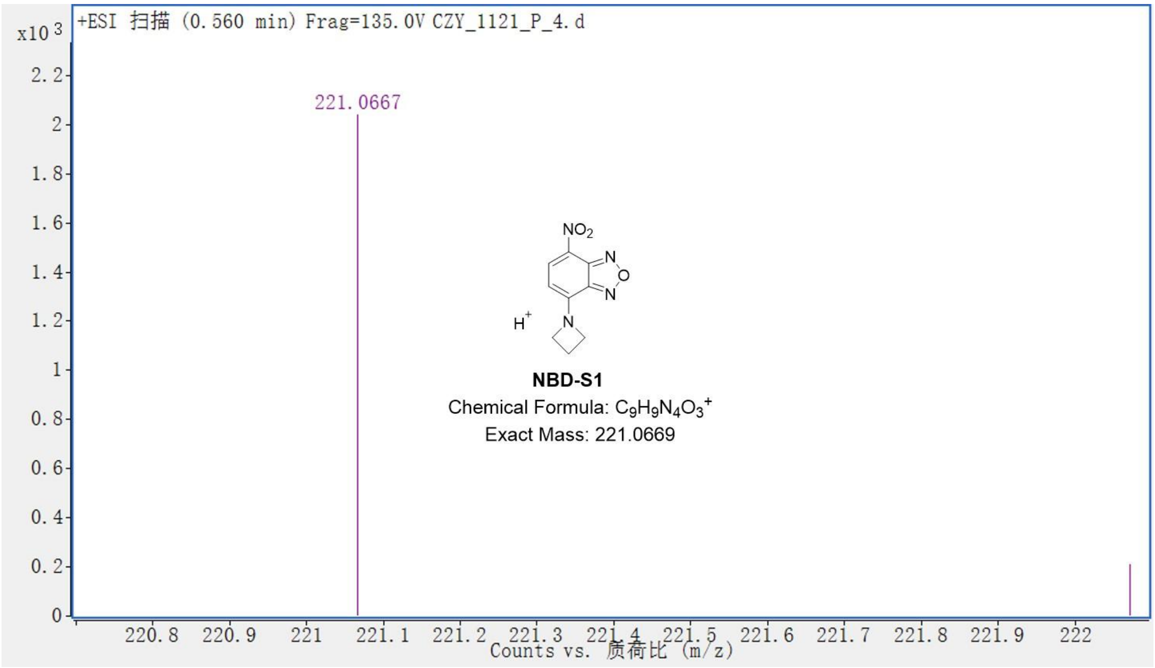
+ESI 扫描 (0.560 min) Frag=135.0V CZY_1121_P_4.d
x10 3
221.0667
NO2
N
O
N
H+
N
NBD-S1
Chemical Formula: C9H9N4O3+
Exact Mass: 221.0669
Counts vs. 质荷比 (m/z)

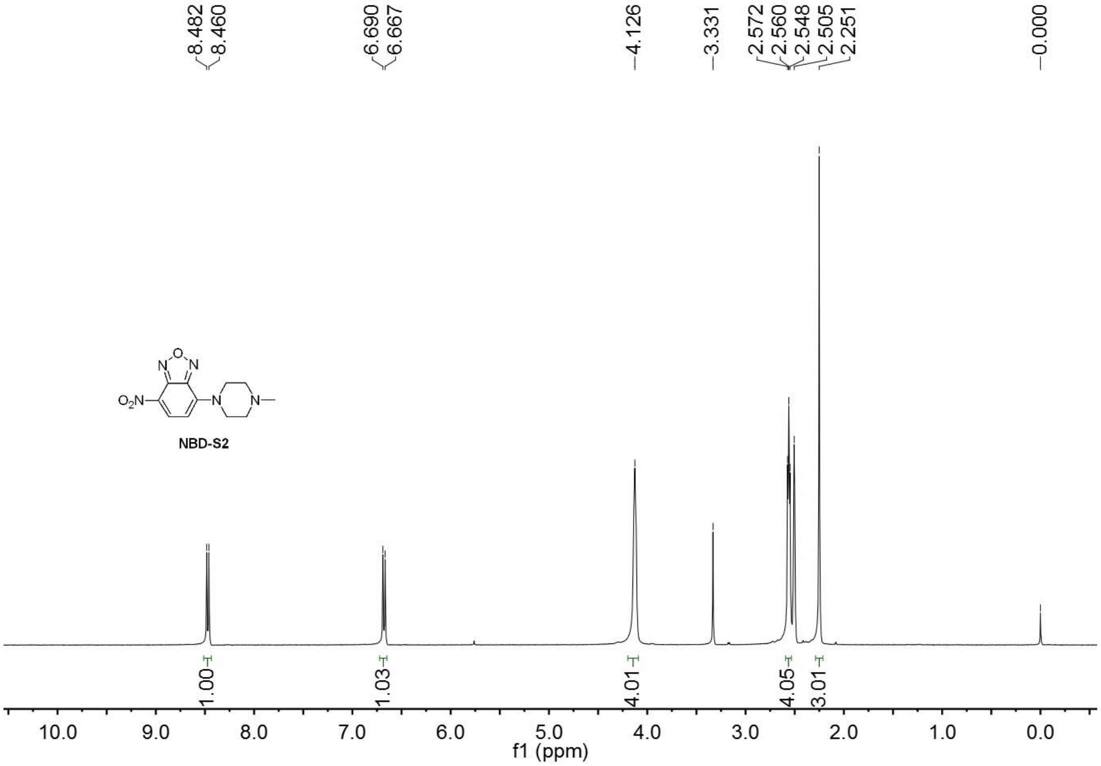
8.482
8.460
6.690
6.667
4.126
3.331
2.572
2.560
2.548
2.505
2.251
0.000
NBD-S2
1.00
1.03
4.01
4.05
3.01
f1 (ppm)

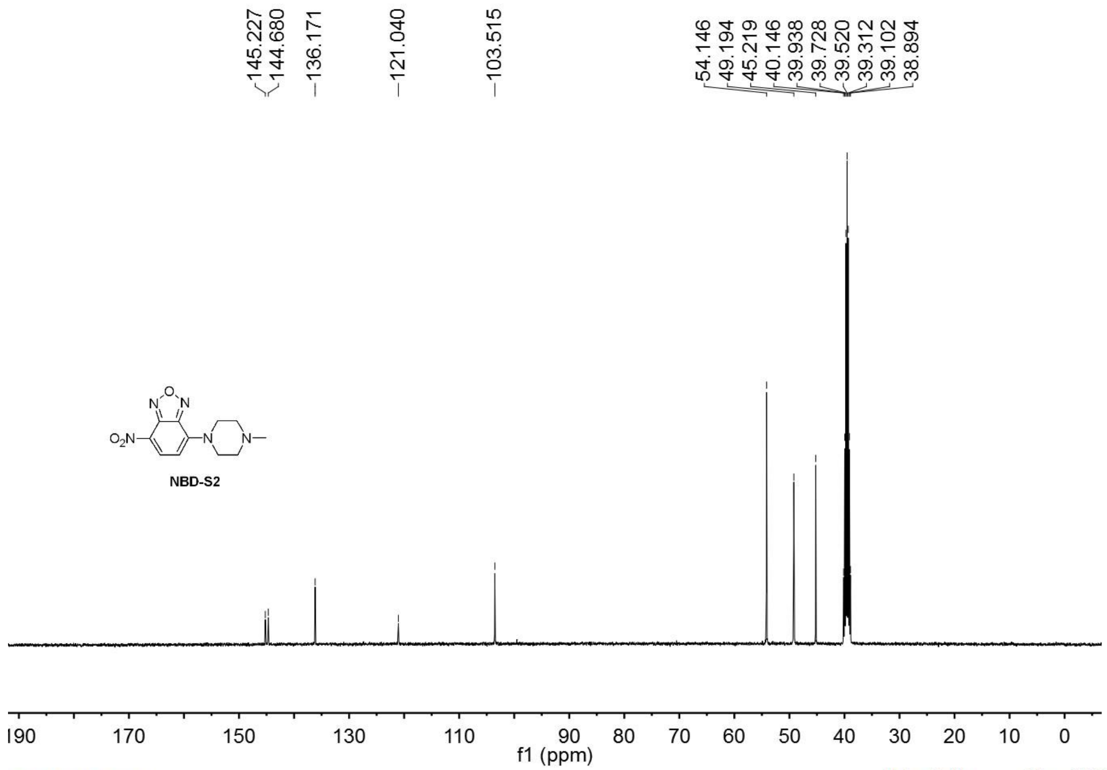
145.227
144.680
136.171
121.040
103.515
54.146
49.194
45.219
40.146
39.938
39.728
39.520
39.312
39.102
38.894
NBD-S2
190 170 150 130 110 90 80 70 60 50 40 30 20 10 0
f1 (ppm)

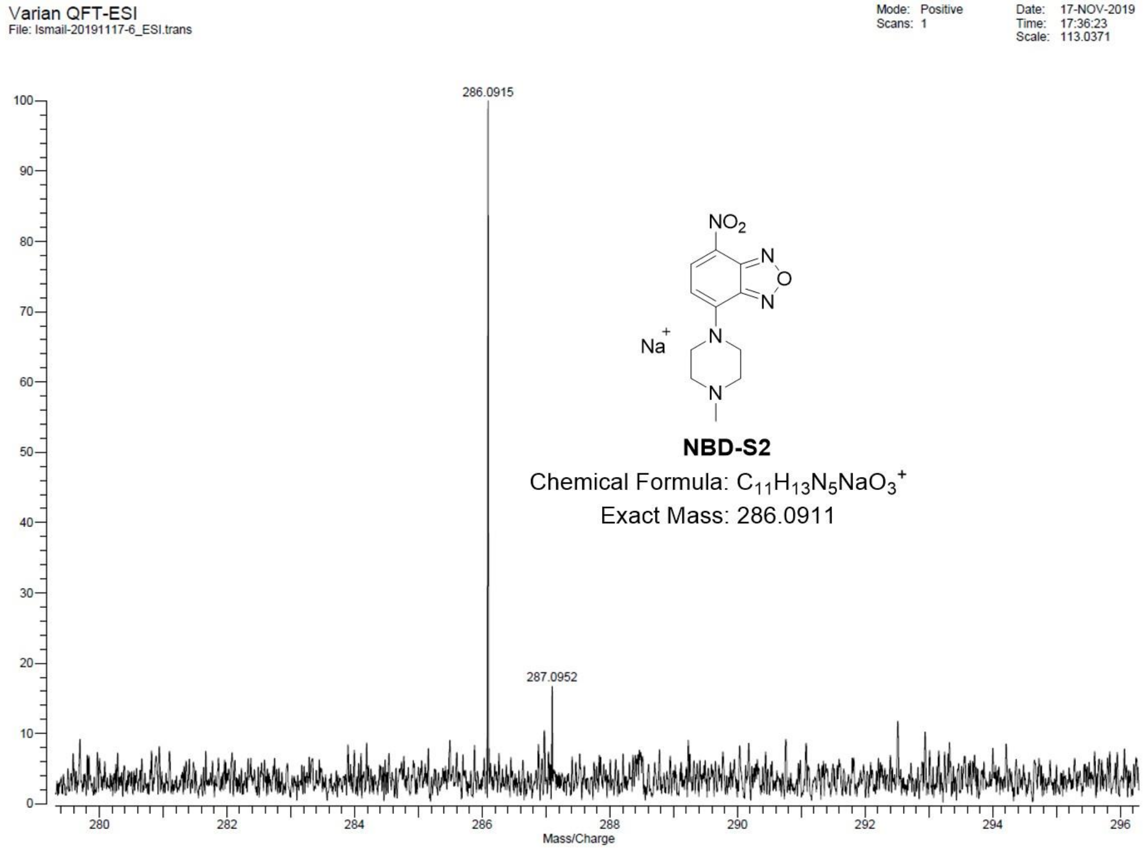
Varian QFT-ESI
File: Ismail-20191117-6_ESI.trans
Mode: Positive
Scans: 1
Date: 17-NOV-2019
Time: 17:36:23
Scale: 113.0371
286.0915
287.0952
NBD-S2
Chemical Formula: $C_{11}H_{13}N_5NaO_3^+$
Exact Mass: 286.0911
Mass/Charge

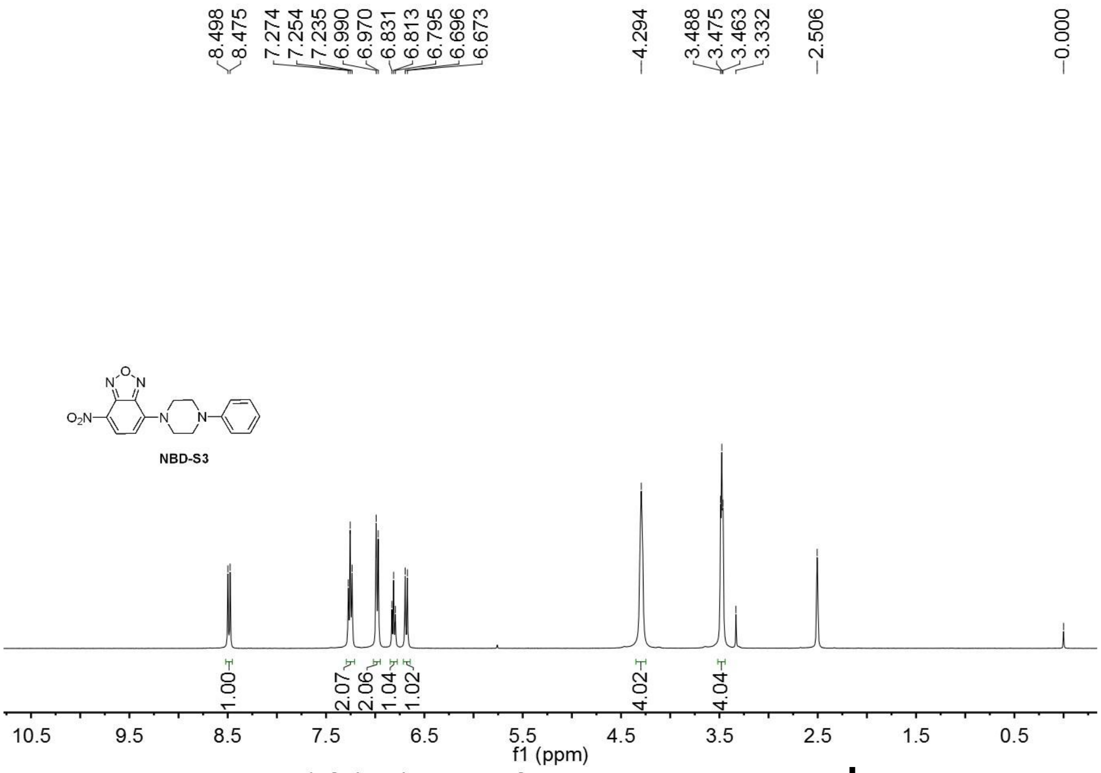
8.498
8.475
7.274
7.254
7.235
6.990
6.970
6.831
6.813
6.795
6.696
6.673
4.294
3.488
3.475
3.463
3.332
2.506
0.000
NBD-S3
1.00
2.07
2.06
1.04
1.02
4.02
4.04
10.5
9.5
8.5
7.5
6.5
5.5
4.5
3.5
2.5
1.5
0.5
f1 (ppm)

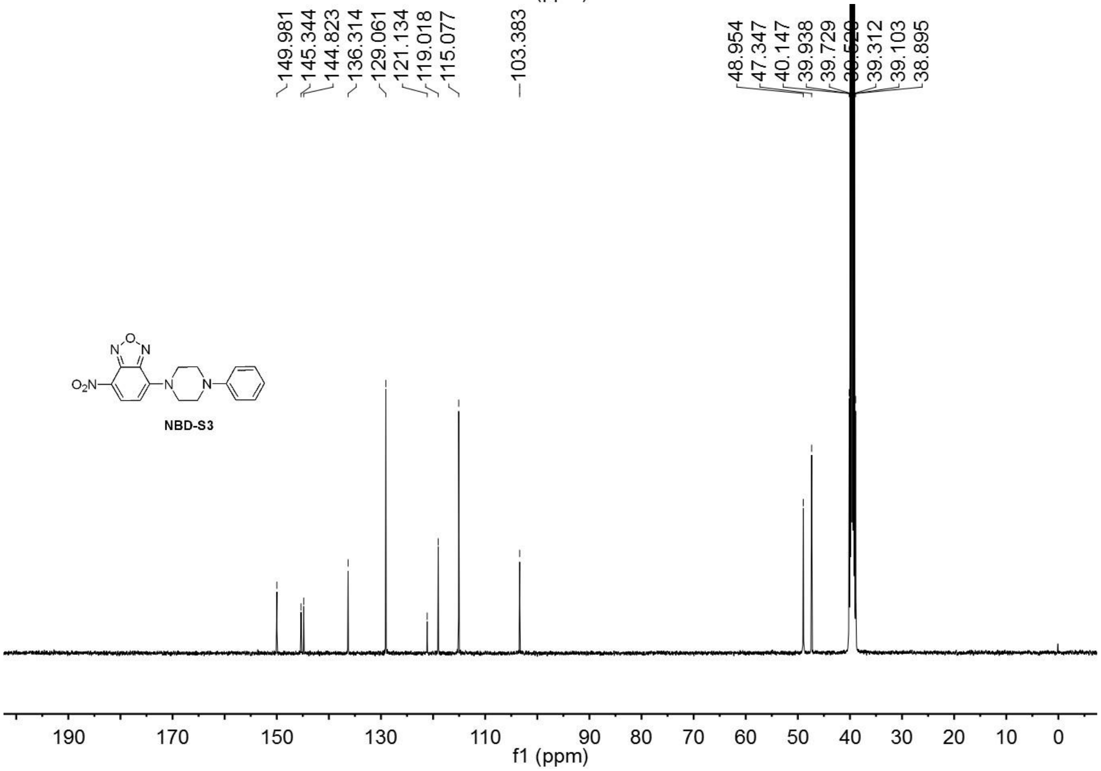
149.981
145.344
144.823
136.314
129.061
121.134
119.018
115.077
103.383
48.954
47.347
40.147
39.938
39.729
39.312
39.103
38.895
NBD-S3
190
170
150
130
110
90
80
70
60
50
40
30
20
10
0
f1 (ppm)

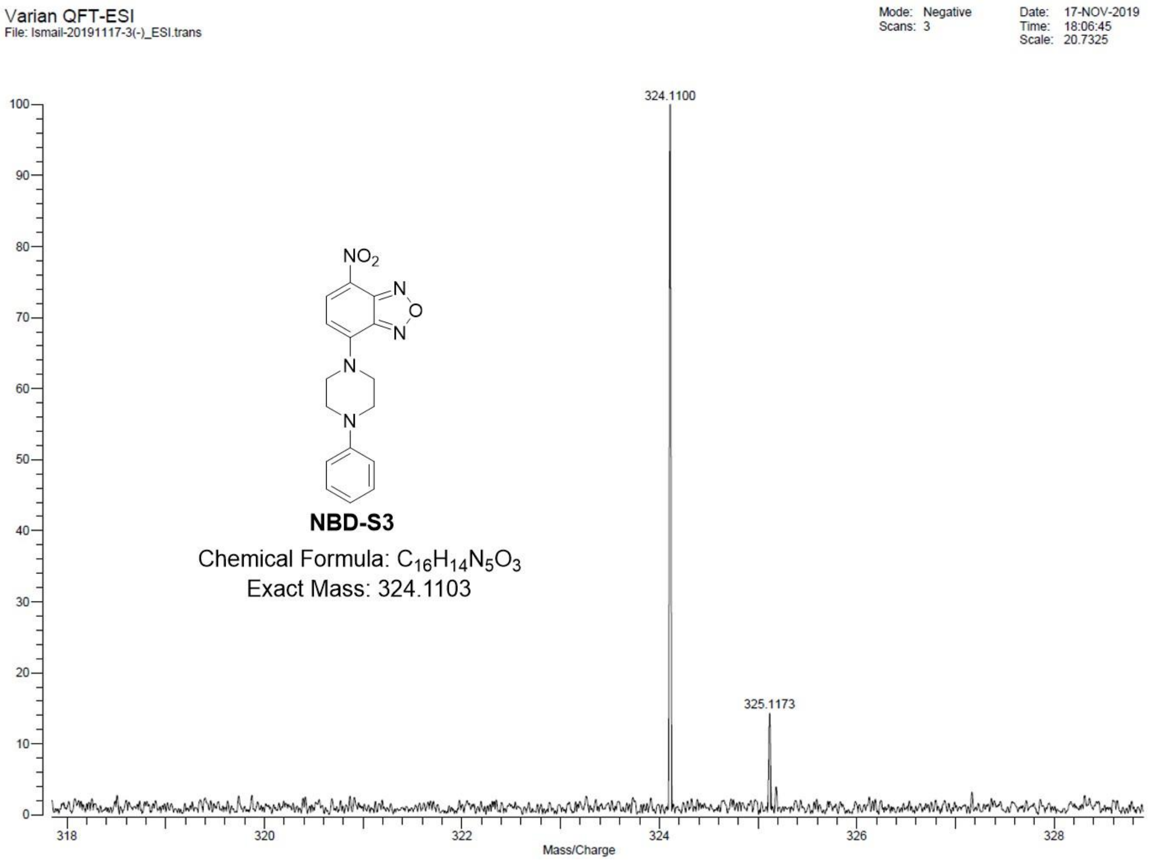
Varian QFT-ESI
File: Ismail-20191117-3(-)_ESI.trans
Mode: Negative
Scans: 3
Date: 17-NOV-2019
Time: 18:06:45
Scale: 20.7325
324.1100
325.1173
NBD-S3
Chemical Formula: $C_{16}H_{14}N_5O_3$
Exact Mass: 324.1103
Mass/Charge

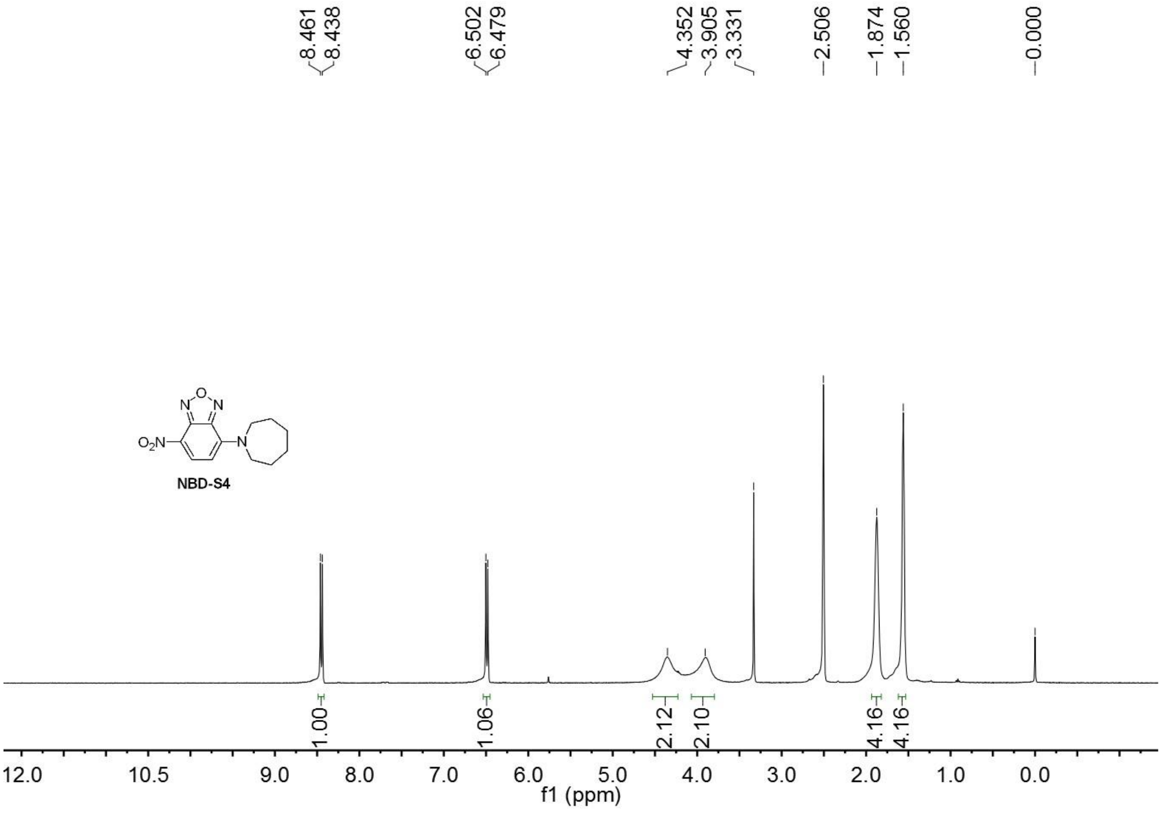
8.461
8.438
6.502
6.479
4.352
3.905
3.331
2.506
1.874
1.560
0.000
NBD-S4
1.00
1.06
2.12
2.10
4.16
4.16
f1 (ppm)

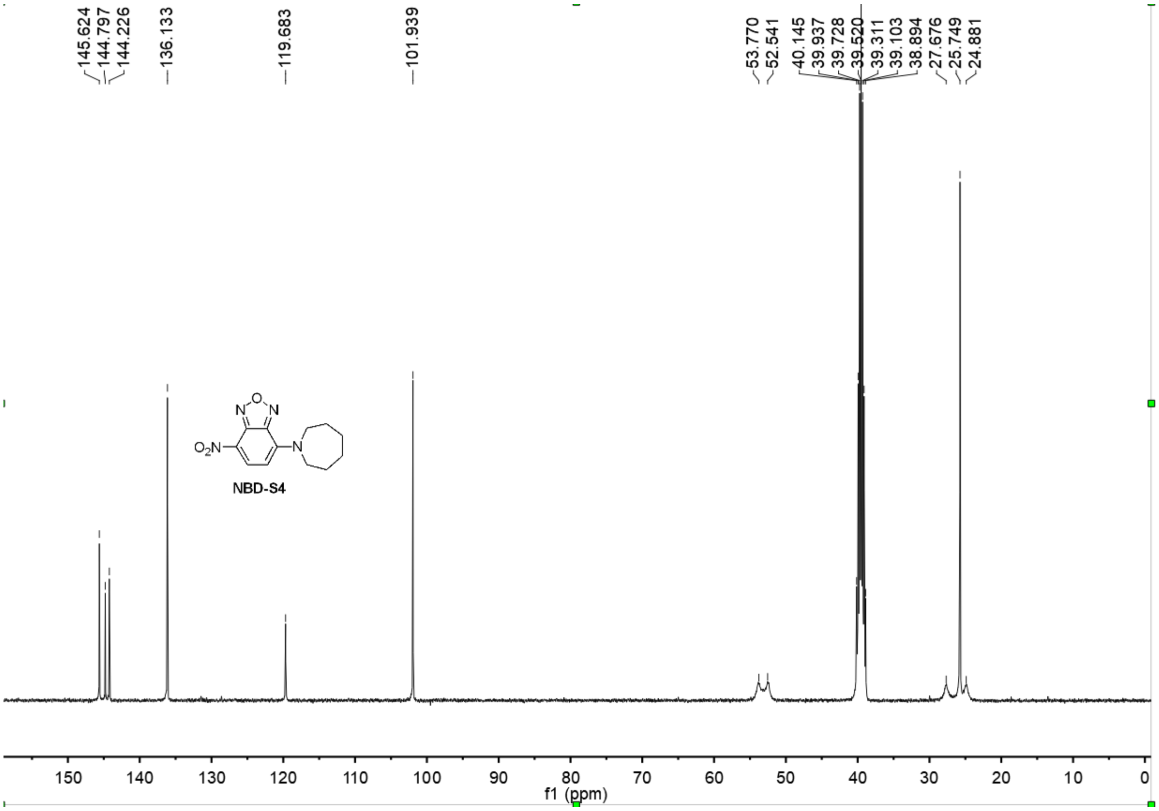
145.624
144.797
144.226
136.133
119.683
101.939
53.770
52.541
40.145
39.937
39.728
39.520
39.311
39.103
38.894
27.676
25.749
24.881
NBD-S4
f1 (ppm)

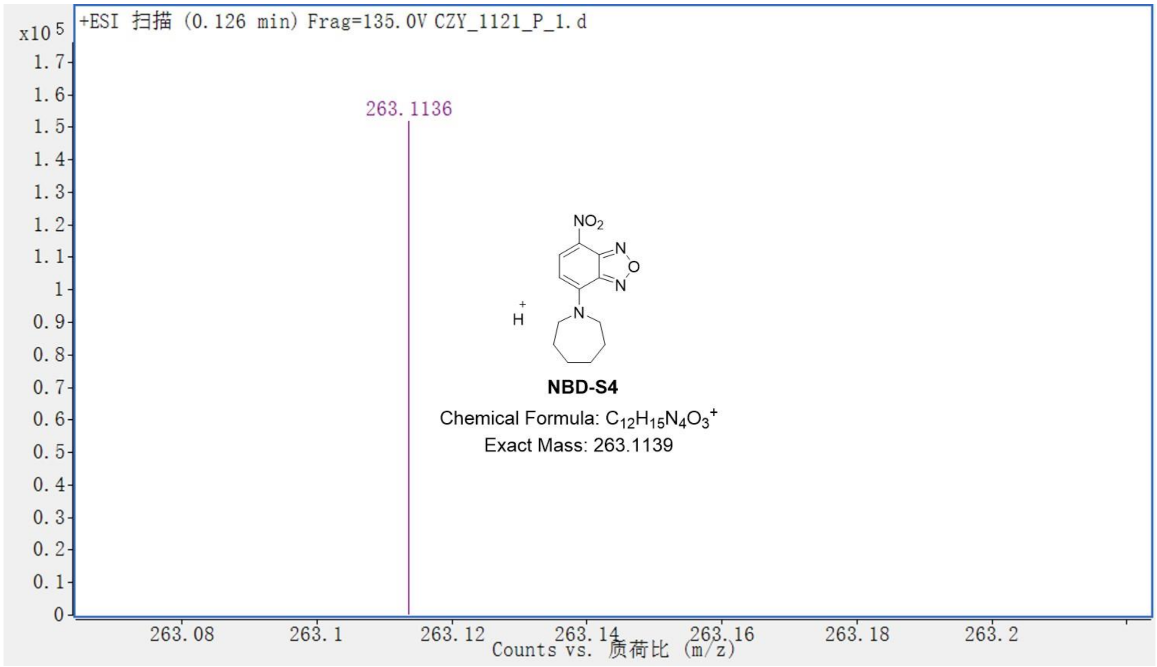
+ESI 扫描 (0.126 min) Frag=135.0V CZY_1121_P_1.d
x10 5
263.1136
NBD-S4
Chemical Formula: C12H15N4O3+
Exact Mass: 263.1139
Counts vs. 质荷比 (m/z)

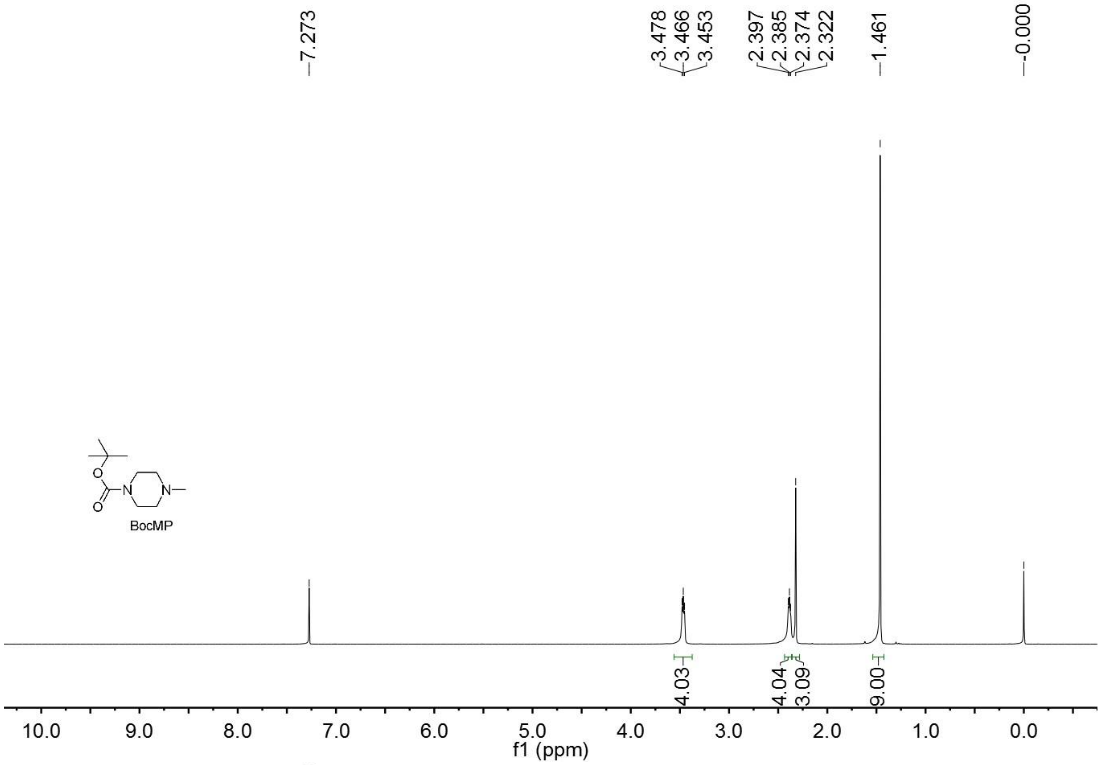
7.273
3.478
3.466
3.453
2.397
2.385
2.374
2.322
1.461
-0.000
BocMP
4.03
4.04
3.09
9.00
10.0 9.0 8.0 7.0 6.0 5.0 4.0 3.0 2.0 1.0 0.0
f1 (ppm)

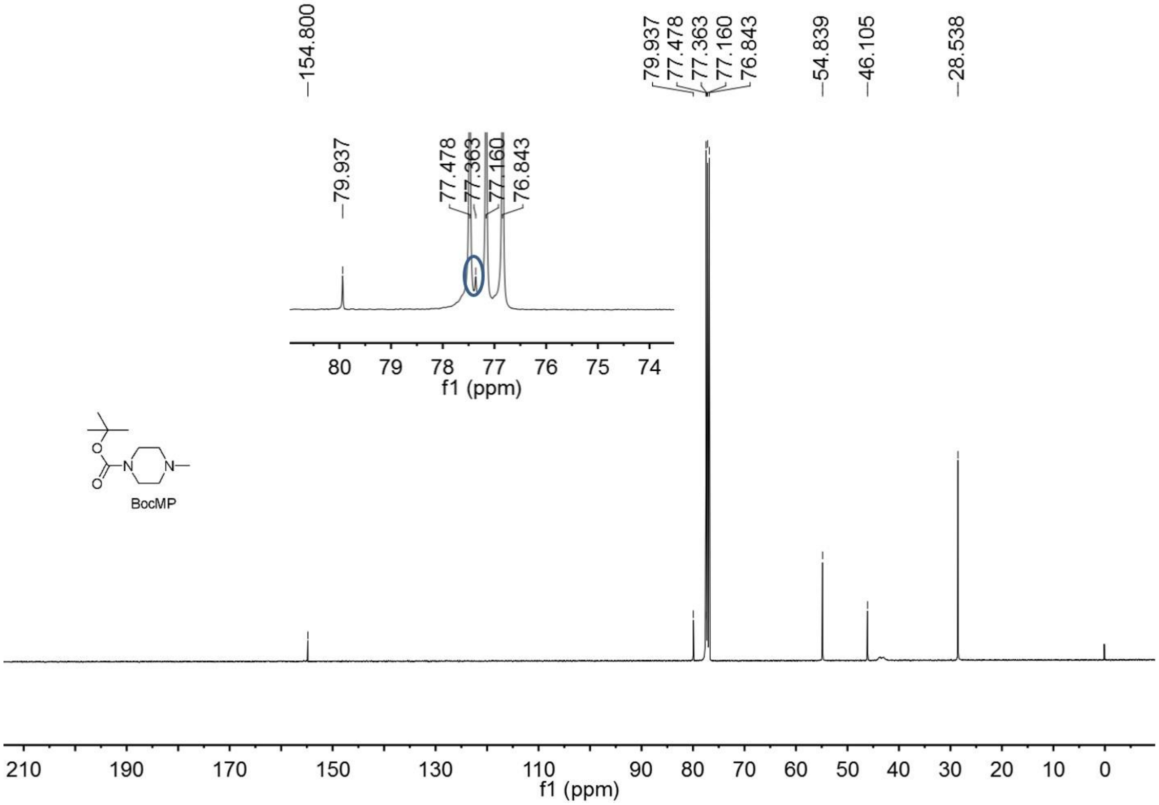
154.800
79.937
77.478
77.363
77.160
76.843
54.839
46.105
28.538
79.937
77.478
77.363
77.160
76.843
80 79 78 77 76 75 74
f1 (ppm)
BocMP
210 190 170 150 130 110 90 80 70 60 50 40 30 20 10 0
f1 (ppm)

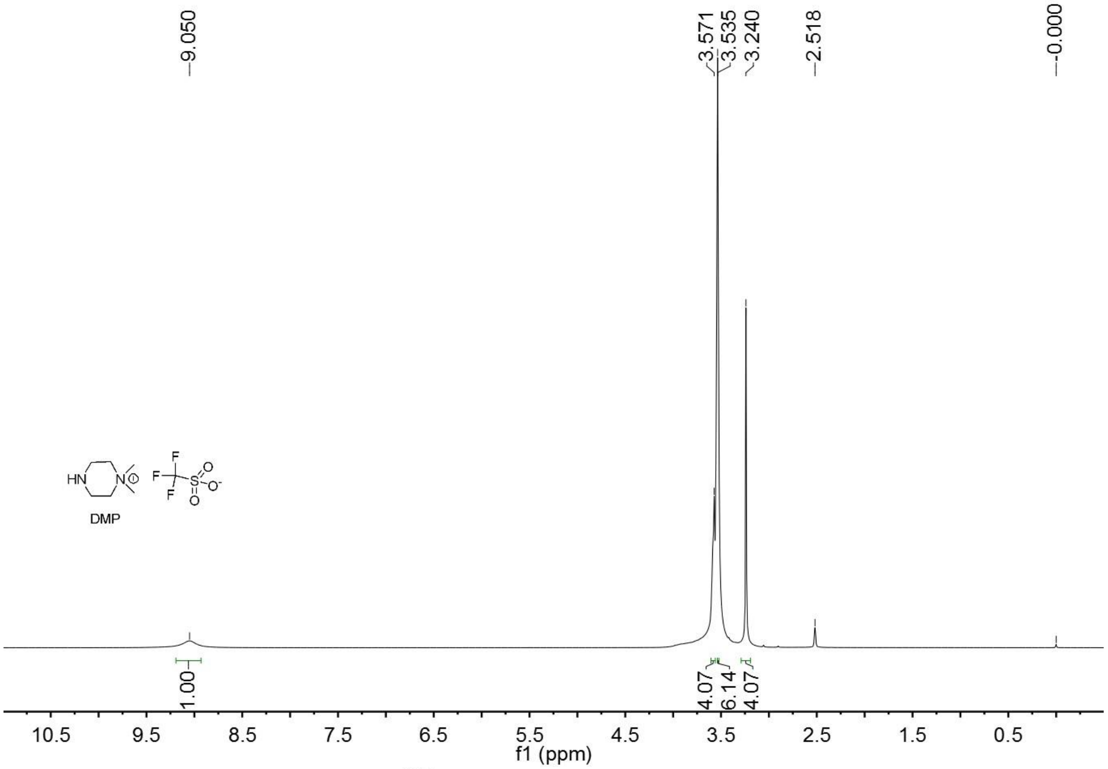
9.050
3.571
3.535
3.240
2.518
-0.000
HN
N
F
S
O
O
O⁻
DMP
1.00
4.07
6.14
4.07
10.5
9.5
8.5
7.5
6.5
5.5
4.5
3.5
2.5
1.5
0.5
f1 (ppm)

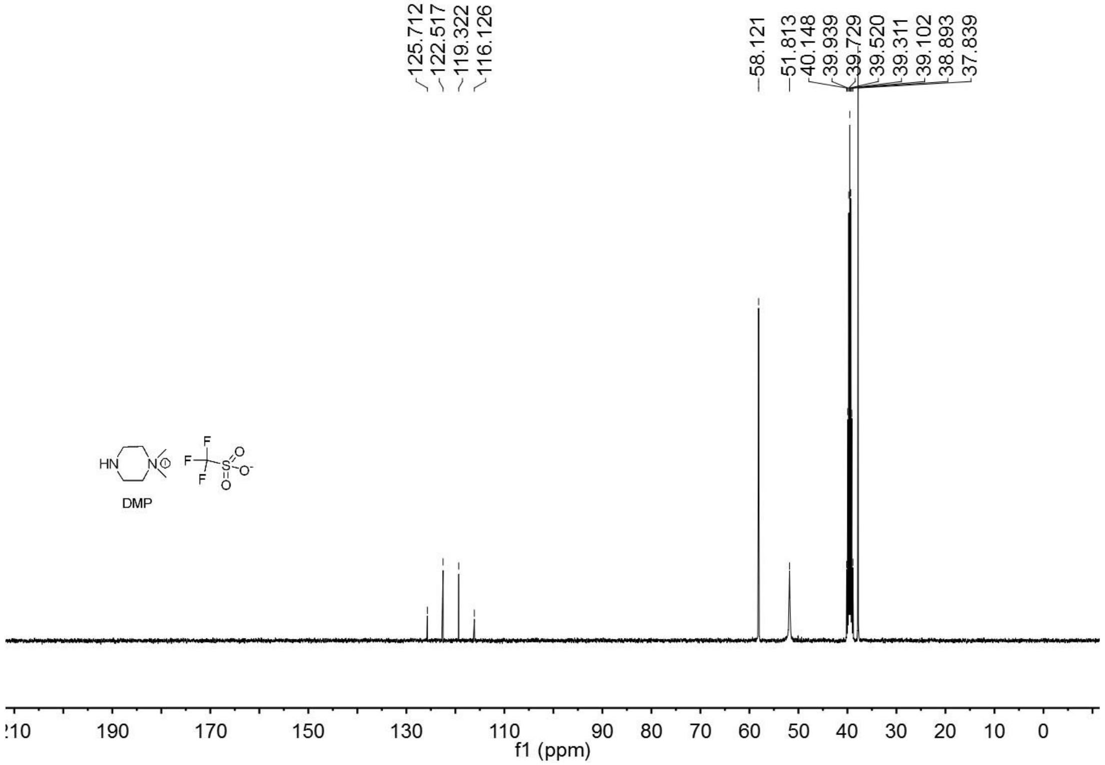
125.712
122.517
119.322
116.126
58.121
51.813
40.148
39.939
39.729
39.520
39.311
39.102
38.893
37.839
DMP
190
170
150
130
110
90
80
70
60
50
40
30
20
10
0
f1 (ppm)

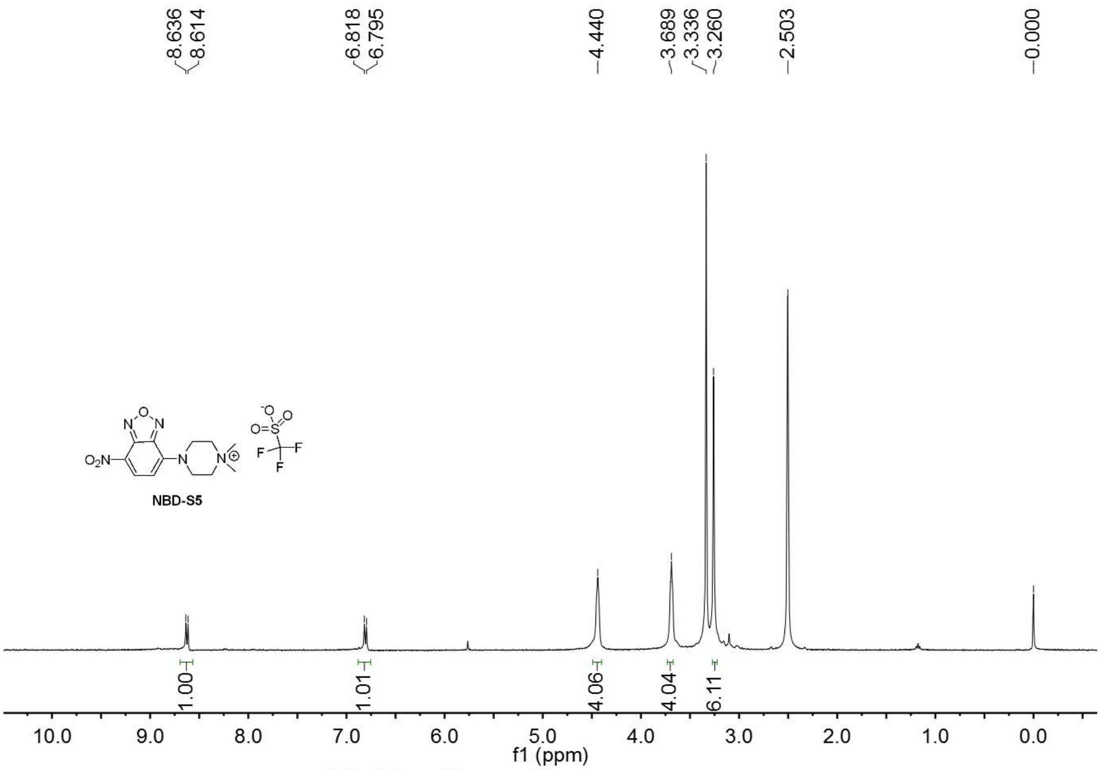
8.636
8.614
6.818
6.795
4.440
3.689
3.336
3.260
2.503
0.000
NBD-S5
1.00
1.01
4.06
4.04
6.11
10.0 9.0 8.0 7.0 6.0 5.0 4.0 3.0 2.0 1.0 0.0
f1 (ppm)

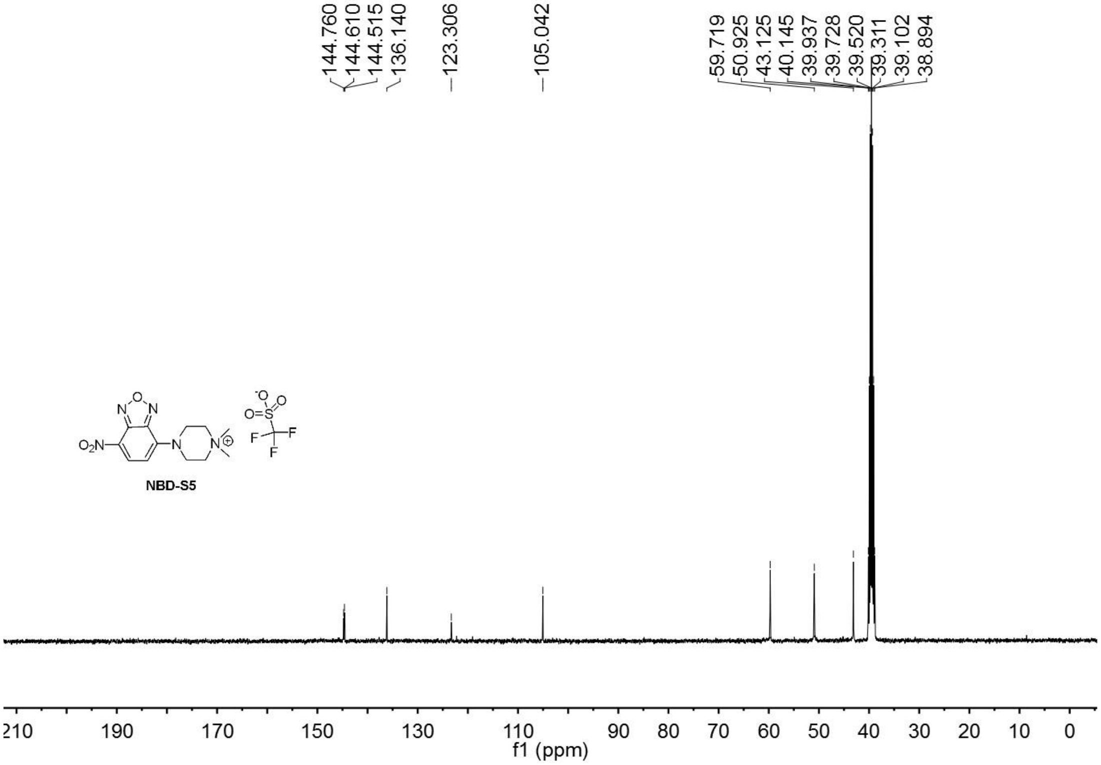
144.760
144.610
144.515
136.140
123.306
105.042
59.719
50.925
43.125
40.145
39.937
39.728
39.520
39.311
39.102
38.894
NBD-S5
210 190 170 150 130 110 90 80 70 60 50 40 30 20 10 0
f1 (ppm)

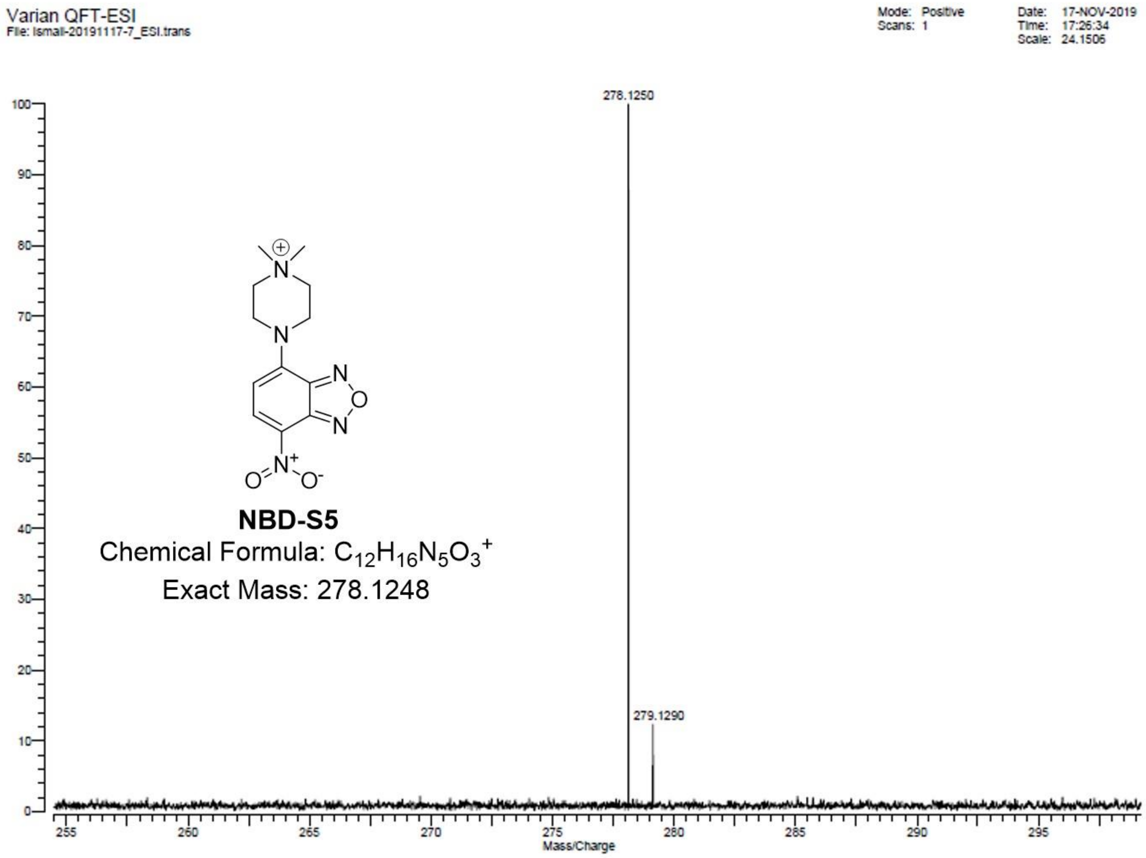
Varian QFT-ESI
File: Ismail-20191117-7_ESI.trans
Mode: Positive
Scans: 1
Date: 17-NOV-2019
Time: 17:26:34
Scale: 24.1506
278.1250
279.1290
NBD-S5
Chemical Formula: C12H16N5O3+
Exact Mass: 278.1248
Mass/Charge

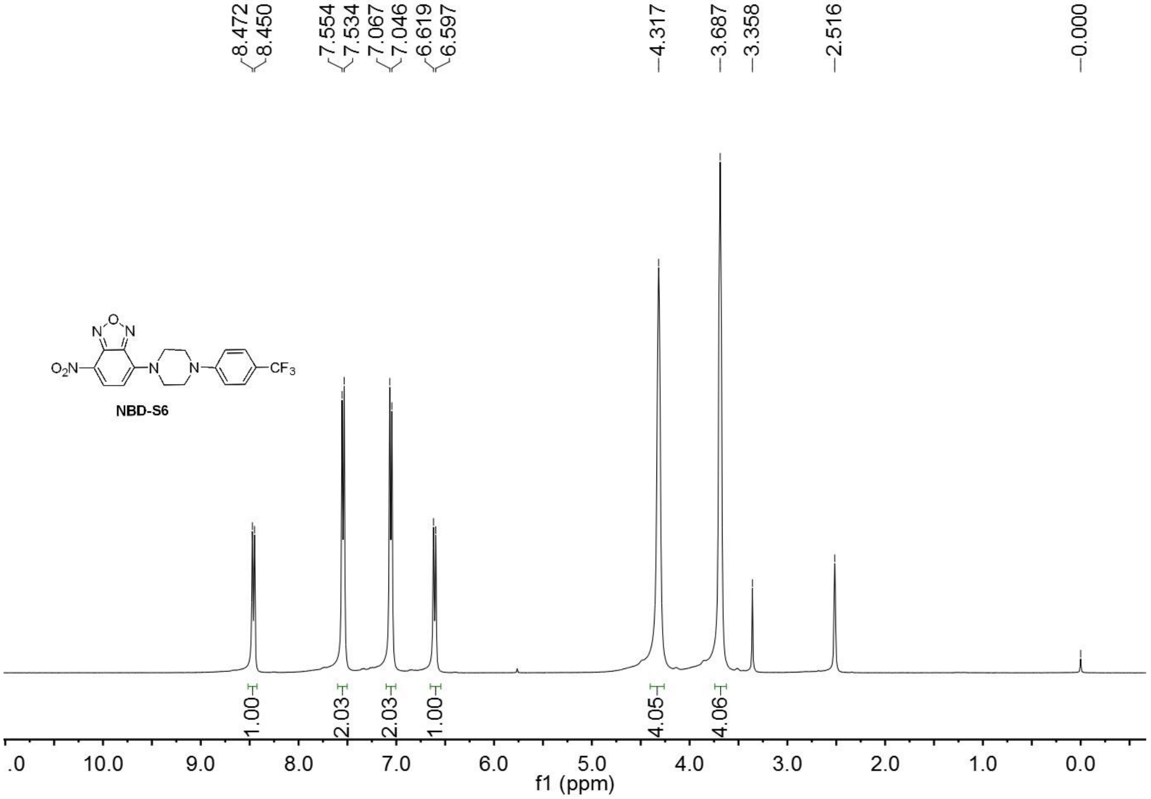
8.472
8.450
7.554
7.534
7.067
7.046
6.619
6.597
4.317
3.687
3.358
2.516
0.000
NBD-S6
1.00
2.03
2.03
1.00
4.05
4.06
f1 (ppm)

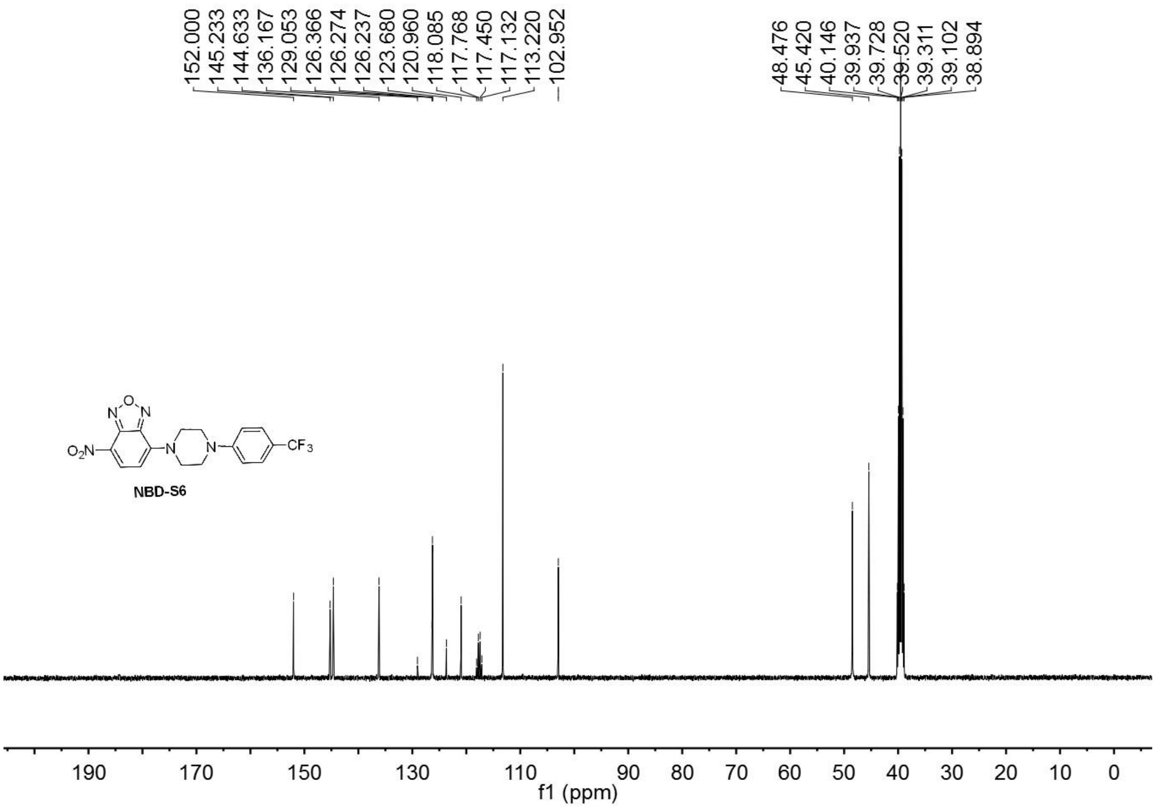
152.000
145.233
144.633
136.167
129.053
126.366
126.274
126.237
123.680
120.960
118.085
117.768
117.450
117.132
113.220
102.952
48.476
45.420
40.146
39.937
39.728
39.520
39.311
39.102
38.894
NBD-S6
190 170 150 130 110 90 80 70 60 50 40 30 20 10 0
f1 (ppm)

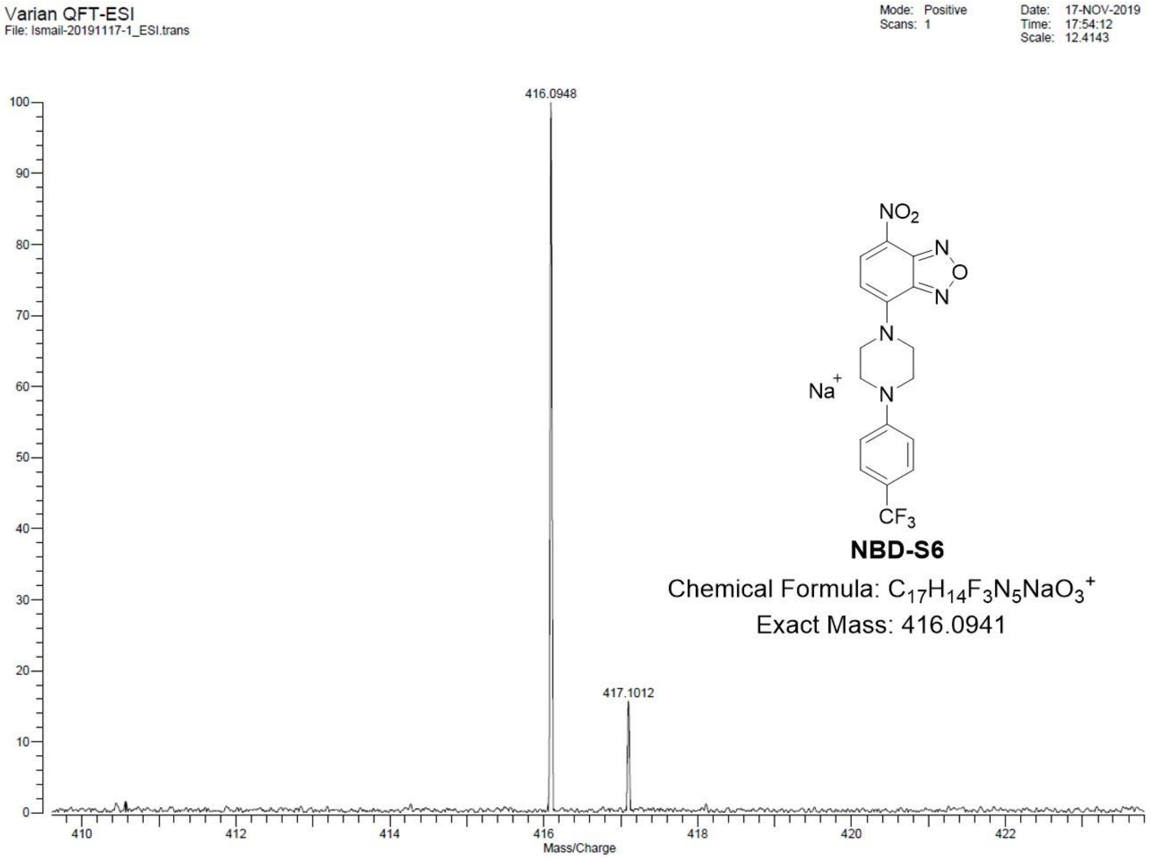
Varian QFT-ESI
File: Ismail-20191117-1_ESI.trans
Mode: Positive
Scans: 1
Date: 17-NOV-2019
Time: 17:54:12
Scale: 12.4143
416.0948
417.1012
Mass/Charge
NBD-S6
Chemical Formula: $C_{17}H_{14}F_3N_5NaO_3^+$
Exact Mass: 416.0941

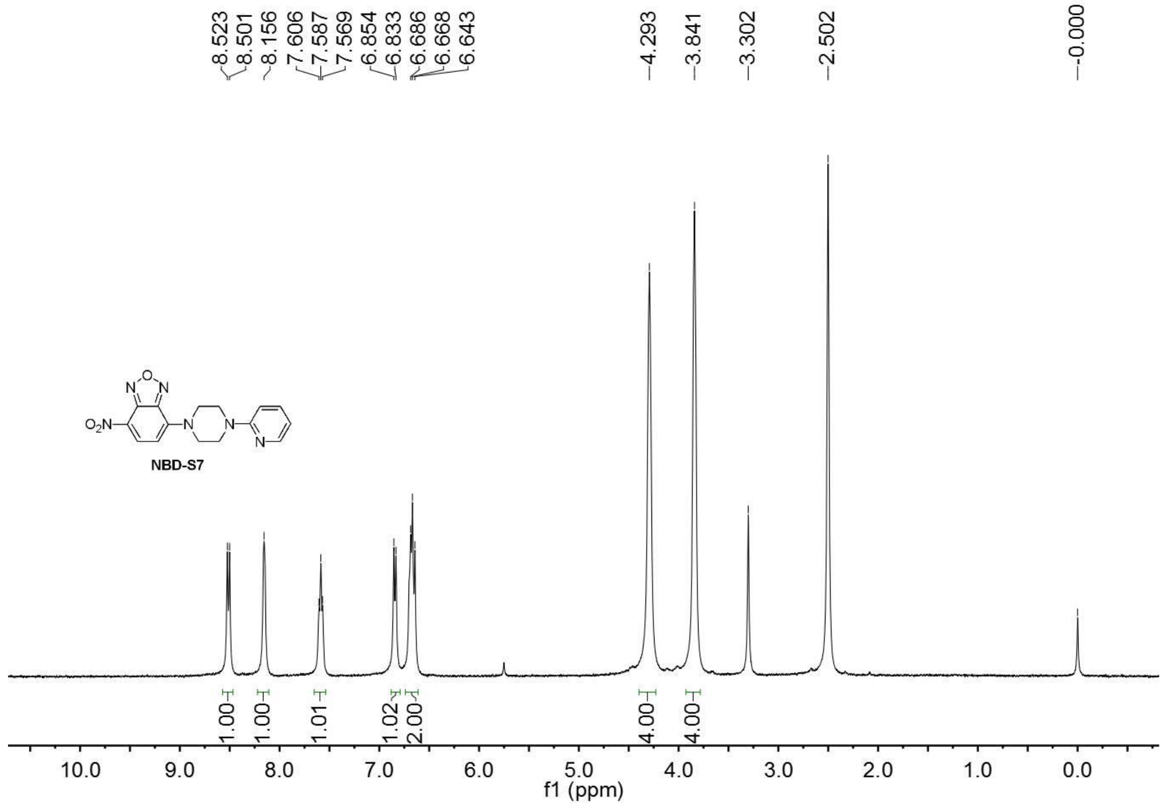
8.523
8.501
8.156
7.606
7.587
7.569
6.854
6.833
6.686
6.668
6.643
4.293
3.841
3.302
2.502
-0.000
NBD-S7
1.00
1.00
1.01
1.02
2.00
4.00
4.00
10.0 9.0 8.0 7.0 6.0 5.0 4.0 3.0 2.0 1.0 0.0
f1 (ppm)

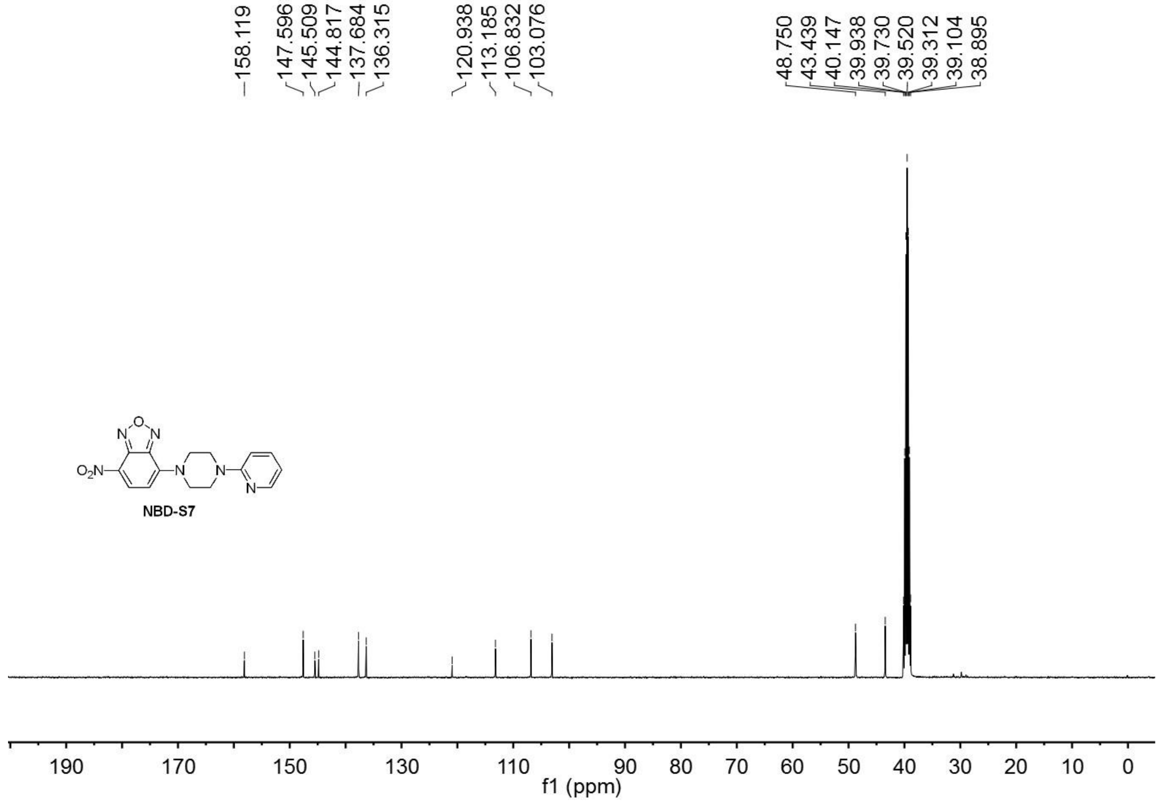
158.119
147.596
145.509
144.817
137.684
136.315
120.938
113.185
106.832
103.076
48.750
43.439
40.147
39.938
39.730
39.520
39.312
39.104
38.895
NBD-S7
190 170 150 130 110 90 80 70 60 50 40 30 20 10 0
f1 (ppm)

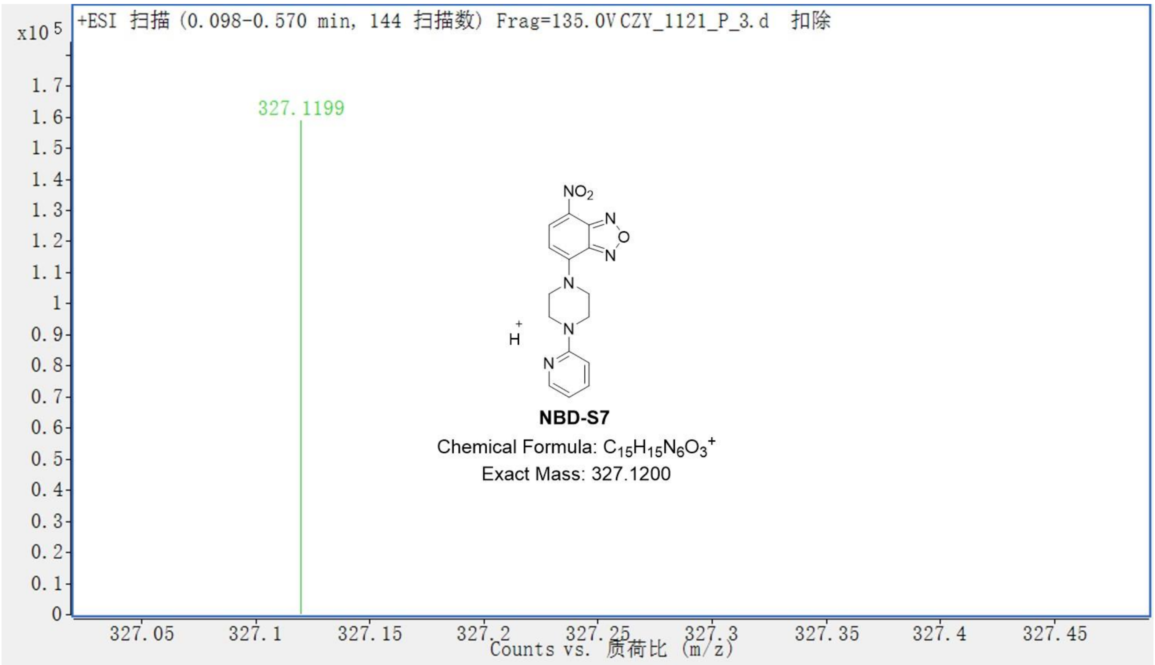
+ESI 扫描 (0.098-0.570 min, 144 扫描数) Frag=135.0V CZY_1121_P_3.d 扣除
x10 5
327.1199
NO2
N
O
N
N
N
H+
N
NBD-S7
Chemical Formula: $C_{15}H_{15}N_6O_3^+$
Exact Mass: 327.1200
Counts vs. 质荷比 (m/z)

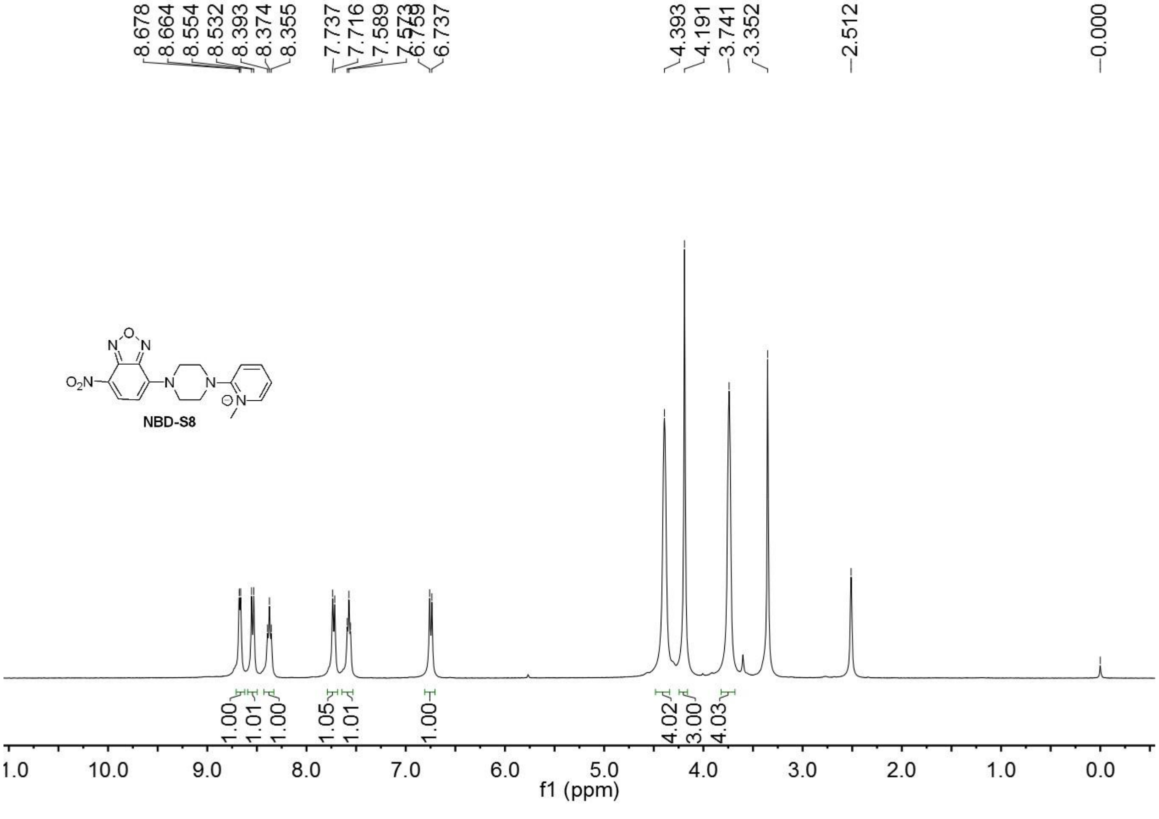
8.678
8.664
8.554
8.532
8.393
8.374
8.355
7.737
7.716
7.589
7.573
6.759
6.737
4.393
4.191
3.741
3.352
2.512
0.000
NBD-S8
1.00
1.01
1.00
1.05
1.01
1.00
4.02
3.00
4.03
f1 (ppm)

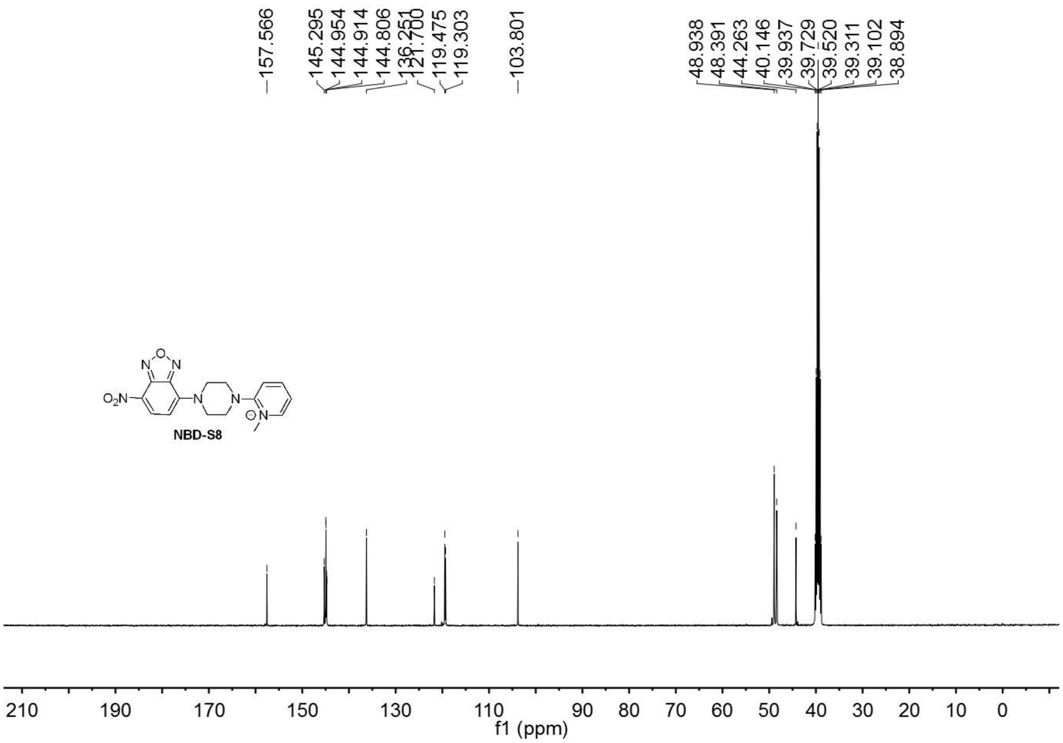
157.566
145.295
144.954
144.914
144.806
136.251
121.700
119.475
119.303
103.801
48.938
48.391
44.263
40.146
39.937
39.729
39.520
39.311
39.102
38.894
NBD-S8
210
190
170
150
130
110
90
80
70
60
50
40
30
20
10
0
f1 (ppm)

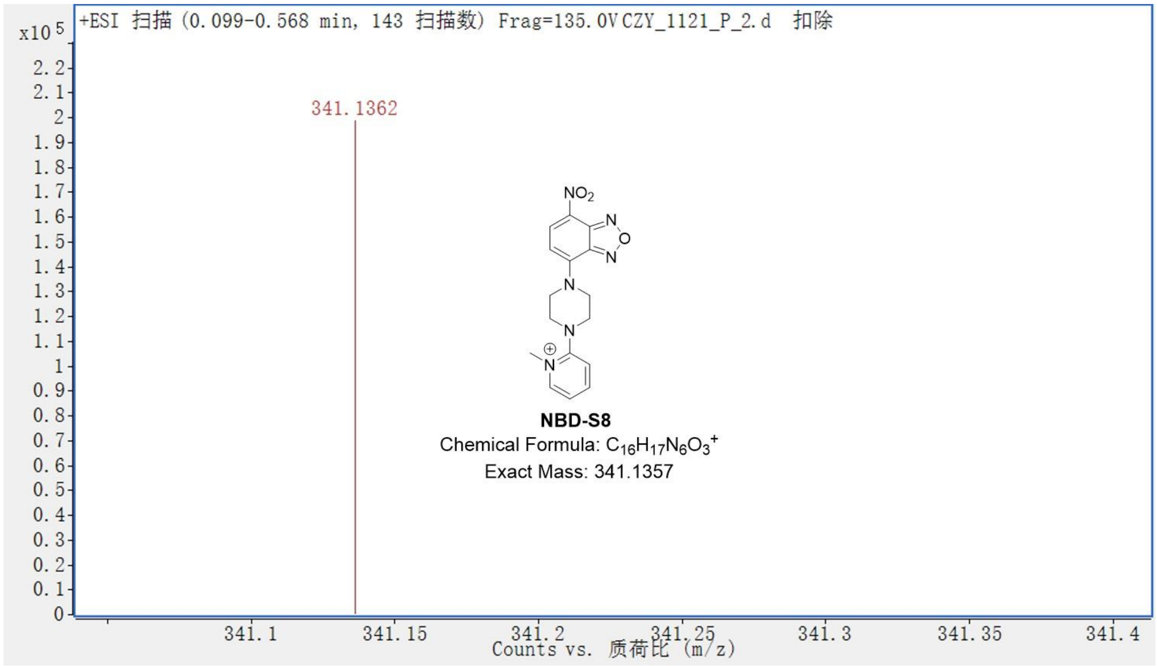
+ESI 扫描 (0.099-0.568 min, 143 扫描数) Frag=135.0V CZY_1121_P_2.d 扣除
x10 5
341.1362
NBD-S8
Chemical Formula: C16H17N6O3+
Exact Mass: 341.1357
341.1
341.15
341.2
341.25
341.3
341.35
341.4
Counts vs. 质荷比 (m/z)

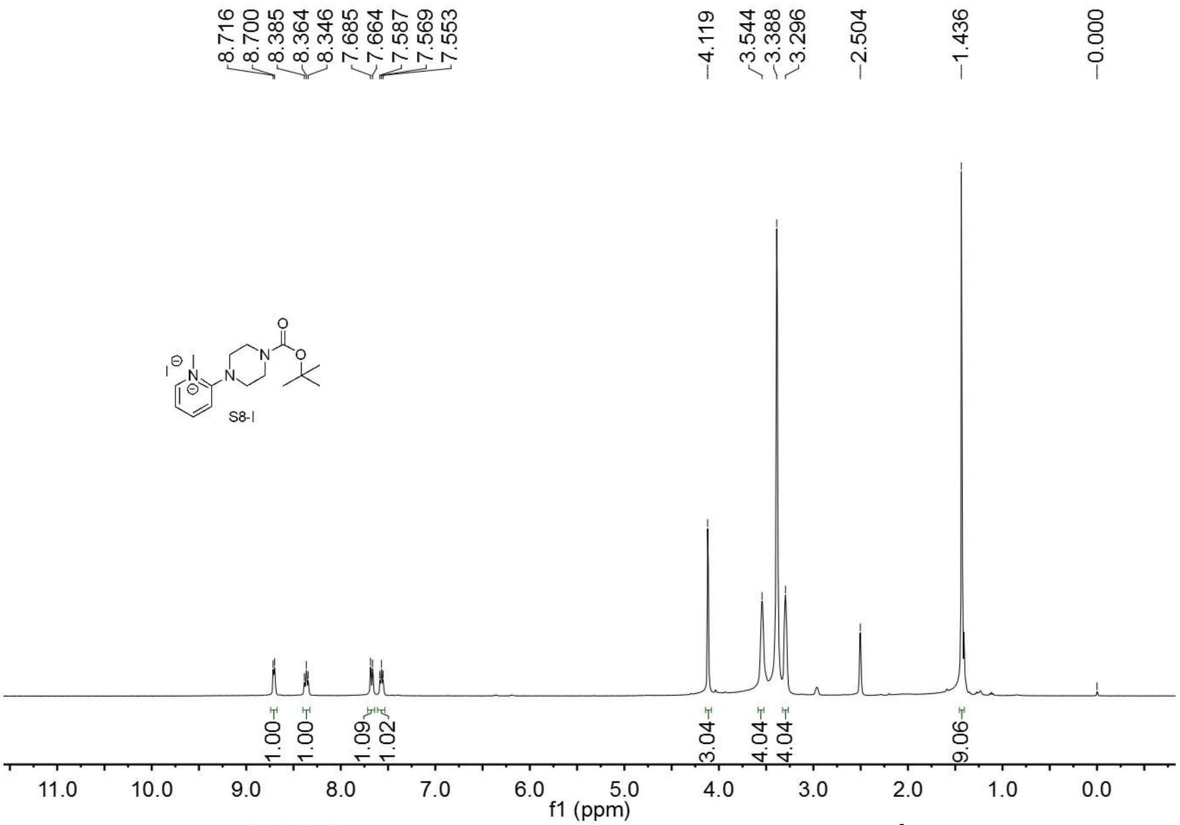
8.716
8.700
8.385
8.364
8.346
7.685
7.664
7.587
7.569
7.553
4.119
3.544
3.388
3.296
2.504
1.436
0.000
S8-I
1.00
1.00
1.09
1.02
3.04
4.04
4.04
9.06
11.0 10.0 9.0 8.0 7.0 6.0 5.0 4.0 3.0 2.0 1.0 0.0
f1 (ppm)

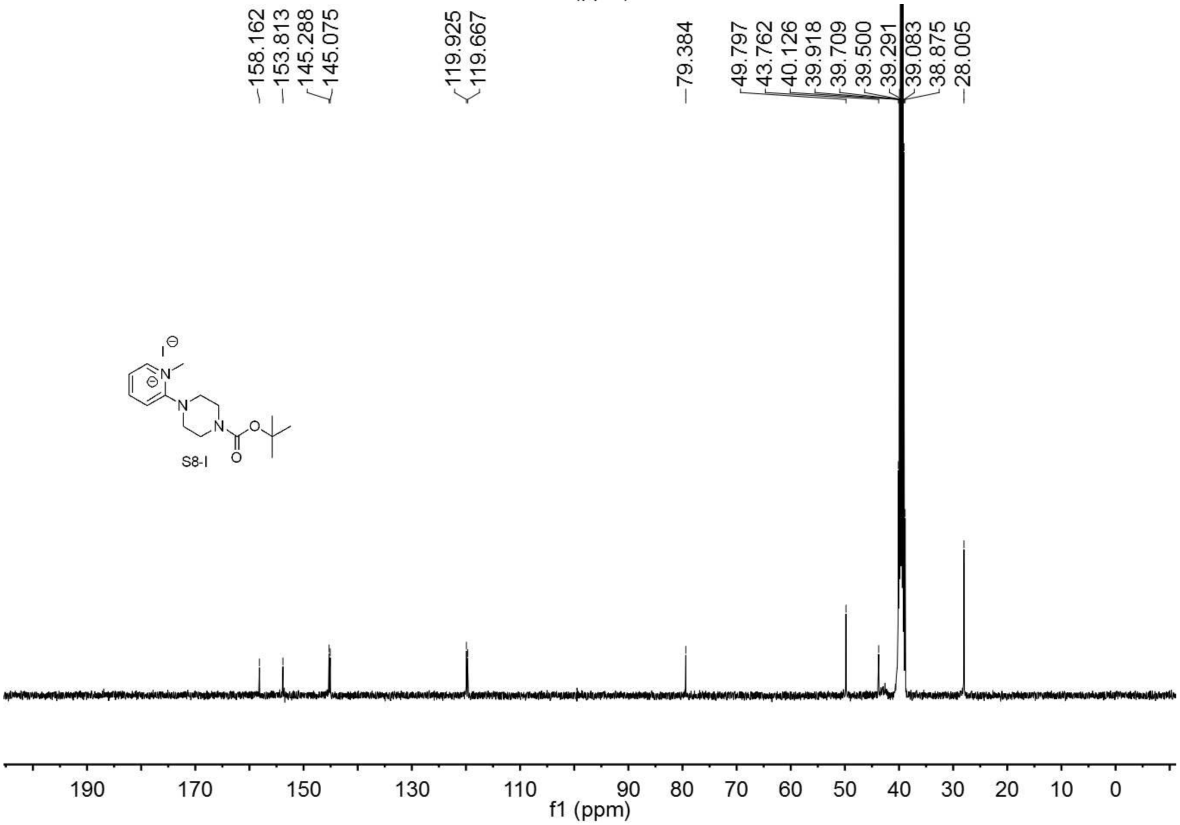
158.162
153.813
145.288
145.075
119.925
119.667
79.384
49.797
43.762
40.126
39.918
39.709
39.500
39.291
39.083
38.875
28.005
S8-I
190 170 150 130 110 90 80 70 60 50 40 30 20 10 0
f1 (ppm)

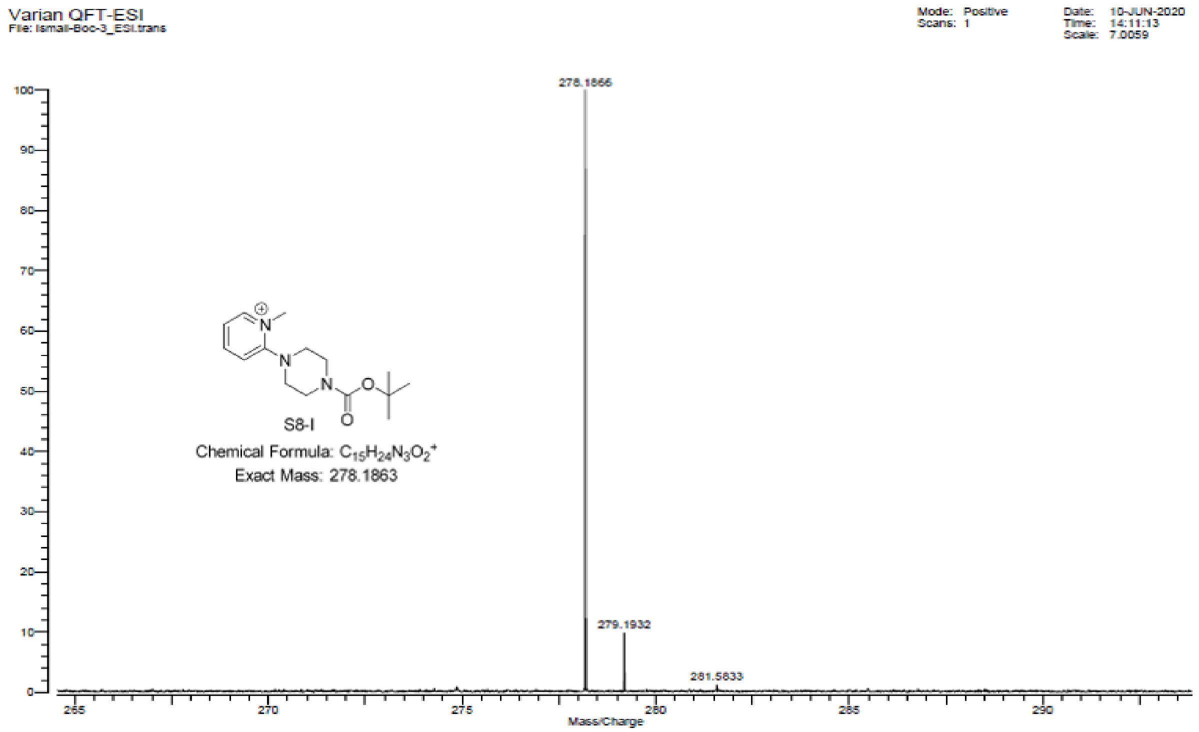

Varian QFT-ESI
File: Ismail-Boc-3_ESI.trans
Mode: Positive
Scans: 1
Date: 10-JUN-2020
Time: 14:11:13
Scale: 7.0059
278.1866
279.1932
281.5833
S8-I
Chemical Formula: C15H24N3O2+
Exact Mass: 278.1863
Mass/Charge

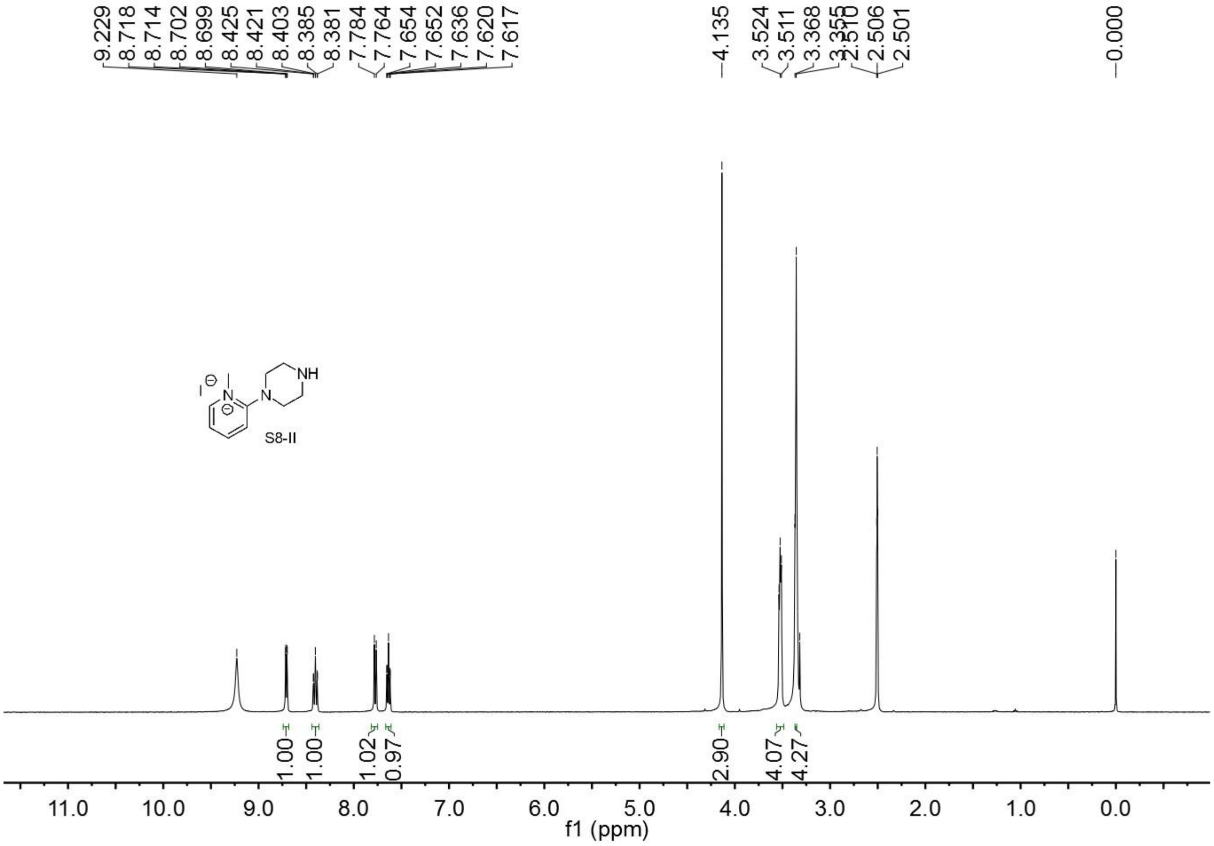

9.229
8.718
8.714
8.702
8.699
8.425
8.421
8.403
8.385
8.381
7.784
7.764
7.654
7.652
7.636
7.620
7.617
4.135
3.524
3.511
3.368
3.355
2.510
2.506
2.501
0.000
S8-II
1.00
1.00
1.02
0.97
2.90
4.07
4.27
f1 (ppm)

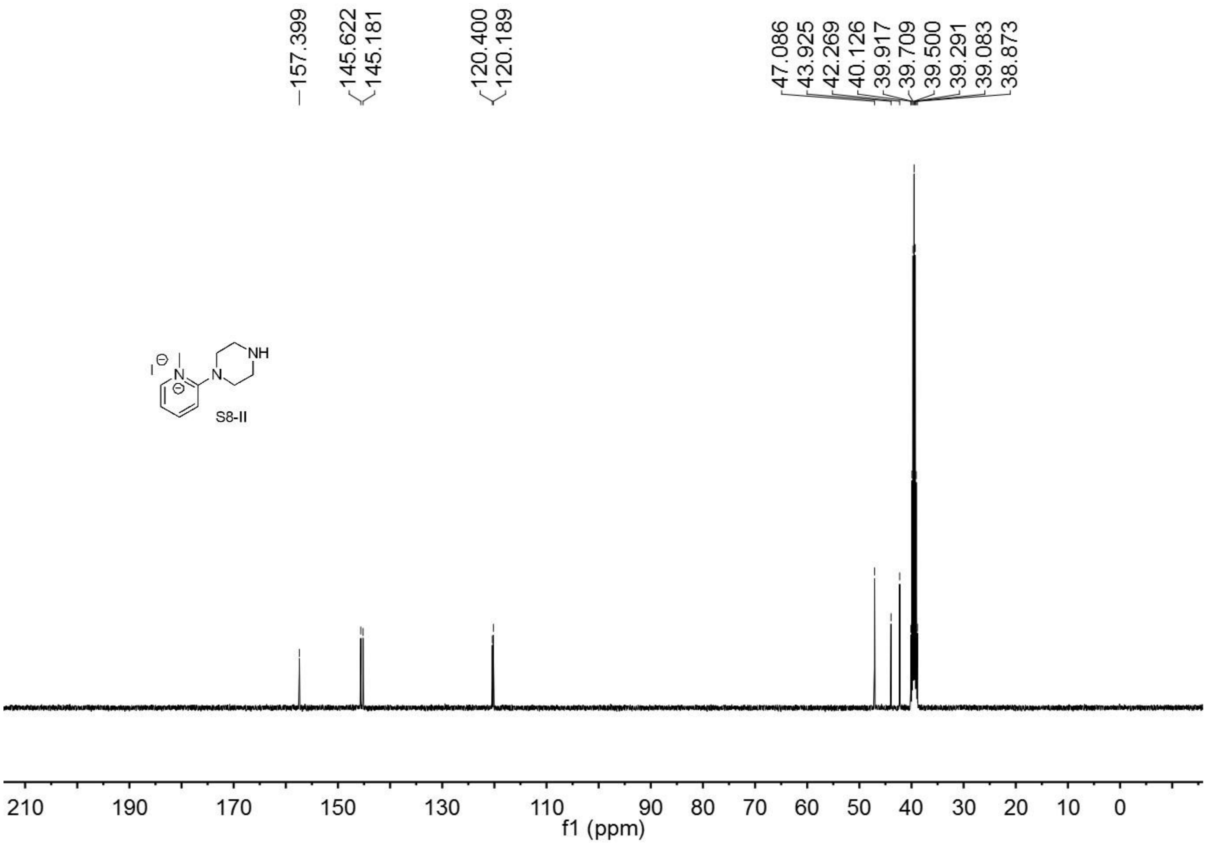

157.399
145.622
145.181
120.400
120.189
47.086
43.925
42.269
40.126
39.917
39.709
39.500
39.291
39.083
38.873
NH
N
I
S8-II
210
190
170
150
130
110
90
80
70
60
50
40
30
20
10
0
f1 (ppm)

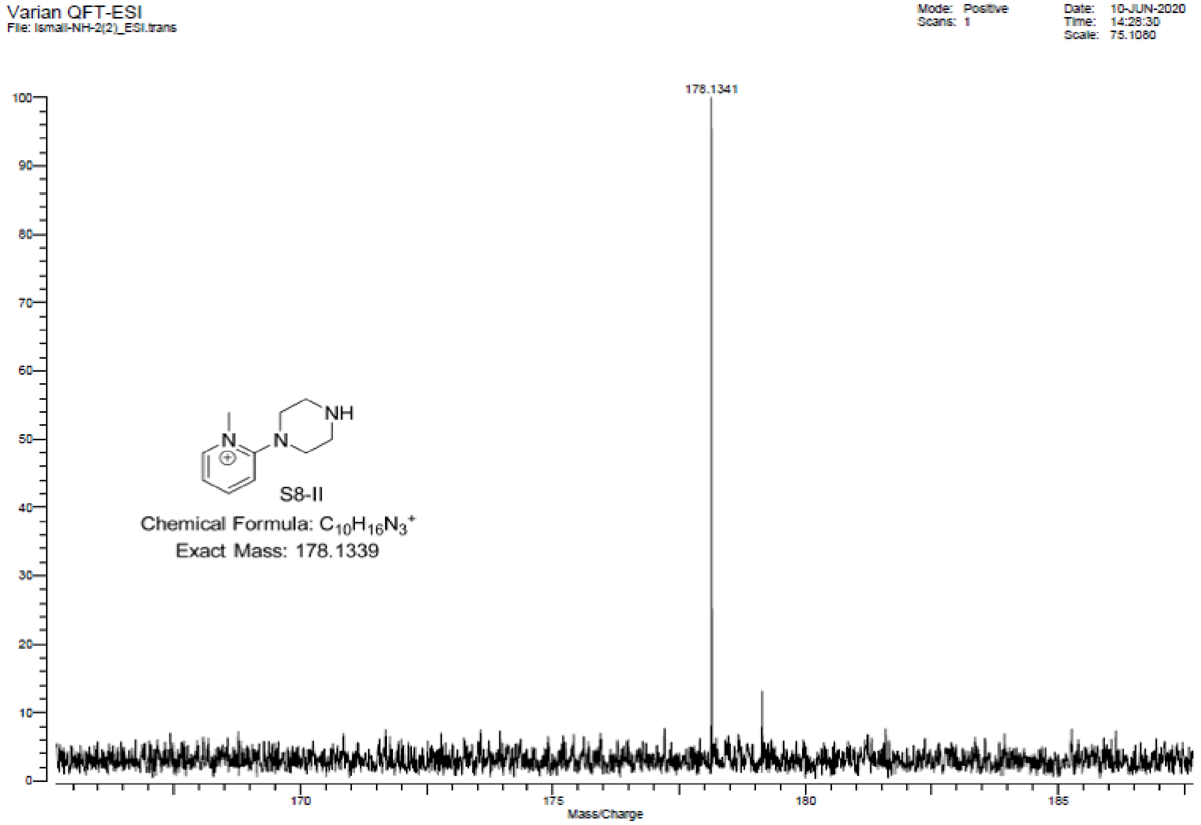

Varian QFT-ESI
File: Ismail-NH-2(2)_ESI.trans
Mode: Positive
Scans: 1
Date: 10-JUN-2020
Time: 14:28:30
Scale: 75.1080
178.1341
NH
N
S8-II
Chemical Formula: $C_{10}H_{16}N_3^+$
Exact Mass: 178.1339
100
90
80
70
60
50
40
30
20
10
0
170
175
180
185
Mass/Charge

9. Supporting reference

1. F. Song, Z. Li, J. Li, S. Wu, X. Qiu, Z. Xi and L. Yi, *Org. Biomol. Chem.*, 2016, **14**, 11117-11124.
2. N. Mrsic, L. Lefort, J. A. F. Boogers, A. J. Minnaard, B. L. Feringa and J. G. de Vriesa, *Adv. Synth. Catal*., 2008, **350**, 1081-1089.
3. J. T. Bowler, F. M. Wong, S. Gronert, J. R. Keeffe and W. Wu, *Org. Biomol. Chem.*, 2014, **12**, 6175-6180.
4. C. Wei, R. Wang, L. Wei, L. Cheng, Z. Li, Z. Xi and L. Yi, *Chem. Asian. J.*, 2014, **9**, 3586-3592.
5. O. V. Dolomanov, L. J. Bourhis, R. J. Gildea, J. A. K. Howard and H. Puschmann, *J. Appl. Cryst*., 2009, **42**, 339-341.
6. G. M. Sheldrick, *Acta Cryst. A.*, 2015, **71**, 3-8.
7. M. M. Cerda, Y. Zhao and M. D. Pluth, *J. Am. Chem. Soc.*, 2018, **140**, 12574-12579.
8. T. D. Newton and M. D. Pluth, *Chem. Sci.*, 2019, **10**, 10723-10727.
9. L. A. Montoya, T. F. Pearce, R. J. Hansen, L. N. Zakharov and M. D. Pluth, *J. Org. Chem.*, 2013, **78**, 6550-6557.
10. K. Zhang, J. Zhang, Z. Xi, L.Y. Li, X. Gu, Q. Z. Zhang and L. Yi, *Chem. Sci.*, 2017, **8**, 2776-2781.
11. S. K. Bae, C. H. Heo, D. J. Choi, D. Sen, E. H. Joe, B. R. Cho and H. M. Kim, *J. Am. Chem. Soc.*, 2013, **135**, 9915-9923.
